# Formal synthesis of (+)-lactacystin from l-serine†

**DOI:** 10.1039/c9ra07244f

**Published:** 2019-09-24

**Authors:** Philip C. Bulman Page, Ross L. Goodyear, Yohan Chan, Alexandra M. Z. Slawin, Steven M. Allin

**Affiliations:** School of Chemistry, University of East Anglia, Norwich Research Park Norwich Norfolk NR4 7TJ UK p.page@uea.ac.uk; School of Chemistry, University of St Andrews St Andrews Scotland KY16 9ST UK; School of Science & Technology, Nottingham Trent University Clifton Nottingham NG11 8NS UK

## Abstract

A formal, stereocontrolled synthesis of lactacystin has been completed from *t*-Bu*-O-*l-serine, providing the key intermediate 13, also useful for the generation of a range of C-9 analogues.

## Abstract 2

A formal, stereocontrolled synthesis of lactacystin has been completed from *t*-Bu-*O*-l-serine.

## Graphical abstract

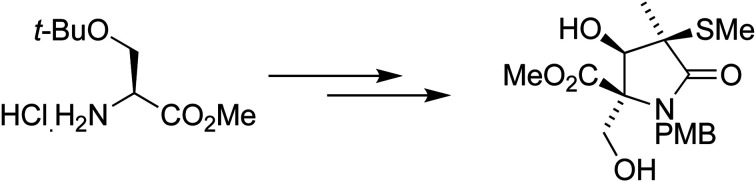

## Introduction

The 20S proteasome is a large barrel-shaped protein comprised of 28 subunits.^1^ The primary function of the proteasome involves the degradation of damaged proteins, a vital component of the ubiquitin proteasome pathway. Inhibition of the proteasome can lead to cell death. This property of the proteasome has made it a promising target for cancer therapeutics.^2^

**Fig. 1:**
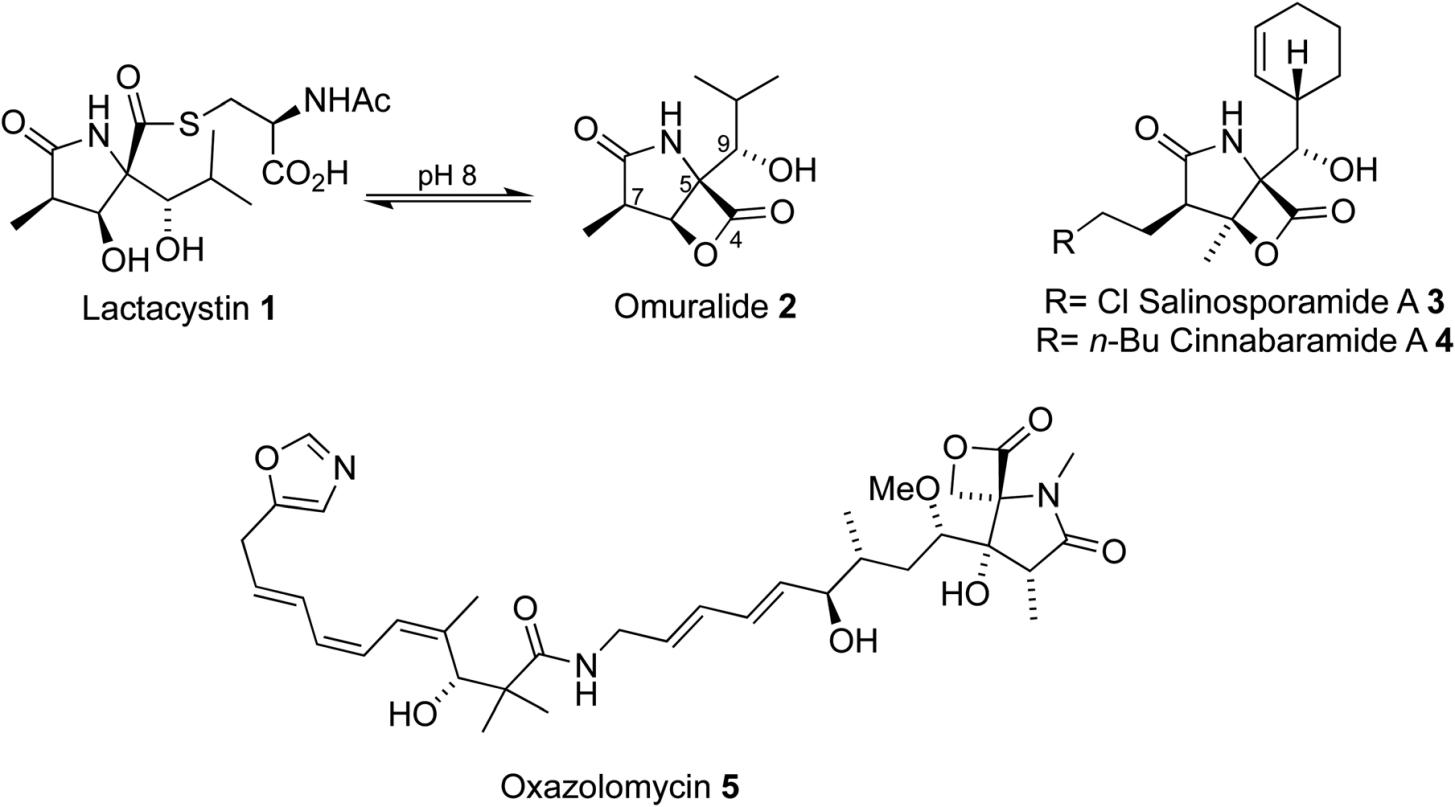
Examples of proteasome inhibitors.

Microbial metabolites have provided a wealth of proteasome inhibitors (Fig. 1). Lactacystin 1 was discovered in 1991 by Ōmura through extraction from the cultured broth of *Streptomyces* sp OM-6519,^3,4^ after observations that it induced differentiation of the mouse neuroblastoma cell line, a consequence of proteasome inhibition. Further studies, driven by several early efforts to prepare lactacystin and analogues, found that lactacystin undergoes cyclization to the β-lactone omuralide 2, which inhibits the proteasome^5^ and can induce apoptosis. The potential for beta-lactone, gamma-lactam proteasome inhibitors was further highlighted by the discovery of the salinosporamides, *e.g.*3,^6–8^ and cinnabaramides, *e.g.*4.^9^ Similar lactam cores have also been discovered in the metabolite oxazolomycin^10^5, which possesses antibiotic activity. A number of strategies to access these cores to produce natural products and analogues of high therapeutic value have been reported.^11^

## Results & discussion

**Scheme 1:**
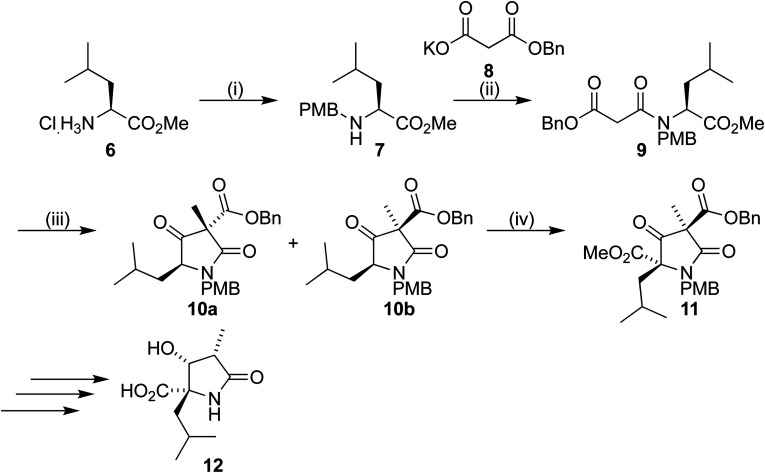
Reagents and conditions: (i) *p*-methoxybenzaldehyde, Et_3_N, MeOH, NaBH_4_, 71%; (ii) NMM, EDAC·HCl, DMAP, CH_2_Cl_2_, r.t., overnight, 93%; (iii) TBAF, THF, r.t., 0.5 h, then MeI, 0 °C to r.t., overnight, 10a/b 57%; (iv) LiHMDS, DMPU, THF, −78 °C, 0.5 h, then NCCO_2_Me, −78 °C, 4 h, 70%.

Our synthetic work has focused on the development of a functionalized lactam core from glycine,^12,13^ and recently using l-leucine 6 as a starting material to produce a formal synthesis of the C9-deoxyomuralide analogue, using the natural chirality of the amino acid to direct the synthesis.^14^ Formation of the carbon skeleton of omuralide was achieved in 4 steps (Scheme 1). Peptide coupling of PMB-protected leucine 7 to the malonic acid benzyl ester 8 provided the precursor 9 to the key Dieckmann cyclization/alkylation step. Cyclization was induced with TBAF, and subsequent addition of methyl iodide provided two diastereoisomers. The major, 10b, was isolated and used in an acylation using Mander's reagent to give 11, thus completing the carbon skeleton. Six further steps produced pyroglutamate 12, which can be cyclized to give 9-deoxyomuralide in one step.

With this methodology in place, we turned our attention to a serine-derived route to lactacystin. This would provide a hydroxy group in the C9 position, which previous SAR studies have shown to be key for effective proteasome inhibition.

**Scheme 2:**
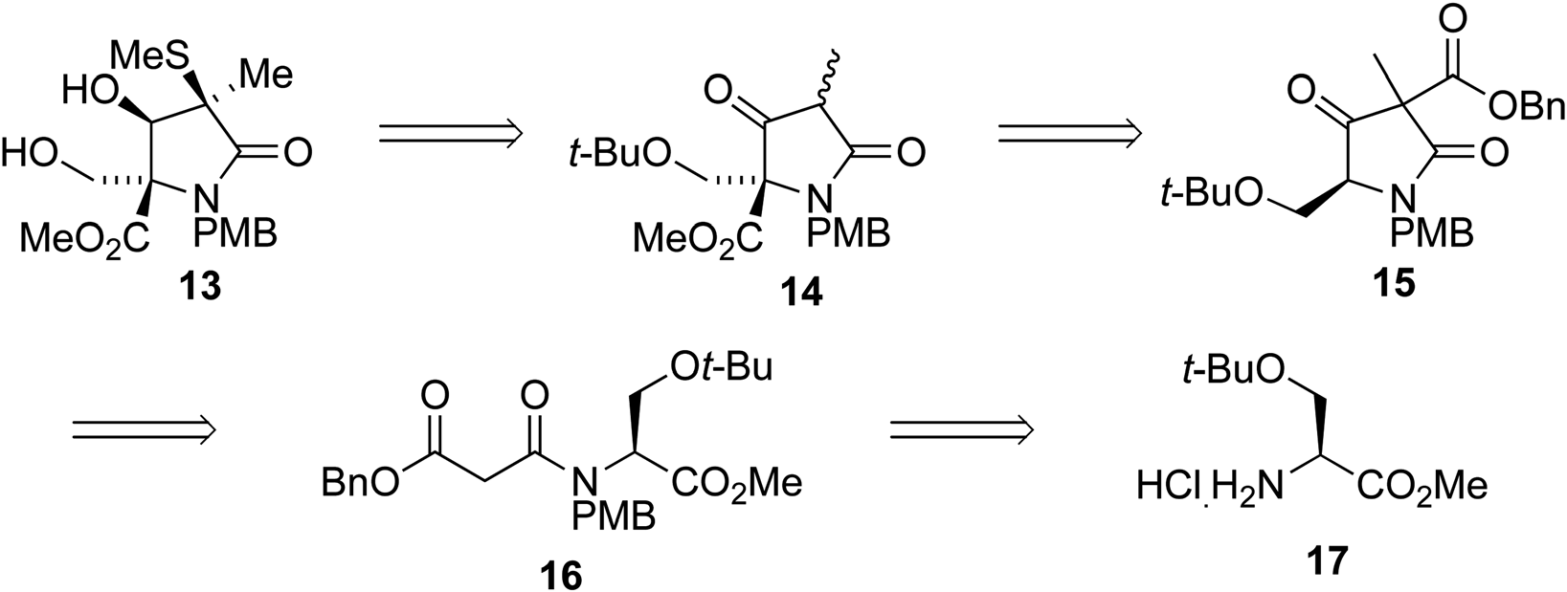


We envisaged that intermediate 13 could be synthesized from a suitably protected tetramic acid-like core 14 (Scheme 2). A removable benzyl ester would be used to help direct acylation of lactam 15 using Mander's reagent; 15 could in turn be formed using the cyclization/alkylation procedure previously developed. The Dieckmann cyclization precursor 16 could be synthesized from peptide coupling of a suitably protected *O-t*-Bu-l-serine 17. This starting material was used due to the size of the *t*-butyl group, its tolerance towards a wide range of conditions, and because both enantiomers are commercially available.

**Scheme 3:**
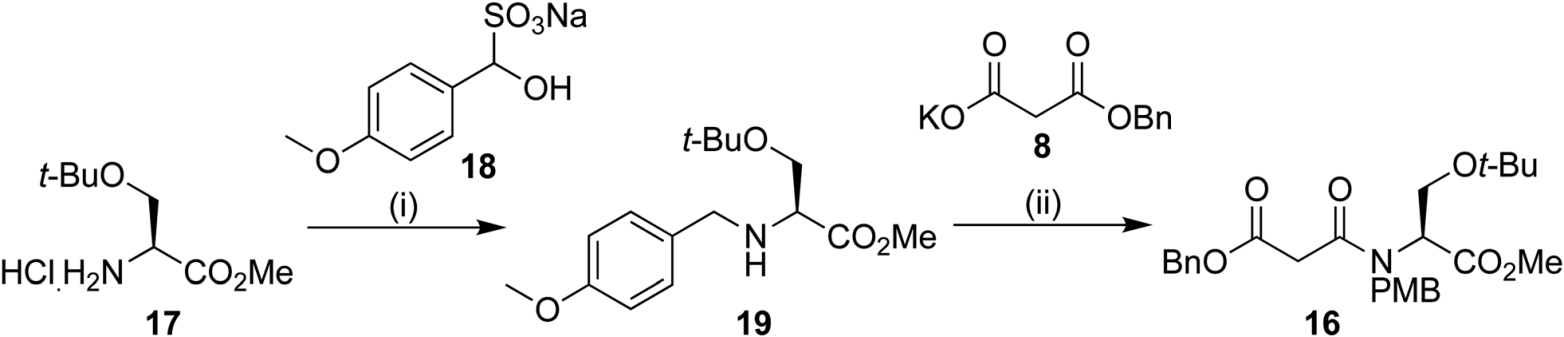
Reagents and conditions: (i) 14, NaBH_3_CN, Et_3_N, MeOH 0 °C 16 h (ii) 8, EDAC·HCl, *N*-methyl morpholine, DMAP, DCM, 16 h; 49% over the 2 steps.

PMB protection of the serine derivative 17 was carried out using a modification of a procedure by Vázquez using the PMB sulphite adduct 18.^15^ We have previously observed^14^ epimerization during imine formation, and so a one-pot procedure was developed. After work up, the protected serine 19 could be used without further purification. Peptide coupling to benzyl malonic ester 8 provided the Dieckmann cyclization precursor in 49% yield over the two steps. Analysis using chiral stationary phase HPLC showed that only negligible epimerization had occurred at this point, providing 16 in 97% ee (Scheme 3).

**Scheme 4:**
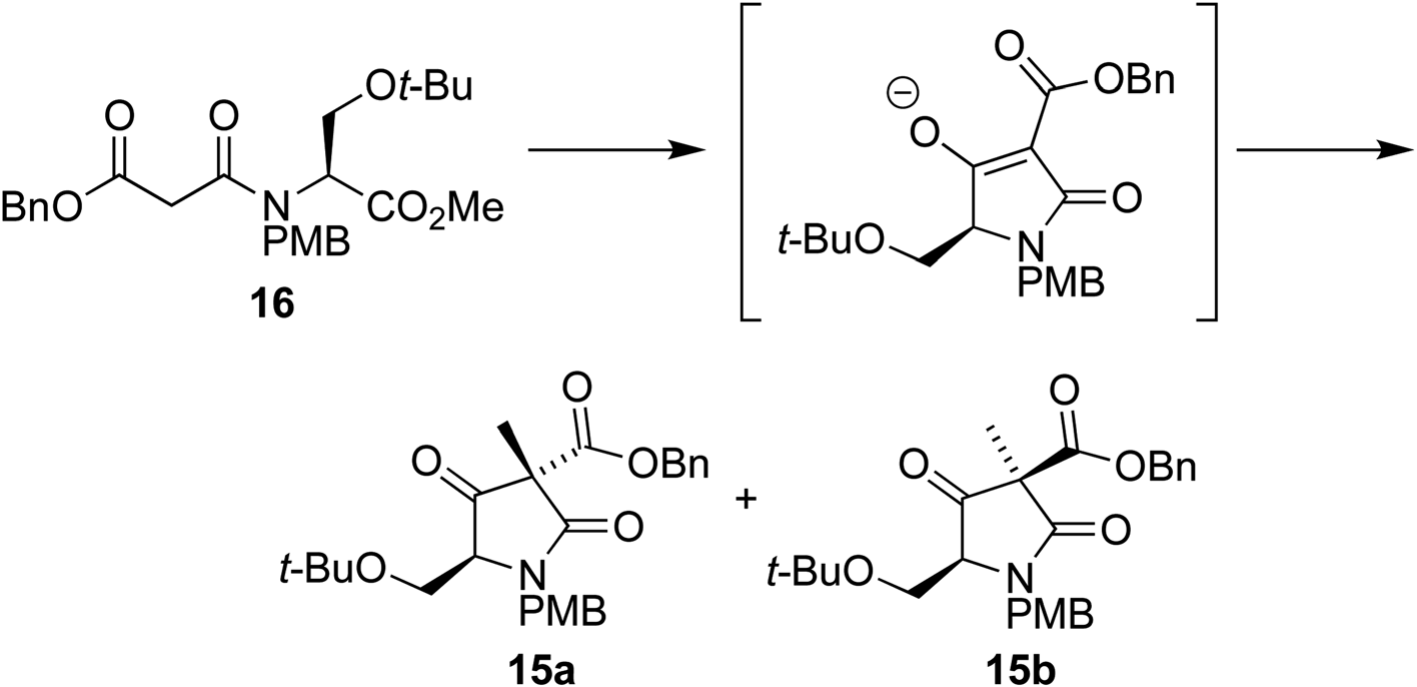


With the Dieckmann precursor 16 in hand, we turned our attention to the tandem cyclization/alkylation step (Scheme 4). When subjected to the conditions used previously (TBAF in THF, then MeI addition), the cyclization/alkylation proceeded with good yield and provided a 10 : 1 ratio of diastereoisomers 15a and 15b, in favour of 15a, where the newly added methyl group is situated on the same face of the molecule as the *t*-BuO group.

**Scheme 5:**
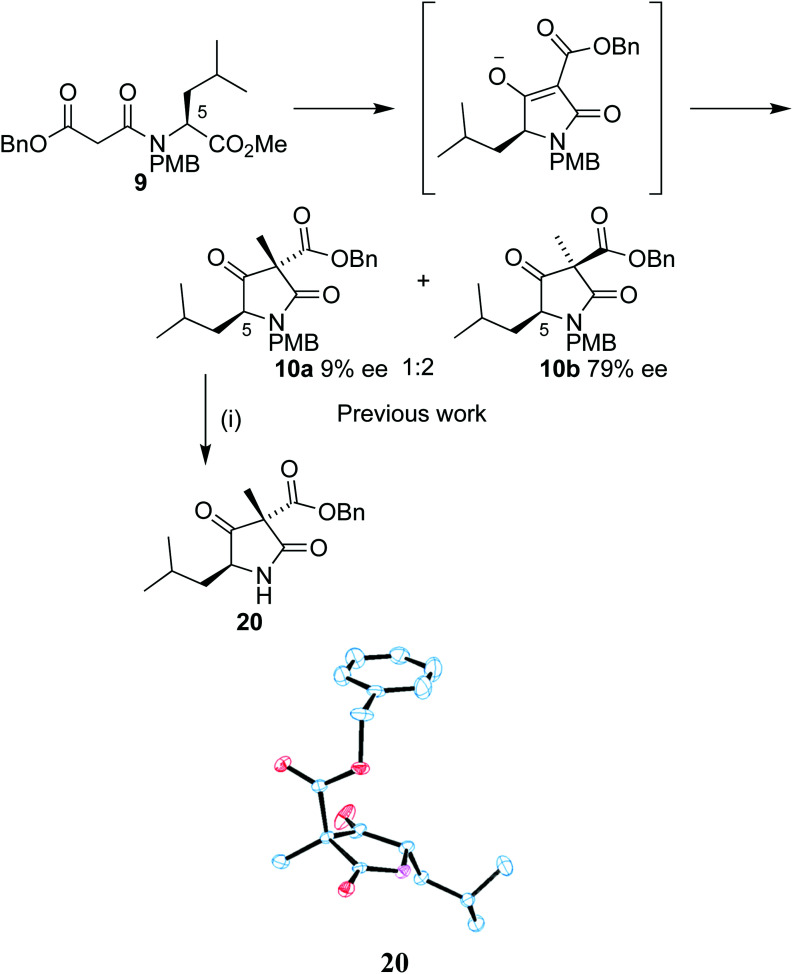
Reagents and conditions: (i) CAN, 3 : 1 MeCN/H_2_O, 84%.

Our previous work on the leucine analogue^14^ showed that addition of the methyl iodide is in that case preferentially introduced opposite the isobutyl moiety (Scheme 5). This outcome was expected due to the bulky nature of the amino acid group and the assumed planarity of the intermediate. The diastereoisomers were isolated in a 1 : 2 mixture and the observation was confirmed by single crystal X-ray analysis of 20, derived from the minor diastereoisomer 10a by PMB removal.

We were therefore surprised to find that our serine derivative afforded the diastereoisomers in a 10 : 1 mixture favouring the diastereoisomer 15a with the methyl group *cis* to the *t*-BuO group, presumably because in forming 15b the benzyl ester unit is forced nearer to the *t*-BuO, so raising the transition state energy of that pathway.

On analysis by chiral HPLC the major diastereoisomer was found to have a disappointing ee of 44%. Running the reaction at decreased temperatures provided higher ee at the expense of yield. Further optimization (Table 1), including increased reaction times, lower temperatures and the separation of the cyclization and alkylation steps, achieved ees of up to 79% and yields of 66%. The ee could be improved further through recrystallization from isopropanol, which produced 97% ee. We suspect that the decrease in ee is primarily the result of epimerization at the C5 position in the mixture of diastereoisomers 15a/b following alkylation, leading to the (presumed) more stable diastereoisomer 15a. Lower temperatures reduce the degree of this epimerization, leading to the decreased diastereoselectivities (3 : 1) but higher ees.

**Table 1:** Optimization of the Dieckmann cyclization/alkylation giving 15a

Entry	Conditions	Yield	Ratio of diastereoisomers 15a : 15b	ee of 15a^a^
1	TBAF, THF 2 h then MeI 2 h	62%	10 : 1	44%
2	TBAF, THF 0.5 h, MeI, 0 °C to r.t. over 16 h	42%	10 : 1	60%
3	TBAF, THF 0.5 h, MeI, −10 °C	Trace	—	76%
4	TBAF, ether, 5 m, THF MeI, −15 °C, 64 h	30%	3 : 1	76%
5	TBAF, ether, 5 m, THF MeI, −12 °C, 64 h	66%	3 : 1	79%

a Determined by HPLC on chiral stationary phase by comparison with racemic material using Chiralpak AD-H or Knauer Eurocel 01 columns.

During our work with the l-leucine derived analogue 9, we were able to isolate a 1 : 2 ratio of diastereoisomers 10a and 10b in 9% ee and 79% ee respectively (Scheme 5).^14^ Our results with leucine strongly indicate that the partial racemization occurs after the alkylation. If racemization occurred solely before alkylation, the ratio of diastereoisomers in the racemic material should match the ratio of diastereoisomers in the enantiopure material. As we observed the diastereoisomers in a 1 : 2 ratio at 9% and 79% ee respectively, the vast majority of racemization must occur after alkylation. Due to the structural similarity between our l-leucine-derived lactams and our serine-derived lactams, it seems likely that the mode of racemization is analogous.

**Scheme 6:**
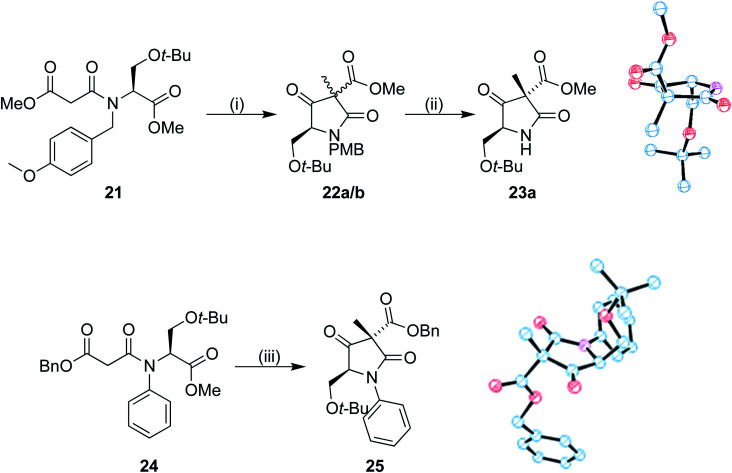
Reagents and conditions: (i) TBAF, ether, 5 m, THF, MeI, −12 °C, 58 h; 87% (ii) CAN, 3 : 1 MeCN/H_2_O, 57%, (iii) TBAF, ether, 5 m, THF MeI, −12 °C, 64 h; 11%.

To investigate the stereoselectivity further, the benzyl ester was replaced by a methyl counterpart (Scheme 6). Coupling of 19 to the half malonic methyl ester potassium salt in an analogous procedure to that of 16 proceeded in good yield, to produce 21. Once treated with our cyclization/alkylation procedure, NMR analysis showed that the cyclization occurred efficiently, but we found that 22a/b decomposed if left in contact with silica gel for extended periods of time. Partial purification was therefore completed with a silica plug to produce a 3 : 1 mixture of diastereoisomers; the mixture was treated with CAN removing the PMB group (Scheme 6). The purified major isomer 23a was obtained as a colourless crystalline solid. Analysis by single crystal X-ray diffraction showed that the methyl group was still preferentially added to the same face as the *tert*-butoxy group, allowing us to conclude that the benzyl ester was not the primary influence on diastereoselectivity in our system.

The system was further investigated by replacing the PMB group with a phenyl (Scheme 6). Chan–Lam coupling of the serine derivative with phenyl boronic acid and subsequent peptide coupling to the benzyl malonic half ester 8 produced the Dieckmann cyclization precursor 24. Compound 24 was subjected to our cyclization conditions. The methylated product 25 was produced in a 5 : 1 ratio of diastereoisomers according to the ^1^H NMR spectrum, albeit in a low yield, perhaps due to the conformation required for cyclization being more unfavourable. A crystal of the major diastereoisomer suitable for X-ray analysis was obtained. Once again, in the major product the methyl group had been introduced to the same face as the *tert*-butoxy.

**Scheme 7:**
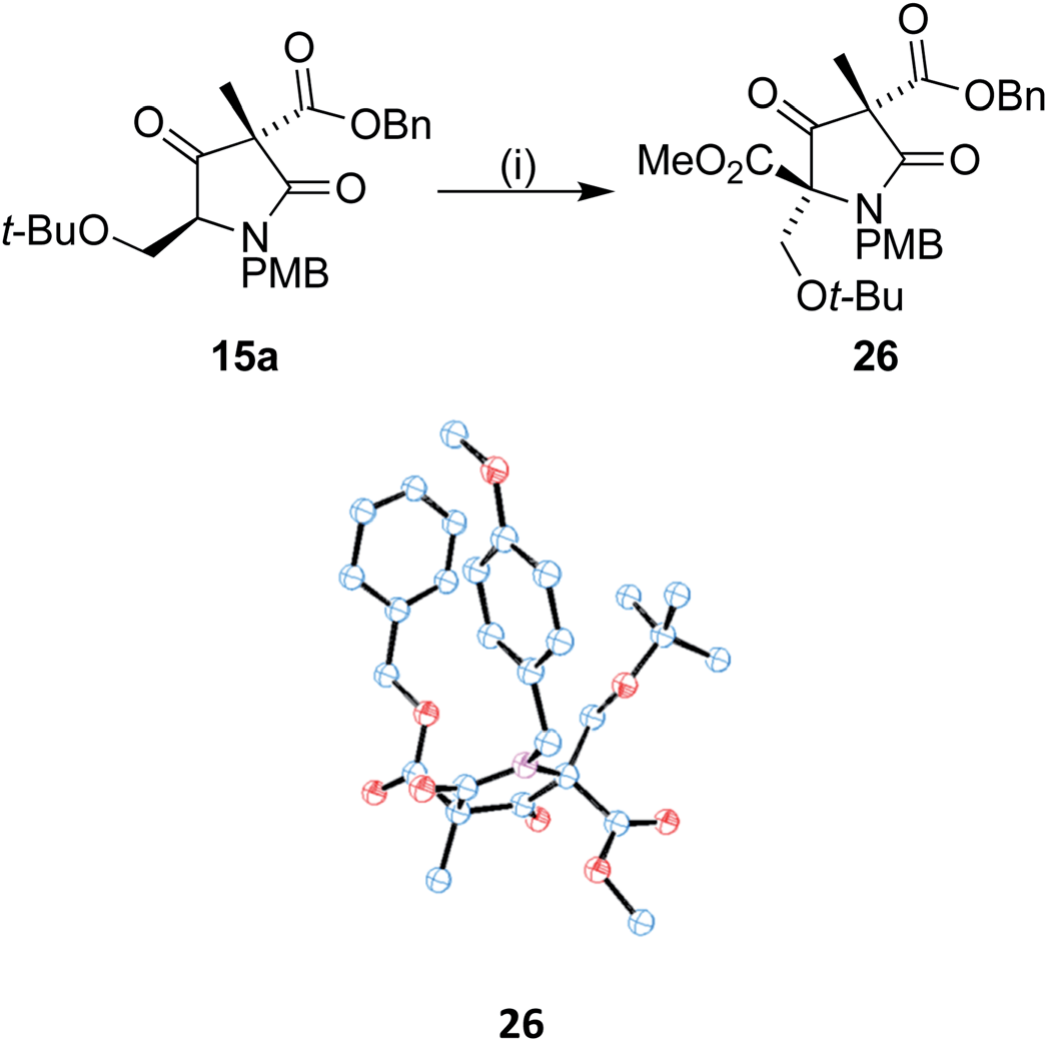
Reagents and conditions: (i) LiHMDS, DMPU, THF, −40 °C, 0.5 h then MeOCOCN, 3 h, −40 °C; 69%.

With 15a in hand, we sought to install the methyl ester moiety that would eventually form the beta lactone found in omuralide. Acylation using Mander's reagent at −40 °C was found to produce compound 26 in good yield with no observed *O*-acetylation. In addition, only one diastereoisomer could be observed (Scheme 7). A small drop in ee was observed during the acylation, to 69%, perhaps due to the presence of small quantities of the minor diastereomer 15b. Using recrystallized 15a, the ee of 26 was 84%. Single crystal X-ray analysis of 26 was carried out on both the racemic and enantiomerically pure (obtained by recrystallization) forms, the latter confirming that the absolute stereochemistry of our C9 centre is as found in (+)-lactacystin.

**Scheme 8:**
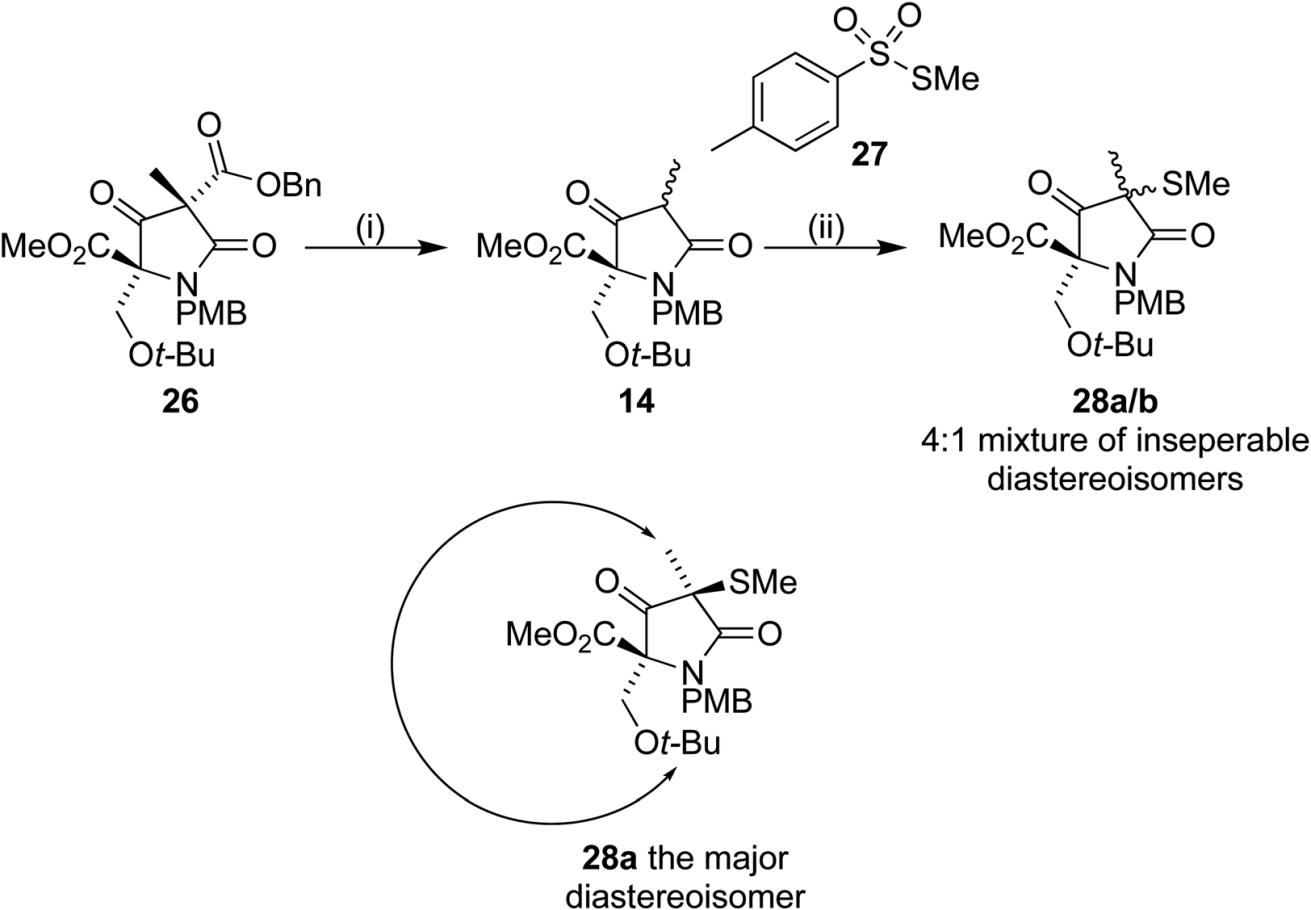
Reagents and conditions (i) Pd(OH)_2_/C, H_2_, 35 °C, 16 h (ii) 27, Et_3_N, DCM, 4 h (70% over 2 steps).

Removal of the benzyl ester from 26 by hydrogenolysis led to tetramic acid derivative 14 as a mixture of diastereoisomers. The mixture proved unstable towards silica gel and so was used in the next step without purification. On treatment with *S*-methyl *p*-toluenethiosulfonate 27, in a similar manner to Pattenden,^16,17^ a 4 : 1 mixture of inseparable diastereoisomers 28a/b was formed. NOESY experiments confirmed that the major observed diastereoisomer was the desired one, with the methyl thioether *trans* to the *t*-BuO group (Scheme 8).

**Scheme 9:**
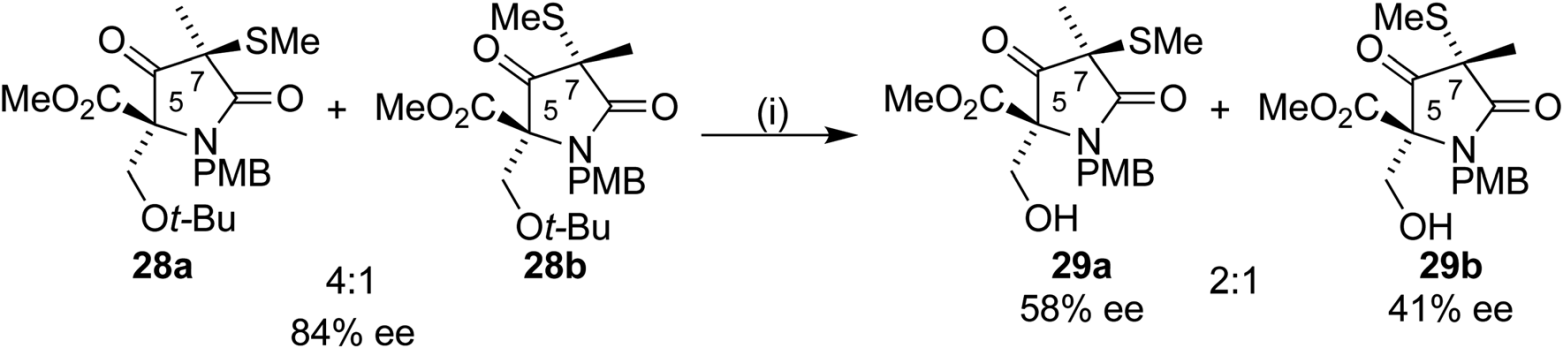
Reagents and conditions: (i) TFA/DCM 1 : 1, 1.5 h; 76%.

Removal of the *t*-butyl group from 28a would allow completion of the formal synthesis to form the Corey intermediate 29a.^18^ Treatment of 28a/b with a 1 : 1 TFA/DCM mixture led to an inseparable mixture of the diastereoisomers 29a/b in a 2 : 1 mixture after purification by column chromatography. To our surprise, analysis by chiral HPLC showed a drop in ee from 84% to 58% and 41% respectively for 29a and 29b (Scheme 9).

**Scheme 10:**
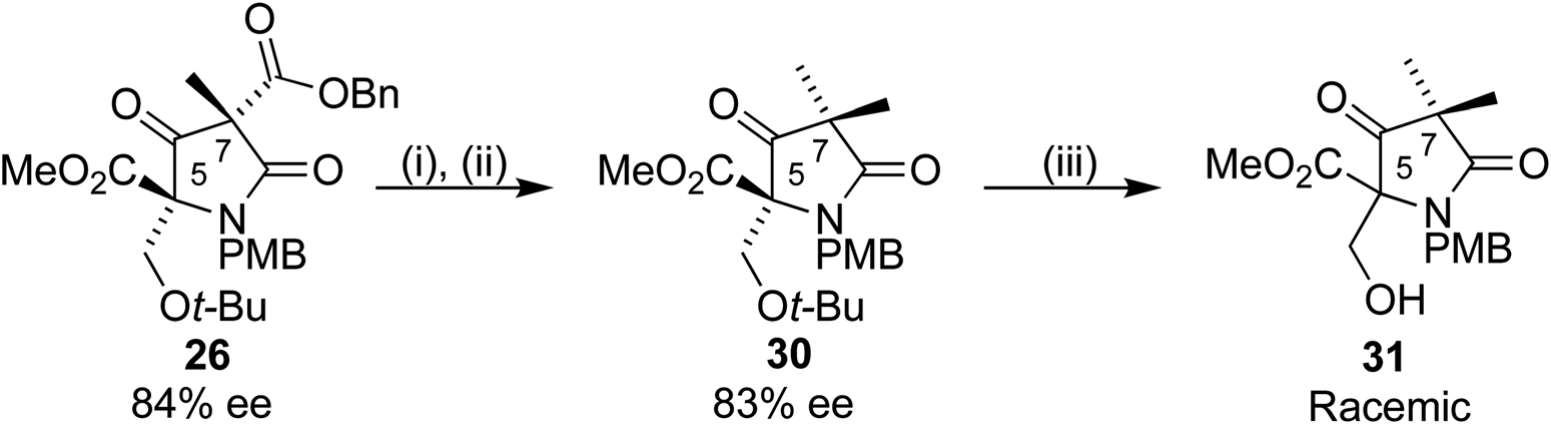
Reagents and conditions: (i) Pd(OH)_2_/C, H_2_, 35 °C, 16 h (ii) MeI, Et_3_N, DCM, 4 h (46% over 2 steps), (iii) TFA/DCM 1 : 1 67%.

A mechanism for this epimerization did not seem obvious, and a simpler substrate was synthesized to probe the reaction further. Use of methyl iodide as alkylating agent in place of 27 added a second methyl group to the C7 position, giving 30 (Scheme 10). This *gem*-methylated analogue of our substrate was chosen for two reasons, to eliminate a stereogenic centre, which could confirm C5 as the epimerizing centre, and to reduce the number of potentially reactive functional groups. Removal of the *t*-butyl group with TFA under the conditions used for 28a/b gave 31 in racemic form. Further analysis revealed that the stereocentre remained unchanged upon treatment of 30 with TFA. The racemization occurred during preparation for the purification by column chromatography, where crude 29a/b was dissolved in DCM and adsorbed onto silica gel, perhaps a result of a retroaldol process. We were pleased to find that changing to a wet loading method, where crude 31 was loaded onto the chromatography column in the eluting solvent using petroleum ether/ethyl acetate, resulted in complete preservation of the ee.

**Scheme 11:**
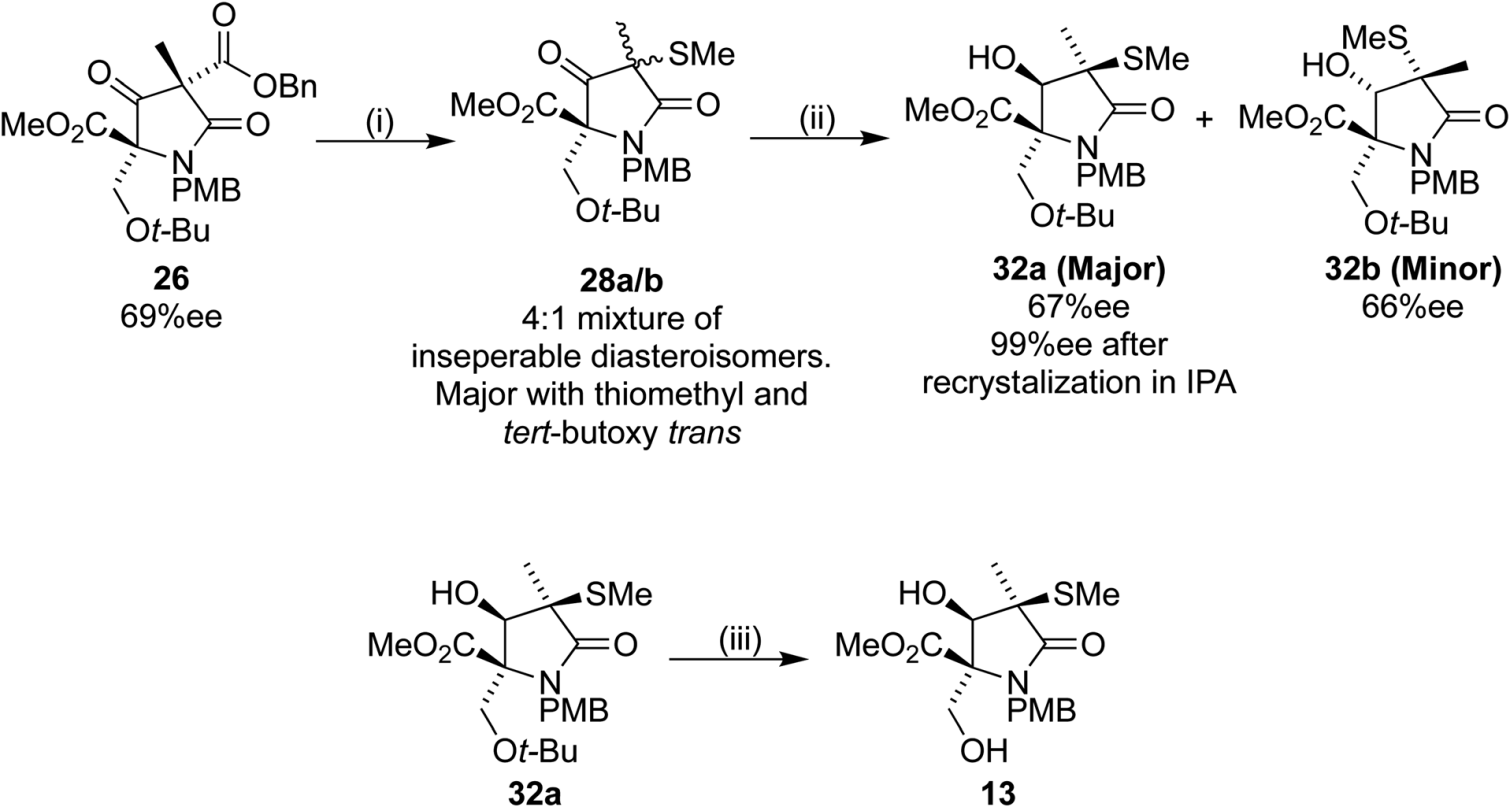
Reagents and conditions (i) (1) Pd(OH)_2_/C, H_2_, 35 °C, 16 h, (2) 27, Et_3_N, DCM, 4 h (70% over 2 steps); (ii) NaBH_4_, EtOH, 30 min, 0 °C: 32a, 54%, 32b, 13%; (iii) TFA/DCM 1 : 1, 75%.

Returning to our original system, the 4 : 1 mixture of 28a/b was treated with TFA/DCM to produce 29a/b. Analysis by ^1^H NMR spectroscopy showed retention of the 4 : 1 ratio in the crude mixture. Silica gel was added, and the NMR spectrum obtained again, now showing a ratio of 3 : 1.3. After leaving 29a/b in contact with the silica gel for 16 h, the ^1^H NMR spectroscopy was repeated, the spectrum this time showing a 3 : 2 ratio. Despite largely preventing the epimerization, we were unable to separate the diastereoisomers 29a/b efficiently. Because of this problem we changed the order of deprotection and reduction, aiming to produce the later Corey intermediate lactam 13 (Scheme 11).^18^

Sodium borohydride reduction of the inseparable 4 : 1 diastereoisomeric mixture 28a/b led to two separable isomers 32a and 32b in a 4 : 1 ratio. Analysis of the products by HPLC using a chiral stationary phase indicated ees of 67% and 66% for 32a and 32b respectively. The desired first eluting diastereoisomer 32a was recrystallized from IPA, to our delight providing 32a with 99% ee. Treatment of the filtrate with TFA/DCM resulted in the desired lactam 13 without further epimerization, thus completing our formal synthesis of (+)-lactacystin in six steps from 16.

A formal synthesis of (+)-lactacystin has thus been completed in six steps from 16. Lactam 13 has also been functionalized by Corey to give a variety of C9 analogues.^19^

## Experimental detail

### Benzyl (3*S*,5*S*)-3-methyl-5-(2-methylpropyl)-2,4-dioxopyrrolidine-3-carboxylate 20

Lactam 10a (1.2023 g, 2.83 mmol) was dissolved in a MeCN/water mixture (3 : 1, 27.6 mL). CAN (8.3 g, 14.22 mmol, 5 equiv.) was added and the solution stirred until complete consumption of the starting material had occurred (approx. 2 h). The solution was diluted with water (150 mL) and extracted with ethyl acetate (150 mL × 3). The organic layers were combined and washed with brine (100 mL × 2), dried (sodium sulfate), filtered, and the solvents removed under reduced pressure. The residue was purified by column chromatography using petroleum ether (40–60 °C)/ethyl acetate (8 : 2) as eluent to produce 20 as a colourless crystalline solid. (0.725 g, 84%). Mp 118–124 °C; [*α*]^24^_D_ = +11.6 (*c* 2.3, CHCl_3_) (9% ee); *ν*_max_ (thin film)/cm^−1^: 3209, 2960, 1781, 1748, 1705; ^1^H NMR (500 MHz, CDCl_3_) *δ*_H_ 7.38–7.30 (m, 3H), 7.29–7.23 (m, 2H), 7.02–6.60 (m, 1H), 5.24–511 (m, 2H), 4.18 (dd, *J* = 9.5, 3.5 Hz, 1H), 1.78–1.68 (m, 2H), 1.53 (s, 3H), 1.43 (m, 1H), 0.96 (d, *J* = 6.2 Hz, 3H), 0.94 (d, *J* = 6.2 Hz, 3H); ^13^C NMR (126 MHz, CDCl_3_) *δ*_C_ 206.7, 171.9, 165.5, 135.0, 128.8, 128.6, 127.9, 68.1, 61.4, 58.6, 41.3, 25.2, 23.2, 21.5, 15.9; HRMS (NSI-FTMS) *m*/*z* [M + NH_4_]^+^ calcd for [C_17_H_25_N_2_O_4_]^+^ 321.1809, found 321.1812.

### Sodium hydroxy(4-methoxyphenyl)methanesulfonate 18 (ref. 15)

*p*-Methoxybenzaldehyde (20 mL, 0.164 mol, 1.2 equiv.) was stirred in ethanol (330 mL). An aqueous solution of sodium sulfite (17.56 g, 0.139 mol in 34 mL) was added slowly, forming a cloudy precipitate. The mixture was stirred for 16 h at 30 °C, then stirred in an ice bath for 2 hours allowing further precipitation. The resulting suspension was filtered, and the collected solid washed with hexane and dried in a vacuum oven to yield the adduct sulphite 18 as a fluffy colourless solid (33.72 g, 81%). Mp 166–168 °C (decomp.) (lit^20^ 155–157 °C (decomp.)); *ν*_max_ (solid)/cm^−1^ 3226, 1516, 1249; ^1^H NMR (500 MHz, DMSO) *δ*_H_ 7.34 (d, *J* = 8.3 Hz, 2H), 6.79 (d, *J* = 8.8 Hz, 2H), 5.56 (d, *J* = 5.1 Hz, 1H), 4.87 (d, *J* = 5.0 Hz, 1H), 3.72 (s, 3H); ^13^C NMR (126 MHz, DMSO) *δ*_C_ 158.6, 131.6, 129.1, 112.6, 84.7, 55.2.

### (*S*)-Methyl 3-(*tert*-butoxy)-2-((4-methoxybenzyl)amino)propanoate 19

*t*-Bu*-O-*Serine methyl ester hydrochloride 17 (5 g, 23.61 mmol) was dissolved in methanol (250 mL). The sulfite salt 18 (9 g, 37.46 mmol, 1.6 equiv.) was added along with triethylamine (3 mL, 21.52 mmol 0.9 equiv.). The stirred solution was cooled with an ice bath, and sodium cyanoborohydride (9.09 g, 144 mmol, 6.1 equiv.) added in small portions. The suspension was kept overnight and allowed to reach room temperature, the solvents removed, and the residue dissolved in ethyl acetate (200 mL). The organic solution was washed with equal amounts of brine, saturated sodium sulfite solution, and water, dried (magnesium sulfate), filtered, and the solvents removed under reduced pressure to give 19 (5.92 g, 85%), which was used in the next step without further purification. For analytical purposes, a portion was purified by column chromatography on silica gel using petroleum ether (40–60 °C)/ethyl acetate (9 : 1) as eluent to yield 19 as a colourless oil. [*α*]^24^_D_ = −23.61 (*c* 1.05, CHCl_3_) *ν*_max_ (thin film)/cm^−1^: 3434, 2974, 1743; ^1^H NMR (500 MHz, CDCl_3_) *δ* 7.25 (d, *J* = 7.5 Hz, 2H), 6.85 (d, *J* = 8.7 Hz, 2H), 3.83 (d, *J* = 12.8 Hz, 1H), 3.79 (s, 3H), 3.72 (s, 3H), 3.65 (d, *J* = 12.8 Hz, 1H), 3.60 (dd, *J* = 8.7, 5.3 Hz, 1H), 3.55 (dd, *J* = 8.7, 4.9 Hz, 1H), 3.43 (t, *J* = 5.1 Hz, 1H), 2.14 (s, 1H), 1.14 (s, 9H); ^13^C NMR (126 MHz, CDCl_3_) *δ* 174.1, 158.8, 132.1, 129.7, 113.9, 73.3, 63.3, 61.1, 55.4, 51.8, 51.5, 27.5; HRMS (NSI-FTMS) *m*/*z* [M + H]^+^ calcd for [C_16_H_26_NO_4_]^+^ 296.1856, found 296.1859.

### (*S*)-Benzyl 3-((3-(*tert*-butoxy)-1-methoxy-1-oxopropan-2-yl)(4-methoxybenzyl)amino)-3-oxopropanoate 16

Crude amine 19 (5.92 g, 20.05 mmol) was dissolved in anhydrous DCM (182 mL) in a flame-dried flask. The benzyl malonic half ester 8 (11.59 g, 49.89 mmol, 2.5 equiv.) was added along with EDAC·HCl (10.30 g, 53.72 mmol 2.7 equiv.), DMAP (0.5 g, 4.1 mmol, 0.2 equiv.) and *N*-methyl morpholine (5.5 mL, 50 mmol, 2.5 equiv.). The solution was stirred for 16 h under an atmosphere of nitrogen. Aqueous HCl (1 M, 6 mL) was added, and the solution transferred into a separating funnel, and washed with water (200 mL) and brine (200 mL). The organic layer was dried (magnesium sulfate), filtered and the solvents removed under reduced pressure to give a yellow oil, which was purified by column chromatography on silica gel using petroleum ether (40–60 °C)/ethyl acetate (8 : 2) as eluent, to give 16 as a pale yellow oil. (5.493 g, 49% over the 2 steps) [*α*]^24^_D_ = −23.61 (*c* 1.05, CHCl_3_) *ν*_max_ (neat)/cm^−1^: 3434, 2974, 1743; ^1^H NMR (500 MHz, CDCl_3_) (major conformation) *δ*_H_ 7.40–7.29 (m, 5H), 7.24 (d, *J* = 8.8 Hz, 2H), 6.86 (d, *J* = 8.7 Hz, 2H), 5.15 (s, 2H), 4.69 (s, 2H), 4.58 (dd, *J* = 7.5, 3.4 Hz, 1H), 3.90 (dd, *J* = 9.7, 7.6 Hz, 1H), 3.80–3.74 (m, 4H), 3.67 (s, 3H), 3.51 (d, *J* = 15.4 Hz, 1H), 3.43 (d, *J* = 15.4 Hz, 1H), 1.06 (s, 9H); ^13^C NMR (126 MHz, CDCl_3_) *δ*_C_ 169.7, 167.3, 167.2, 159.1, 128.8, 128.6, 128.4, 128.1, 114.2, 73.4, 67.1, 60.7, 59.7, 55.4, 52.3, 52.1, 41.5, 27.3; HRMS (NSI-FTMS) *m*/*z* [M + H]^+^ calcd for [C_26_H_34_NO_7_]^+^ 472.2330, found 472.2325. Determination of ee (97% ee) was carried out by HPLC using an AD-H Chiralpak column 90 : 10 hexane/IPA, 230 nm, 0.8 mL min^−1^, 15 °C.

### (3*S*,5*S*)-Benzyl 5-(*tert*-butoxymethyl)-1-(4-methoxybenzyl)-3-methyl-2,4-dioxopyrrolidine-3-carboxylate 15a and (3*R*,5*S*)-benzyl 5-(*tert*-butoxymethyl)-1-(4-methoxybenzyl)-3-methyl-2,4-dioxopyrrolidine-3-carboxylate 15b

Compound 16 (1.605 g, 3.4 mmol) was dissolved in ether (9.8 mL), TBAF (1 M in THF) (7 mL, 7 mmol, 2.1 equiv.) was added, and the solution stirred for 10 m. The solvents were removed under reduced pressure, and the residue placed under high vacuum for approximately 2 h. To the resulting colourless solid was added THF (9.8 mL), and the solution cooled to −12 °C. MeI (2.1 mL, 34 mmol, 10 equiv.) was added and the solution stirred for 72 h. Water (approximately 3 mL) was added and the solution was allowed to reach room temperature. The solution was dissolved in ethyl acetate (100 mL), and washed with water (100 mL) and brine (100 mL). The organic layer was dried (magnesium sulfate), filtered, and evaporated. The resulting brown oil was purified by column chromatography on silica gel using petroleum ether (40–60 °C)/ethyl acetate (9 : 1) as the eluent, to produce 15a/b as a colourless oil in a 3 : 1 ratio of diastereoisomers (1.0273 g, 67%) which could be partially separated. For the major, first eluting diastereoisomer 15a: (0.491 g, 32%), [*α*]^19.5^_D_ = −91.3 (*c* 1.06, CHCl_3_) (98% ee) *ν*_max_ (neat)/cm^−1^: 2934, 1781, 1749, 1698; ^1^H NMR (400 MHz, CDCl_3_) *δ*_H_ 7.39–7.33 (m, 3H), 7.28–7.23 (m, 2H), 7.01 (d, *J* = 8.4 Hz, 2H), 6.60 (d, *J* = 8.7 Hz, 2H), 5.38 (d, *J* = 15.1 Hz, 1H), 5.21 (d, *J* = 12.3 Hz, 1H), 5.10 (d, *J* = 12.3 Hz, 1H), 3.86 (t, *J* = 2.3 Hz, 1H), 3.83 (d, *J* = 15.1 Hz, 1H), 3.74 (s, 3H), 3.58 (dd, *J* = 9.9, 2.1 Hz, 1H), 3.53 (dd, *J* = 9.9, 2.4 Hz, 1H), 1.56 (s, 3H), 1.12 (s, 9H); ^13^C NMR (126 MHz, CDCl_3_) *δ*_C_ 205.0, 170.3, 166.0, 159.3, 135.0, 129.3, 128.8, 128.6, 128.4, 126.7, 114.3, 73.9, 68.2, 65.2, 58.8, 57.2, 55.4, 43.1, 27.3, 15.3; HRMS (NSI-FTMS) *m*/*z* [M + H]^+^ calcd for [C_26_H_32_NO_6_]^+^ 254.2224, found 454.2218. Determined by HPLC to have 79% ee; recrystallization from IPA provided a supernatant of 98% ee. Determination of ee was carried out by HPLC using an AD-H Chiralpak column 80 : 20 hexane/IPA, 230 nm, 0.8 mL min^−1^, 25 °C. For the partially purified, minor, second eluting diastereomer 15b: (0.416 g, 35%), ^1^H NMR (400 MHz, CDCl_3_) *δ* 7.38–7.28 (m, 5H), 7.22 (d, *J* = 8.6 Hz, 2H), 6.82 (d, *J* = 8.7 Hz, 2H), 5.25–5.16 (m, 2H), 5.11 (d, *J* = 12.2 Hz, 1H), 4.31 (d, *J* = 14.3 Hz, 1H), 3.87 (dd, *J* = 7.2, 2.6 Hz, 1H), 3.78 (s, 3H), 3.59 (dd, *J* = 9.7, 2.5 Hz, 1H), 3.48 (dd, *J* = 9.7, 7.1 Hz, 1H), 1.52 (s, 3H), 1.02 (s, 9H). ^13^C NMR (101 MHz, CDCl_3_) *δ* 204.0, 169.6, 165.6, 159.4, 135.0, 130.0, 128.8, 128.7, 128.3, 128.0, 114.2, 74.0, 68.2, 65.5, 62.8, 58.5, 55.4, 44.5, 27.2, 16.7.

### (2*R*,4*S*)-4-Benzyl 2-methyl 2-(*tert*-butoxymethyl)-1-(4-methoxybenzyl)-4-methyl-3,5-dioxopyrrolidine-2,4-dicarboxylate 26

Lactam 15a (0.1125 g, 0.25 mmol), in a flame-dried flask, was dissolved in anhydrous THF (4.5 mL), cooled to −40 °C and placed under an atmosphere of nitrogen. LiHMDS (1 M in THF/ethylbenzene) (0.56 mL, 0.56 mmol, 2.2 equiv.) and DMPU (0.9 mL, 0.75 mmol, 3 equiv.) were added, and the solution allowed to stand for 0.5 h. Methyl cyanoformate (0.09 mL, 1.13 mmol, 4.5 equiv.) was added and stirring was continued for 3 h. Saturated aqueous NH_4_Cl (0.2 mL) was added, and the mixture allowed to reach room temperature. Water (20 mL) was added and the mixture extracted with equal amounts of ethyl acetate twice. The combined organic extracts were washed with water (20 mL) and brine (2 × 20 mL), dried (sodium sulfate), filtered, and the solvents removed under reduced pressure. The residue was purified by column chromatography using petroleum ether (40–60 °C)/ethyl acetate (9 : 1) as eluent to yield 26 as a colourless crystalline solid (0.0881 g, 69%). Mp 90–94 °C [*α*]^25^_D_ = +2.85 (*c* 1.12, CHCl_3_), *ν*_max_ (neat)/cm^−1^: 3434, 2974, 1743; ^1^H NMR (500 MHz, CDCl_3_) *δ*_H_ 7.39–7.30 (m, 5H), 7.18 (d, *J* = 8.7 Hz, 2H), 6.70 (d, *J* = 8.7 Hz, 2H), 5.24 (d, *J* = 12.5 Hz, 1H), 5.16 (d, *J* = 12.5 Hz, 1H), 4.72 (d, *J* = 15.3 Hz, 1H), 4.57 (d, *J* = 15.3 Hz, 1H), 3.83 (d, *J* = 9.7 Hz, 1H), 3.75 (s, 3H), 3.73 (d, *J* = 9.7 Hz, 1H), 3.53 (s, 3H), 1.66 (s, 3H), 0.87 (s, 9H); ^13^C NMR (126 MHz, CDCl_3_) *δ*_C_ 199.7, 170.7, 166.3, 165.1, 159.0, 135.1, 129.5, 128.7, 128.6, 128.5, 128.0, 113.7, 74.1, 68.1, 61.0, 57.7, 55.5, 53.4, 44.7, 26.8, 18.9; HRMS (NSI-FTMS) *m*/*z* [M + H]^+^ calcd for [C_28_H_34_NO_8_]^+^ 512.2279, found 512.2271. Determination of ee was carried out by HPLC using an AD-H Chiralpak column 90 : 10 hexane/IPA, 230 nm, 0.8 mL min^−1^, 25 °C.

### Methyl (2*S*)-2-anilino-3-*tert*-butoxypropanoate 33 (ref. 21)

Compound 17 (1.249 g, 5.9 mmol) was dissolved in anhydrous DCM (40 mL). Phenyl boronic acid (1.447 g, 11.94 mmol, 2 equiv.) was added, together with Cu(OAc)_2_ (1.2 g, 6.6 mmol, 1.1 equiv.). Triethylamine (1.65 mL, 11.8 mmol, 2 equiv.) and molecular sieve (4 Å, 4.4 g) were added, and the mixture was placed under a static atmosphere of oxygen and stirred for 3 days. Aqueous ammonium hydroxide (1 M, 40 mL) was added, and the suspension stirred for 40 min and filtered through diatomaceous earth. The organic layer was removed, and the aqueous layer extracted with DCM (50 mL). The organic layers were combined, washed with water (80 mL) and brine (80 mL), dried (magnesium sulfate), filtered, and the solvents removed under reduced pressure. The residue was purified by column chromatography using petroleum ether (40–60 °C)/ether 18 : 1 as eluent to yield 33 as a cream coloured solid (0.319 g, 22%). Recrystallization from petroleum ether gave crystals of 95% ee. Mp 52–59 °C (lit^21^ 47–50 °C); [*α*]^22^_D_ = −13.69 (*c* 1.11, CHCl_3_) (95% ee) [*α*]^24^_D_ = (lit^21^ [*α*]^24^_D_ = −10.7 (*c* 1.1, CHCl_3_) (71% ee)); *ν*_max_ (neat)/cm^−1^: 3398, 2975, 1751; ^1^H NMR (500 MHz, CDCl_3_) *δ*_H_ 7.20–7.14 (m, 2H), 7.75 (td, *J* = 7.4, 0.8 Hz, 1H), 6.64 (d, *J* = 8.2 Hz, 2H), 4.62 (s, 1H), 4.20 (t, *J* = 4.1 Hz, 1H), 3.78 (dd, *J* = 8.8, 4.0 Hz, 1H), 3.73 (s, 3H), 3.69 (dd, *J* = 8.8, 4.2 Hz, 1H), 1.17 (s, 9H); ^13^C NMR (126 MHz, CDCl_3_) *δ*_C_ 172.0, 146.9, 129.40, 118.5, 113.8, 73.70, 62.60, 57.4, 52.3, 27.5. Determination of ee was carried out by HPLC using a Eurocel 01 Knauer column 90 : 10 hexane/IPA, 230 nm, 0.8 mL min^−1^, 25 °C.

### (*S*)-Benzyl 3-((3-(*tert*-butoxy)-1-methoxy-1-oxopropan-2-yl)(phenyl)amino)-3-oxopropanoate 24

Amine 33 (0.2308 g, 0.91 mmol) was dissolved in anhydrous DCM (7 mL) in a flame-dried flask. The benzyl malonic half ester 8 (0.47 g, 2.02 mmol, 2.2 equiv.) was added together with EDAC·HCl (0.467 g, 2.4 mmol 2.7 equiv.), DMAP (0.021 g, 0.17 mmol, 0.19 equiv.) and *N*-methylmorpholine (0.23 mL, 2.09 mmol, 2.3 equiv.). The mixture was stirred for 16 h under a nitrogen atmosphere. Aqueous HCl (0.3 mL, 1 M solution) was added, and the solution diluted with DCM (50 mL). The solution was washed with water (50 mL) and brine (50 mL). The organic layer was dried (magnesium sulfate), filtered, and the solvents removed under reduced pressure to give a yellow oil, which was purified by column chromatography using petroleum ether (40–60 °C)/ethyl acetate (9 : 1 to 7 : 3) as eluent to yield 24 as colourless oil (0.1136 g, 71%). [*α*]^23^_D_ = +1.48 (*c* 0.54, CHCl_3_) (95% ee) *ν*_max_ (neat)/cm^−1^: 3022, 2974, 1743, 1663; ^1^H NMR (500 MHz, CDCl_3_) *δ*_H_ 7.45 (s, 2H), 7.38–7.28 (m, 8H), 5.10 (s, 2H), 4.69–4.64 (m, 1H), 3.81–3.78 (m, 2H), 3.75 (s, 3H), 3.24 (d, *J* = 15.7 Hz, 1H), 3.20 (d, *J* = 15.8 Hz, 1H), 1.08 (s, 9H). ^13^C NMR (126 MHz, CDCl_3_) *δ*_C_ 169.8, 167.3, 166.4, 141.5, 135.6, 129.6, 129.5, 128.8, 128.6, 128.5, 128.4, 73.5, 67.1, 62.0, 59.2, 52.4, 42.2, 27.4; HRMS (NSI-FTMS) *m*/*z* [M + H]^+^ calcd for [C_24_H_30_NO_6_]^+^ 428.2068, found 428.2065. Determination of ee was carried out by HPLC using an AD-H Chiralpak column 80 : 20 hexane/IPA, 230 nm, 0.8 mL min^−1^, 25 °C.

### (3*S*,5*S*)-Benzyl 5-(*tert*-butoxymethyl)-3-methyl-2,4-dioxo-1-phenylpyrrolidine-3-carboxylate 25

The Dieckmann cyclization precursor 24 (0.104 g, 0.24 mmol) was dissolved in ether (0.67 mL), TBAF (1 M in THF), (0.5 mL, 0.5 mmol, 2.1 equiv.) added, and the solution stirred for 10 min. The solvents were removed under reduced pressure, and the residue placed under high vacuum for approximately 2 h. THF (0.67 mL) was added, and the solution cooled to −12 °C. MeI (0.07 mL, 1.12 mmol, 4.7 equiv.) was added, and the suspension stirred for 72 h. Water (0.1 mL) was added, the solution allowed to reach room temperature, ethyl acetate (30 mL) added, and the solution washed with water (30 mL) and brine (30 mL). The organic layer was dried (magnesium sulfate), filtered, and the solvents removed under reduced pressure. The resulting brown oil was purified by column chromatography using petroleum ether (40–60 °C)/ethyl acetate (9 : 1) as the eluent. Only the major diastereoisomer 25 could be isolated (0.0081 g, 8%) mp 121–124 °C [*α*]^22^_D_ = +14.2 (*c* 0.81, CHCl_3_) (87% ee) *ν*_max_ (neat)/cm^−1^: 2977, 2253, 1782, 1752, 1702; ^1^H NMR (500 MHz, CDCl_3_) *δ*_H_ 7.44–7.23 (m, 10H), 5.18 (s, 2H), 4.57 (t, *J* = 2.1 Hz, 1H), 3.68 (dd, *J* = 9.6, 1.8 Hz, 1H), 3.39 (dd, *J* = 9.6, 2.4 Hz, 1H), 1.65 (s, 3H), 1.02 (s, 9H); ^13^C NMR (126 MHz, CDCl_3_) *δ*_C_ 204.5, 169.5, 165.8, 135.8, 135.2, 129.4, 128.8, 128.5, 127.8, 127.5, 125.6, 73.9, 68.8, 68.0, 59.7, 57.9, 27.2, 15.2; HRMS (NSI-FTMS) *m*/*z* [M + H]^+^ calcd for [C_24_H_28_NO_5_S]^+^ 410.1962, found 410.1956. Determination of ee (87% ee) was carried out by HPLC using an AD-H Chiralpak column 80 : 20 hexane/IPA, 230 nm, 0.8 mL min^−1^, 25 °C.

### Potassium 3-methoxy-3-oxopropanoate 34 (ref. 22)

KOH (22.06 g, 393.18 mmol, 1.2 equiv.) was dissolved in methanol (75 mL). The resulting solution was added to a solution of dimethyl malonate (38 mL, 332.5 mmol) in methanol (85 mL). A colourless precipitate was formed, which was collected by vacuum filtration and dried in a vacuum oven to provide (26.35 g, 51%) of 34 as a colourless solid. Mp: 204–209 °C (lit^23^ 204–207 °C); *ν*_max_ (solid)/cm^−1^: 1726, 1595, 1368; ^1^H NMR (500 MHz, D_2_O) *δ*_H_ 3.75 (s, 3H), 3.34 (s, 2H); 13C NMR (101 MHz, D_2_O) *δ*_C_ 174.8, 172.6, 53.2, 45.0.

### (*S*)-Methyl 3-(*tert*-butoxy)-2-(3-methoxy-*N*-(4-methoxybenzyl)-3-oxopropanamido)propanoate 21

Amine 19 (0.5558 g, 1.88 mmol) was dissolved in anhydrous DCM (17.5 mL) in a flame-dried flask. The methyl malonic half ester 34 (0.692 g, 4.43 mmol, 2.4 equiv.) was added together with EDAC·HCl (1.06 g, 5.53 mmol 2.9 equiv.), DMAP (0.0405 g, 0.33 mmol, 0.18 equiv.) and *N*-methylmorpholine (0.5 mL, 4.54 mmol, 2.4 equiv.). The mixture was stirred for 24 h under an atmosphere of nitrogen. Aqueous HCl (1 M, 0.5 mL) was added, and the solution washed with water (20 mL) and brine (20 mL). The organic layer was dried (magnesium sulfate), filtered, and the solvents removed under reduced pressure to give a yellow oil, which was purified by column chromatography using petroleum ether (40–60 °C)/ethyl acetate (8 : 2) as eluent to yield the desired compound 21 as a pale-yellow oil (0.54 g, 73%). [*α*]^26^_D_ = −42.0 (*c* 1.13, CHCl_3_); *ν*_max_ (neat)/cm^−1^: 2973, 1744, 1656, 1514; ^1^H NMR (400 MHz, CDCl_3_) *δ*_H_ 7.29 (d, *J* = 7.5 Hz, 2H), 6.91 (d, *J* = 8.7 Hz, 2H), 4.73 (s, 2H), 4.60 (dd, *J* = 7.6, 3.4 Hz, 1H), 3.93 (dd, *J* = 9.7, 7.6 Hz, 1H), 3.86–3.81 (m, 4H), 3.74 (s, 3H), 3.72 (s, 3H), 3.50 (d, *J* = 15.3 Hz, 1H), 3.40 (d, *J* = 15.3 Hz, 1H), 1.10 (s, 9H); ^13^C NMR (101 MHz, CDCl_3_) *δ*_C_ 169.7, 167.8, 167.4, 159.2, 128.8, 128.1, 114.2, 73.5, 60.7, 59.8, 55.4, 52.5, 52.3, 52.2, 41.3, 27.3; HRMS (NSI-FTMS) *m*/*z* [M + H]^+^ calcd for [C_20_H_30_NO_7_]^+^ 396.2017, found 396.2016.

### (3*S*,5*S*)-Methyl 5-(*tert*-butoxymethyl)-1-(4-methoxybenzyl)-3-methyl-2,4-dioxopyrrolidine-3-carboxylate 22a and (3*R*,5*S*)-methyl 5-(*tert*-butoxymethyl)-1-(4-methoxybenzyl)-3-methyl-2,4-dioxopyrrolidine-3-carboxylate 22b

The Dieckmann cyclization precursor 21 (0.2219 g, 0.56 mmol) was dissolved in ether (1.6 mL). TBAF (1 M in THF), (1.6 mL, 1.6 mmol, 3 equiv.) was added, and the solution stirred for 5 min. The solvents were removed under reduced pressure, and the resulting brown oil was dissolved in THF (1.6 mL). The solution was cooled to −12 °C, MeI (0.15 mL, 2.4 mmol, 4.3 equiv.) added, and the suspension stirred for 58 h. Water (1 mL) was added, and the mixture added to a short silica gel column. The column was eluted with ethyl acetate. The 3 : 1 mixture of diastereoisomers 22a/b was collected as a yellow oil and used onto the next step without further purification (0.1832 g, 87%). Data for the major diastereoisomer 22a: ^1^H NMR (400 MHz, CDCl_3_) *δ*_H_ 7.21 (d, *J* = 8.5 Hz, 2H), 6.87 (d, *J* = 8.6 Hz, 2H), 5.35 (d, *J* = 15.0 Hz, 1H), 3.93 (d, *J* = 15.0 Hz, 1H), 3.93 (t, *J* = 2.2 Hz, 1H), 3.80 (s, 3H), 3.73 (s, 3H), 3.60 (dd, *J* = 9.8, 2.2 Hz, 1H), 3.56 (dd, *J* = 9.9, 2.4 Hz, 1H), 1.53 (s, 3H), 1.11 (s, 9H). ^13^C NMR (101 MHz, CDCl_3_) *δ*_C_ 205.0, 170.5, 166.7, 159.5, 129.5, 127.2, 114.4, 73.9, 65.4, 58.7, 57.4, 55.5, 53.4, 43.4, 27.3, 15.2. Data for the minor diastereoisomer 22b: ^1^H NMR (400 MHz, CDCl_3_) *δ*_H_ 7.23 (d, *J* = 6.6 Hz, 2H), 6.85 (d, *J* = 7.2 Hz, 2H), 5.27 (d, *J* = 14.6 Hz, 1H), 4.23 (d, *J* = 14.5 Hz, 1H), 3.86 (dd, *J* = 5.8, 2.7 Hz, 1H), 3.79 (s, 3H), 3.75 (s, 3H), 3.69 (m, 2H), 1.52 (s, 3H), 1.17 (s, 9H). ^13^C NMR (101 MHz, CDCl_3_) *δ*_C_ 204.0, 169.0, 166.3, 159.5, 129.0, 127.8, 114.3, 74.0, 65.1, 61.3, 58.3, 55.4, 53.4, 44.2, 27.4, 17.4. Data for both diastereoisomers: *ν*_max_ (neat)/cm^−1^: 2974, 1782, 1750, 1698, 1514; HRMS (NSI-FTMS) *m*/*z* [M + H]^+^ calcd for [C_20_H_28_NO_6_]^+^ 378.1911, found 378.1913.

### (3*S*,5*S*)-Methyl 5-(*tert*-butoxymethyl)-3-methyl-2,4-dioxopyrrolidine-3-carboxylate 23a and (3*R*,5*S*)-methyl 5-(*tert*-butoxymethyl)-3-methyl-2,4-dioxopyrrolidine-3-carboxylate 23b

The mixture of diastereoisomers 22a/b (0.1251 g, 0.331 mmol) was dissolved in a MeCN/H_2_O mixture (3 : 1) (3.5 mL). CAN (0.9 g, 1.64 mmol, 5 equiv.) was added, and the solution stirred vigorously until consumption of the starting material had occurred (approx. 1.5 h). Ethyl acetate (25 mL) was added, and the solution washed with water (25 mL). The aqueous layer was extracted with ethyl acetate (25 mL). The combined organic layers were washed with water (25 mL) and brine (25 mL), dried (magnesium sulfate), filtered, and the solvents removed under reduced pressure. The crude material was purified by column chromatography using petroleum ether (40–60 °C)/ethyl acetate (8 : 2–7 : 3) as the eluent to give a mixture of partially separable diastereoisomers (0.0483 g, 57% total) from which only 23a could be obtained uncontaminated (0.01 g, 12%). Data for the major diastereoisomer 23a: mp 111–116 °C; [*α*]^24^_D_ = −46.2 (*c* 0.91, CHCl_3_); *ν*_max_ (neat)/cm^−1^: 3234, 2976, 1785, 1750, 1706; ^1^H NMR (400 MHz, CDCl_3_) *δ*_H_ 6.72 (d, *J* = 39.7 Hz, 1H), 4.18 (dd, *J* = 9.2, 3.6 Hz, 1H), 3.77–3.69 (m, 4H), 3.53 (t, *J* = 9.1 Hz, 1H), 1.55 (s, 3H), 1.19 (s, 9H); ^13^C NMR (101 MHz, CDCl_3_) *δ*_C_ 204.4, 171.4, 166.2, 74.3, 63.4, 62.4, 58.5, 53.6, 27.5, 16.7; HRMS (NSI-FTMS) *m*/*z* [M + H]^+^ calcd for [C_12_H_20_NO_5_]^+^ 258.1336, found 258.1339. Data for the minor diastereoisomer 23b: ^1^H NMR (400 MHz, CDCl_3_) *δ*_H_ 6.73–6.31 (m, 1H), 4.31 (t, *J* = 3.6 Hz, 1H), 3.74 (s, 3H), 3.61 (d, *J* = 3.6 Hz, 2H), 1.51 (s, 3H), 1.14 (s, 9H); ^13^C NMR (101 MHz, CDCl_3_) *δ*_C_ 205.2, 172.2, 166.3, 74.1, 63.5, 61.0, 58.6, 53.6, 27.4, 15.1.

### (2*R*,4*R*)-Methyl 2-(*tert*-butoxymethyl)-1-(4-methoxybenzyl)-4-methyl-4-(methylthio)-3,5-dioxopyrrolidine-2-carboxylate 28a and (2*R*,4*S*)-methyl 2-(*tert*-butoxymethyl)-1-(4-methoxybenzyl)-4-methyl-4-(methylthio)-3,5-dioxopyrrolidine-2-carboxylate 28b

Methyl ester 26 (0.1426 g, 0.31 mmol) was dissolved in THF (0.7 mL), and the solution heated to 35 °C. A micro-spatula of Pd(OH)_2_/C was added and the stirred solution placed under a static atmosphere of hydrogen. After 16 h the solution was filtered through diatomaceous earth and washed with DCM. The solvents were removed under reduced pressure to produce a foam, which was dissolved in anhydrous DCM (0.5 mL) in a flame-dried flask. 27 (0.1090 g, 0.53 mmol, 1.7 equiv.) was added together with triethylamine (0.05 mL, 0.35 mmol, 1.1 equiv.). The solution was stirred at 20 °C for 5 h under an atmosphere of nitrogen, and the solvents removed under reduced pressure to give a yellow oil, which was purified by column chromatography using petroleum ether (40–60 °C)/ethyl acetate (9 : 1) as the eluent to yield a 4 : 1 mixture of inseparable diastereoisomers 28a/b as a pale-yellow oil (0.127 g, 70%). Data for the major diastereoisomer 28a: ^1^H NMR (500 MHz, CDCl_3_) *δ*_H_ 7.26 (d, *J* = 8.7 Hz, 2H), 6.82 (d, *J* = 8.7 Hz, 2H), 4.74 (d, *J* = 15.2 Hz, 1H), 4.44 (d, *J* = 15.2 Hz, 1H), 3.89 (d, *J* = 9.6 Hz, 1H), 3.78 (s, 3H), 3.72 (d, *J* = 9.6 Hz, 1H), 3.47 (s, 3H), 2.16 (s, 3H), 1.57 (s, 3H), 1.01 (s, 9H); ^13^C NMR (101 MHz, CDCl_3_) *δ* 201.6, 172.5, 166.2, 159.3, 130.3, 128.4, 113.8, 76.2, 74.3, 60.3, 55.4, 52.0, 49.7, 44.1, 27.1, 18.0, 12.6. Data for the minor diastereoisomer 28b: ^1^H NMR (500 MHz, CDCl_3_) *δ*_C_ 7.26 (d, *J* = 8.7 Hz, 2H), 6.82 (d, *J* = 8.7 Hz, 2H), 4.84 (d, *J* = 15.1 Hz, 1H), 4.36 (d, *J* = 15.1 Hz, 1H), 4.00 (d, *J* = 10.2 Hz, 1H), 3.86 (d, *J* = 10.2 Hz, 1H), 3.78 (s, 3H), 3.37 (s, 3H), 2.20 (s, 3H), 1.61 (s, 3H), 1.08 (s, 9H); ^13^C NMR (101 MHz, CDCl_3_) *δ* 203.4, 172.7, 166.7, 159.3, 130.4, 128.0, 113.8, 75.8, 74.4, 59.7, 55.4, 53.1, 50.7, 44.2, 27.1, 20.7, 12.9. Data for both diastereoisomers: *ν*_max_ (neat)/cm^−1^: 3020, 1741, 1699; HRMS (NSI-FTMS) *m*/*z* [M + H]^+^ calcd for [C_21_H_30_NO_6_S]^+^ 424.1788, found 424.1790.

### (2*R*,4*R*)-Methyl 2-(hydroxymethyl)-1-(4-methoxybenzyl)-4-methyl-4-(methylthio)-3,5-dioxopyrrolidine-2-carboxylate 29a (ref. 17) and (2*R*,4*S*)-methyl 2-(hydroxymethyl)-1-(4-methoxybenzyl)-4-methyl-4-(methylthio)-3,5-dioxopyrrolidine-2-carboxylate 29b

The 4 : 1 mixture of inseparable diastereoisomers 28a/b (0.0297 g, 0.07 mmol) was dissolved in anhydrous DCM (0.15 mL) in a flame-dried flask, and TFA (0.15 mL) added. The solution was stirred under nitrogen for 1.5 h until TLC showed complete consumption of starting material. The solution was diluted with DCM (50 mL), and washed with water (50 mL). The organic layer was washed with aqueous NaHCO_3_ (50 mL), dried (sodium sulfate), filtered, and the solvents removed under reduced pressure to give a brown residue, which was purified by column chromatography using petroleum ether (40–60 °C)/ethyl acetate (2 : 1) as eluent to yield 29a/b as a 2 : 1 mixture of inseparable isomers (0.0195 g, 76%). Data for the major diastereomer 29a: ^1^H NMR (400 MHz, CDCl_3_) *δ*_H_ 7.33 (d, *J* = 8.6 Hz, 2H), 6.86 (d, *J* = 8.6 Hz, 2H), 5.08 (d, *J* = 15.2 Hz, 1H), 4.35 (d, *J* = 15.2 Hz, 1H), 4.17 (d, *J* = 12.0 Hz, 1H), 3.79 (d, *J* = 12.8 Hz, 4H), 3.67 (s, 3H), 2.12 (s, 3H), 1.54 (s, 3H); ^13^C NMR (101 MHz, CDCl_3_) *δ*_C_ 199.1, 172.2, 165.7, 159.7, 129.9, 128.8, 114.6, 77.6, 61.8, 55.4, 53.4, 49.7, 44.3, 16.9, 12.4. Analysis by HPLC determined the ee to be 58% ee. Data for the minor diastereomer 29b: ^1^H NMR (400 MHz, CDCl_3_) *δ*_H_ 7.27 (d, *J* = 9.7 Hz, 2H), 6.84 (d, *J* = 7.2 Hz, 2H), 4.68 (d, *J* = 15.0 Hz, 1H), 4.60 (d, *J* = 15.0 Hz, 1H), 4.20 (d, *J* = 11.8 Hz, 1H), 4.07 (d, *J* = 12.6 Hz, 1H), 3.77 (s, 3H), 3.42 (s, 3H), 2.17 (s, 3H), 1.61 (s, 3H); ^13^C NMR (101 MHz, CDCl_3_) *δ*_C_ 200.3, 172.2, 166.7, 159.6, 130.3, 127.9, 114.3, 76.6, 62.01, 55.4, 53.4, 49.5, 44.4, 18.1, 12.2. Analysis by HPLC determined the ee to be 41% ee. Data for both diastereoisomers: *ν*_max_ (neat)/cm^−1^: 3419, 3000, 2932, 1776, 1742, 1699; HRMS (NSI-FTMS) *m*/*z* [M + H]^+^ calcd for [C_17_H_22_NO_6_S]^+^ 368.1162, found 368.1165. Determination of ee was carried out by HPLC using an AD-H Chiralpak column, 80 : 20 hexane/IPA, 230 nm, 0.8 mL min^−1^, 25 °C.

### (*R*)-Methyl 2-(*tert*-butoxymethyl)-1-(4-methoxybenzyl)-4,4-dimethyl-3,5-dioxopyrrolidine-2-carboxylate 30

Methyl ester 26 (0.0832 g, 0.16 mmol) was dissolved in THF (0.5 mL) and the solution heated to 35 °C. Pd(OH)_2_/C (20% nominally) (0.604 g) was added and the solution placed under a static atmosphere of hydrogen. After 16 h the solution was filtered through diatomaceous earth and washed through with DCM. The solvents were removed under reduced pressure to produce a foam which was dissolved in DCM (0.6 mL). The solution was stirred under an atmosphere of nitrogen and triethylamine (0.04 mL, 0.29 mmol, 1.8 equiv.) added. After 15 min, MeI was added (0.04 mL 0.64 mmol, 4 equiv.), and the solution stirred for a further 5 h, diluted with DCM (50 mL), and washed with water (50 mL) and brine (50 mL). The organic layer was dried (sodium sulfate), filtered, the solvents were removed under reduced pressure, and the residue purified by column chromatography using petroleum ether (40–60 °C)/ethyl acetate (8 : 2) as eluent to produce a pink oil. The oil was dissolved in DCM and washed with a saturated sodium thiosufate solution until the organic layer became colourless, dried (sodium sulfate), filtered, and the solvents removed under reduced pressure to produce 30 as a colourless oil (0.0286 g, 46% over the 2 steps). [*α*]^22^_D_ = +50 (*c* 0.31, CHCl_3_); *ν*_max_ (neat)/cm^−1^: 3019, 2978, 1779, 1742; ^1^H NMR (500 MHz, CDCl_3_) *δ*_H_ 7.22 (d, *J* = 8.7 Hz, 2H), 6.81 (d, *J* = 8.7 Hz, 2H), 4.87 (d, *J* = 15.1 Hz, 1H), 4.19 (d, *J* = 15.1 Hz, 1H), 3.89 (d, *J* = 9.8 Hz, 1H), 3.77 (s, 3H), 3.75 (d, *J* = 9.8 Hz, 1H), 3.31 (s, 3H), 1.34 (s, 3H), 1.28 (s, 3H), 1.05 (s, 9H); ^13^C NMR (126 MHz, CDCl_3_) *δ*_C_ 209.1, 177.3, 166.9, 159.3, 130.5, 128.2, 113.8, 75.8, 74.1, 58.9, 55.4, 52.8, 46.1, 43.3, 27.2, 22.2, 20.6; HRMS (NSI-FTMS) *m*/*z* [M + H]^+^ calcd for [C_21_H_30_NO_6_]^+^ 392.2068, found 392.2068. Determination of ee was carried out by HPLC using an Eurocel 01 Knauer column, 95 : 5 hexane/IPA, 230 nm, 0.8 mL min^−1^, 25 °C.

### (*R*)-Methyl 2-(hydroxymethyl)-1-(4-methoxybenzyl)-4,4-dimethyl-3,5-dioxopyrrolidine-2-carboxylate 31

Lactam 30 (0.0109 g, 0.027 mmol) was dissolved in dry DCM (0.1 mL), and TFA (0.1 mL) added. The solution was stirred under nitrogen for 1.5 h until TLC showed complete consumption of starting material. The solution was diluted to 50 mL with DCM and washed with water (50 mL). The organic layer was washed with a saturated NaHCO_3_ solution (50 mL), dried (sodium sulfate), filtered, and the solvents removed under reduced pressure to give a colourless solid, which was purified by column chromatography using petroleum ether (40–60 °C)/ethyl acetate (2 : 1) as eluent to yield the deprotected alcohol 31 as a gum (0.0062 g, 67%). [*α*]^24^_D_ = −7.74 (*c* 0.62, CHCl_3_) (86% ee) *ν*_max_ (neat)/cm^−1^: 3396, 2919, 1778, 1742, 1678; ^1^H NMR (500 MHz, CDCl_3_) *δ*_H_ 7.30 (d, *J* = 8.7 Hz, 2H), 6.86 (d, *J* = 8.7 Hz, 2H), 4.89 (d, *J* = 15.1 Hz, 1H), 4.31 (d, *J* = 15.1 Hz, 1H), 4.14 (dd, *J* = 12.3, 8.6 Hz, 1H), 3.85 (dd, *J* = 12.3, 4.4 Hz, 1H), 3.79 (s, 3H), 3.55 (s, 3H), 1.35 (s, 3H), 1.28 (s, 3H), 1.17 (dd, *J* = 8.6, 4.5 Hz, 1H); ^13^C NMR (126 MHz, CDCl_3_) *δ*_C_ 208.3, 177.3, 166.4, 159.6, 130.1, 128.7, 114.5, 77.3, 60.8, 55.4, 53.2, 46.1, 43.7, 22.0, 20.5; HRMS (NSI-FTMS) *m*/*z* [M + H]^+^ calcd for [C_17_H_22_NO_6_]^+^ 336.1442, found 336.1443. Determination of ee (86% ee) was carried out by HPLC using an AD-H Chiralpak column, 80 : 20 hexane/IPA, 230 nm, 0.8 mL min^−1^, 25 °C.

### (2*R*,3*R*,4*R*)-Methyl 2-(*tert*-butoxymethyl)-3-hydroxy-1-(4-methoxybenzyl)-4-methyl-4-(methylthio)-5-oxopyrrolidine-2-carboxylate 32a and (2*R*,3*S*,4*S*)-methyl 2-(*tert*-butoxymethyl)-3-hydroxy-1-(4-methoxybenzyl)-4-methyl-4-(methylthio)-5-oxopyrrolidine-2-carboxylate 32b

The mixture of diastereoisomers 28a/b (0.1407 g, 0.33 mmol) was dissolved in ethanol (9 mL) and the solution cooled with an ice bath. Sodium borohydride (0.0073 g, 0.19 mmol, 0.6 equiv.) was added, and the mixture stirred for 20 min. Water (40 mL) was added, and the resulting solution extracted with ethyl acetate (3 × 40 mL). The combined organic extracts were washed with brine (120 mL), dried (sodium sulfate), filtered, and the solvents removed under reduced pressure. The residue was purified using column chromatography using petroleum ether (40–60 °C)/ethyl acetate (8 : 2–2 : 1) as eluent to provide two separable diastereoisomers as gums, 32a, the first eluting diastereoisomer (0.0764 g, 54%) and 32b, the second (0.0181 g, 13%). Analysis by chiral HPLC showed the ees of the diastereoisomers to be 67% and 66% ee respectively. Diastereomer 32a upon recrystallization from IPA gave 0.0511 g of material from the supernatant at 99% ee. Data for major, first eluting diastereoisomer 32a: [*α*]^23^_D_ = +6.2 (*c* 0.71, CHCl_3_) (99% ee) *ν*_max_ (neat)/cm^−1^: 3418, 2973, 2926, 1743, 1697; ^1^H NMR (500 MHz, CDCl_3_) *δ*_H_ 7.24 (d, *J* = 8.7 Hz, 2H), 6.81 (d, *J* = 8.7 Hz, 2H), 4.71 (d, *J* = 15.2 Hz, 1H), 4.46 (d, *J* = 15.2 Hz, 1H), 3.99–3.87 (m, 2H), 3.82 (d, *J* = 9.7 Hz, 1H), 3.78 (s, 3H), 3.65 (s, 3H), 3.44 (d, *J* = 9.7 Hz, 1H), 2.12 (s, 3H), 1.60 (s, 3H), 1.04 (s, 9H); ^13^C NMR (126 MHz, CDCl_3_) *δ*_C_ 173.0, 172.1, 158.9, 130.4, 129.6, 113.7, 77.9, 74.1, 70.7, 62.8, 55.4, 53.4, 52.5, 45.2, 27.2, 22.9, 12.3; HRMS (NSI-FTMS) *m*/*z* [M + H]^+^ calcd for [C_21_H_32_NO_6_S]^+^ 426.1945, found 426.1942. Data for the minor, second eluting diastereomer 32b: [*α*]^22^_D_ = +5.26 (*c* 0.38, CHCl_3_) (66% ee) *ν*_max_ (neat)/cm^−1^: 3385, 3016, 2975, 1743, 1686; ^1^H NMR (500 MHz, CDCl_3_) *δ* 7.18 (d, *J* = 8.6 Hz, 2H), 6.81 (d, *J* = 8.7 Hz, 2H), 4.77 (d, *J* = 15.5 Hz, 1H), 4.43 (d, *J* = 15.5 Hz, 1H), 4.32 (s, 1H), 3.96 (d, *J* = 9.6 Hz, 1H), 3.77 (s, 3H), 3.75 (s, 1H), 3.67 (d, *J* = 9.7 Hz, 1H), 3.64 (s, 3H), 2.19 (s, 3H), 1.53 (s, 3H), 1.02 (s, 9H); ^13^C NMR (126 MHz, CDCl_3_) *δ* 173.8, 171.7, 158.8, 130.3, 128.9, 113.7, 78.0, 74.4, 71.6, 62.1, 55.7, 55.4, 52.7, 45.3, 27.1, 22.6, 12.7; HRMS (NSI-FTMS) *m*/*z* [M + H]^+^ calcd for [C_21_H_32_NO_6_S]^+^ 426.1945, found 426.1945. Determination of ee was carried out by HPLC using an AD-H Chiralpak column, 90 : 10 hexane/IPA, 230 nm, 0.8 mL min^−1^, 25 °C.

### (2*R*,3*R*,4*R*)-Methyl 3-hydroxy-2-(hydroxymethyl)-1-(4-methoxybenzyl)-4-methyl-4-(methylthio)-5-oxopyrrolidine-2-carboxylate 13 (ref. 18)

Alcohol 32a (0.0427 g, 0.1 mmol) was dissolved in anhydrous DCM (0.21 mL) in a flame-dried flask. TFA (0.21 mL) was added, and the mixture stirred under an atmosphere of nitrogen until TLC showed complete consumption of starting material (about 1.5 h). The mixture was diluted with DCM (20 mL) and washed with water (20 mL). The aqueous layer was extracted with DCM (20 mL), and the combined organic layers were washed with saturated aqueous NaHCO_3_ (20 mL) and brine (20 mL), dried (sodium sulfate), filtered, and the solvents removed under reduced pressure. The residue was purified by column chromatography using petroleum ether (40–60 °C)/ethyl acetate 1 : 1 as eluent to yield 13 as a colourless solid (0.0279 g, 75%). Mp 128–130 °C (lit^18^ 129 °C); [*α*]^23^_D_ = −33.84 (*c* 0.13, CHCl_3_) (lit^18^ [*α*]^23^_D_ = −41.8 (*c* 0.1, CHCl_3_)) *ν*_max_ (neat)/cm^−1^: 3416, 2925, 2852, 1737, 1675; ^1^H NMR (500 MHz, CDCl_3_) *δ*_H_ 7.29 (d, *J* = 8.5 Hz, 2H), 6.85 (d, *J* = 8.7 Hz, 2H), 5.11 (d, *J* = 15.3 Hz, 1H), 4.13 (d, *J* = 7.7 Hz, 1H), 4.05 (d, *J* = 15.3 Hz, 1H), 3.85–3.77 (m, 5H), 3.76 (s, 3H), 3.67 (d, *J* = 8.1 Hz, 1H), 2.14 (s, 3H), 1.61 (s, 3H); ^13^C NMR (126 MHz, CDCl_3_) *δ*_C_ 173.5, 171.6, 159.5, 129.8, 129.6, 114.6, 76.8, 72.4, 62.5, 55.4, 53.4, 52.9, 44.8, 22.9, 12.4; HRMS (NSI-FTMS) *m*/*z* [M + H]^+^ calcd for [C_17_H_24_NO_6_S]^+^ 370.1319, found 370.1320. Determination of ee was carried out by HPLC using an AD-H Chiralpak column, 80 : 20 hexane/IPA, 230 nm, 0.8 mL min^−1^, 25 °C.

## Conflicts of interest

There are no conflicts to declare.

## Supplementary Material

RA-009-C9RA07244F-s001

RA-009-C9RA07244F-s002
