# Supplementary material for: Formal synthesis of (+)-lactacystin from l-serine
Source: RSC Adv. 2019 Sep 24;9(51):30019–32. doi: 10.1039/c9ra07244f (PMC9072140; doi:10.1039/c9ra07244f)
Supplement: RA-009-C9RA07244F-s001 [file RA-009-C9RA07244F-s001.pdf]

Electronic Supplementary Material (ESI) for RSC Advances.
This journal is © The Royal Society of Chemistry 2019

Formal Synthesis of (+)-Lactacystin from L-Serine

Philip C. Bulman Page,* Ross L. Goodyear, Yohan Chan, Alexandra M. Z. Slawin, Steven M. Allin

Supplementary information

S2-S26 NMR spectra

S27-S41 HPLC traces

S42-S67 X-Ray data

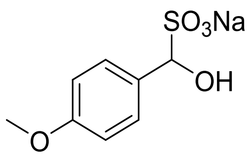
SO3Na
OH
O

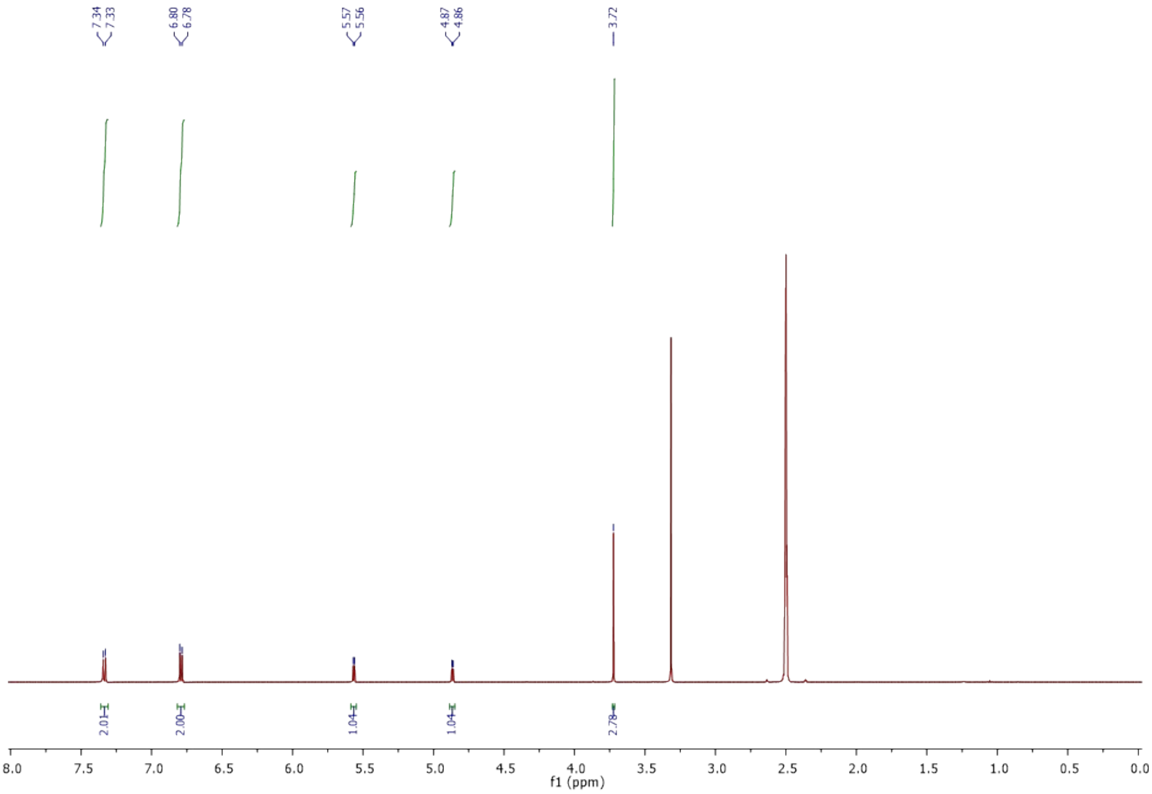
7.34
7.33
6.80
6.78
5.57
5.56
4.87
4.86
3.72
2.01
2.00
1.04
1.04
2.78
f1 (ppm)

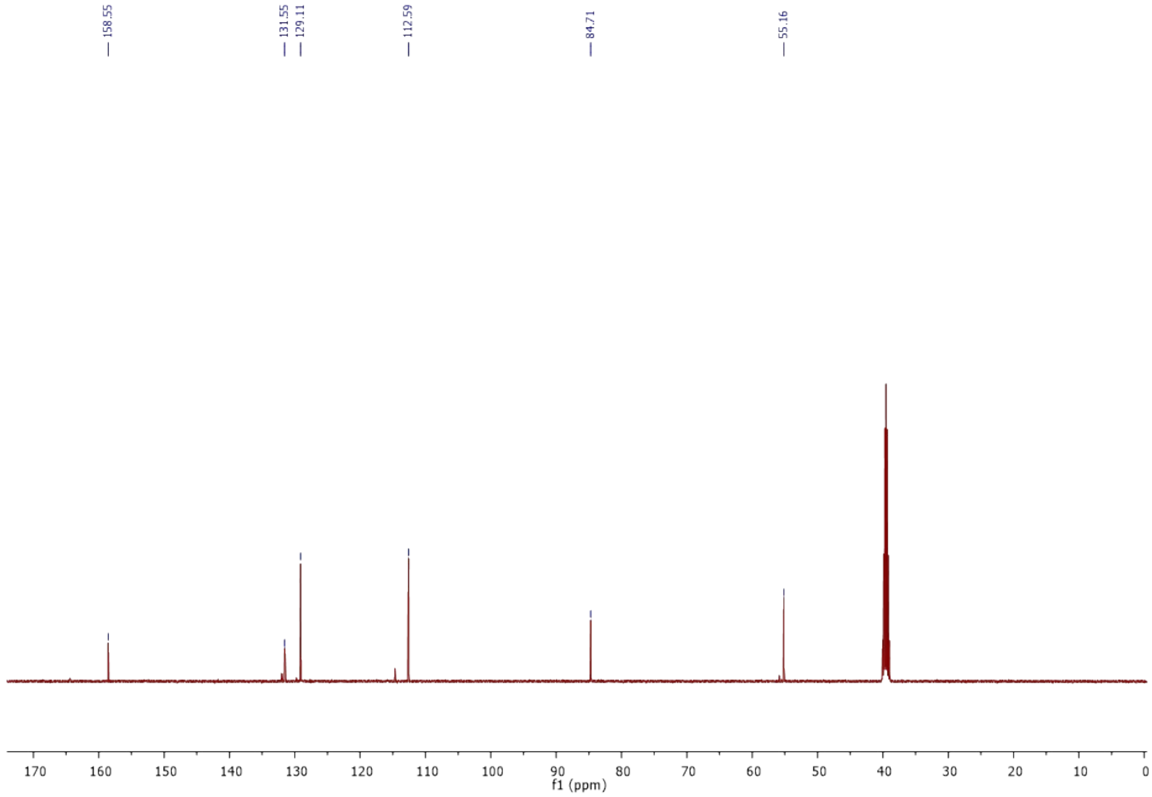
158.55
131.55
129.11
112.59
84.71
55.16
f1 (ppm)

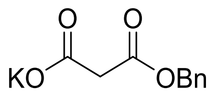
O
O
KO
OBn

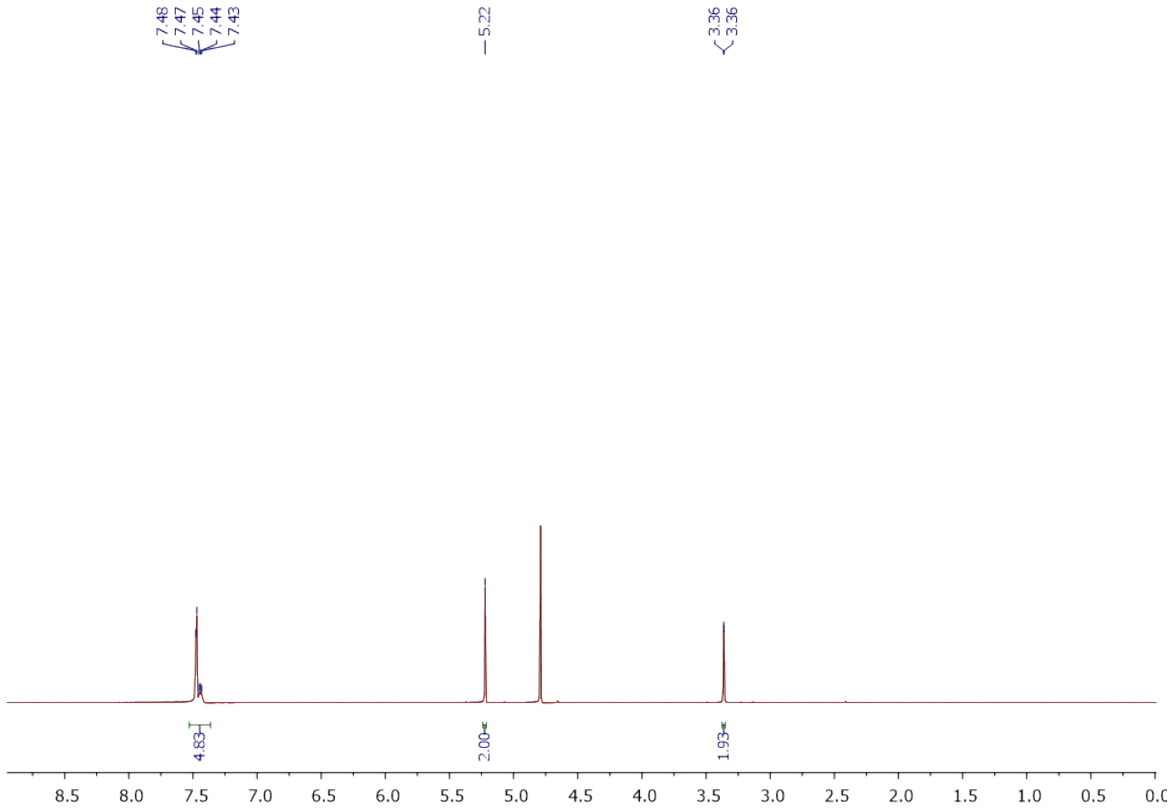
7.48
7.47
7.45
7.44
7.43
5.22
3.36
3.36
4.83
2.00
1.93
8.5 8.0 7.5 7.0 6.5 6.0 5.5 5.0 4.5 4.0 3.5 3.0 2.5 2.0 1.5 1.0 0.5 0.0

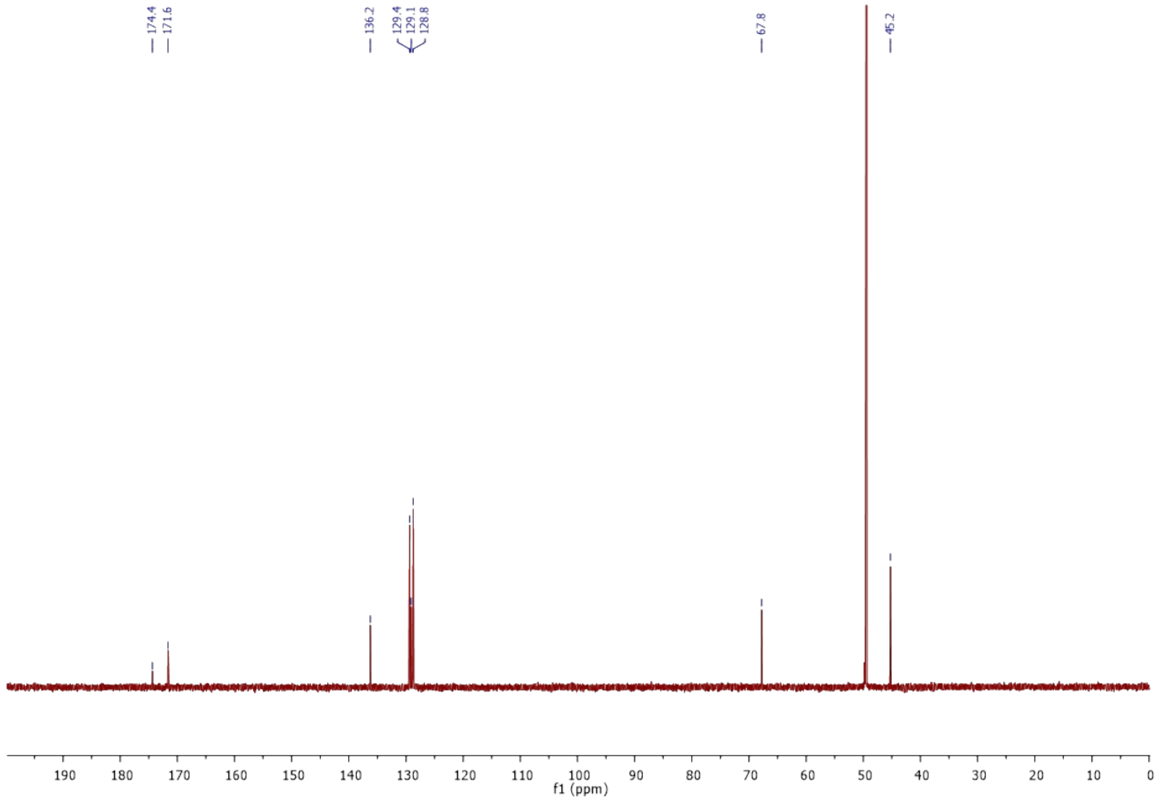
174.4
171.6
136.2
129.4
129.1
128.8
67.8
45.2
190 180 170 160 150 140 130 120 110 100 90 80 70 60 50 40 30 20 10 0
f1 (ppm)

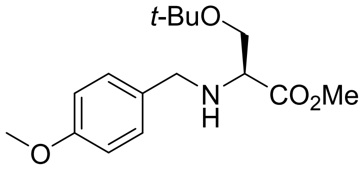
t-BuO
N
H
CO2Me
O

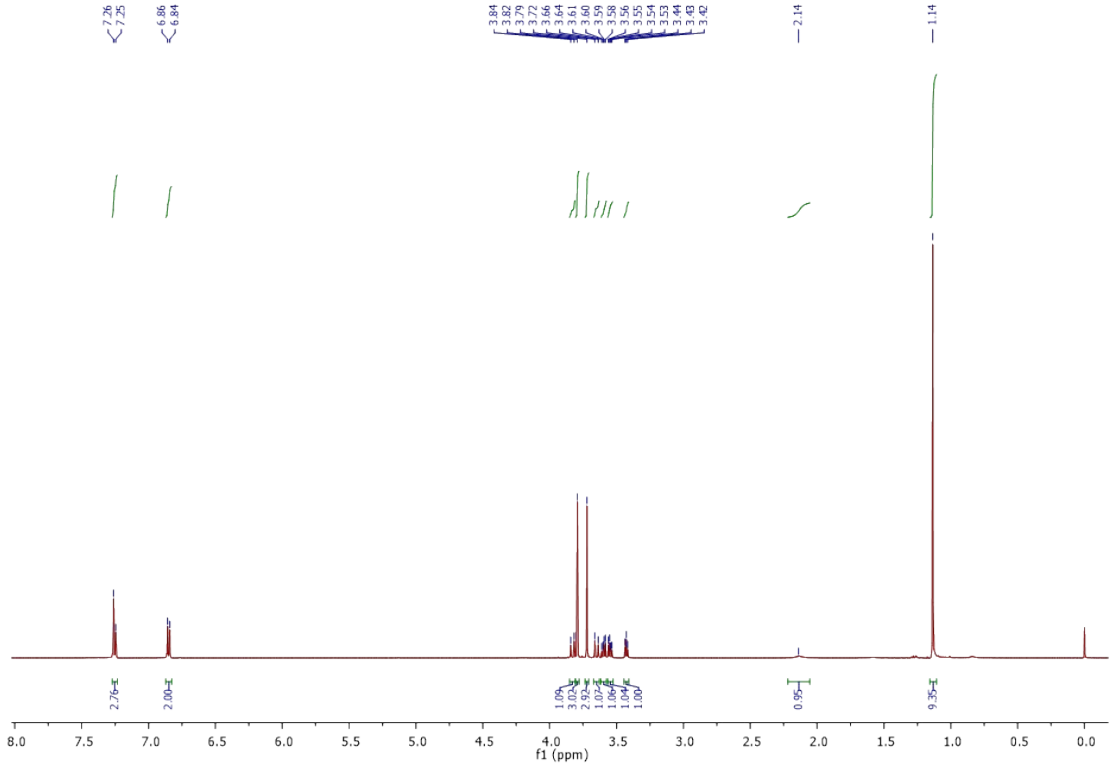
7.26
7.25
6.86
6.84
3.84
3.82
3.79
3.72
3.66
3.64
3.61
3.60
3.59
3.58
3.56
3.55
3.54
3.53
3.44
3.43
3.42
2.14
1.14
2.76
2.00
1.09
3.02
2.92
1.07
1.06
1.04
1.00
0.95
9.35
f1 (ppm)

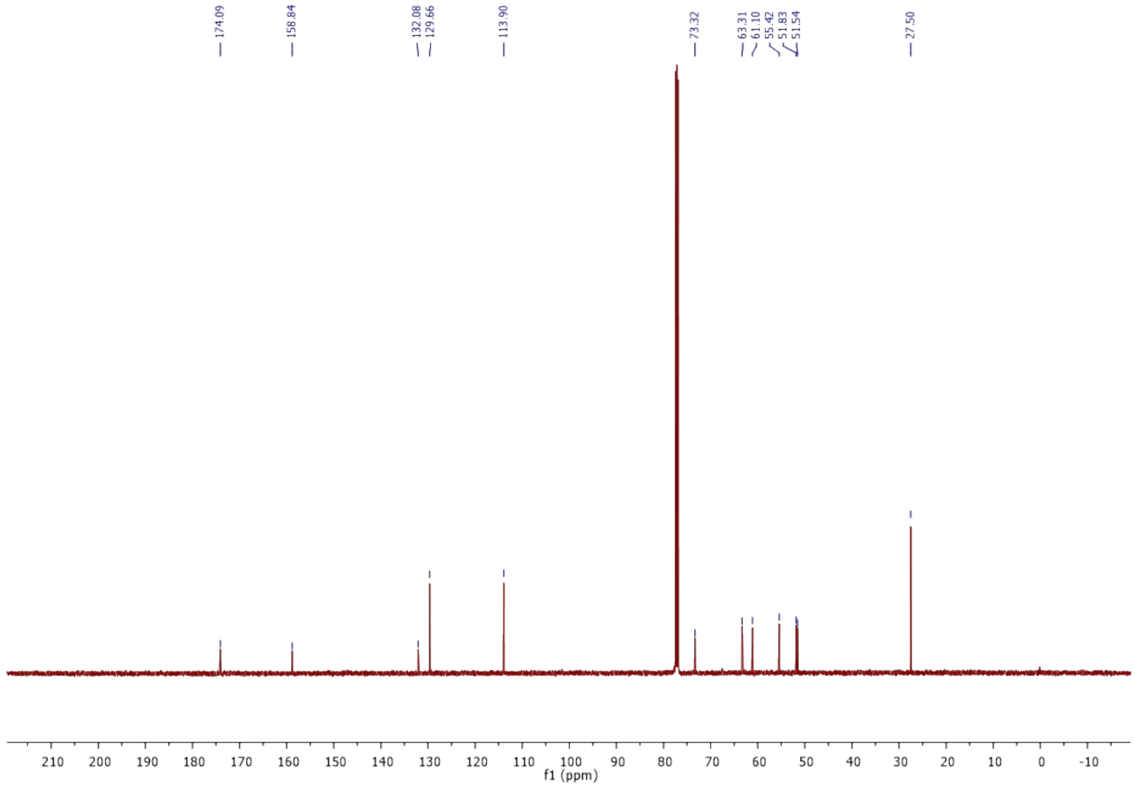
174.09
158.84
132.08
129.66
113.90
73.32
63.31
61.10
55.42
51.83
51.54
27.50
f1 (ppm)

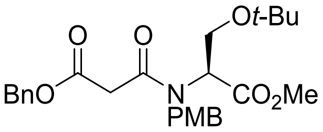
Ot-Bu
O
O
BnO
N
PMB
CO2Me

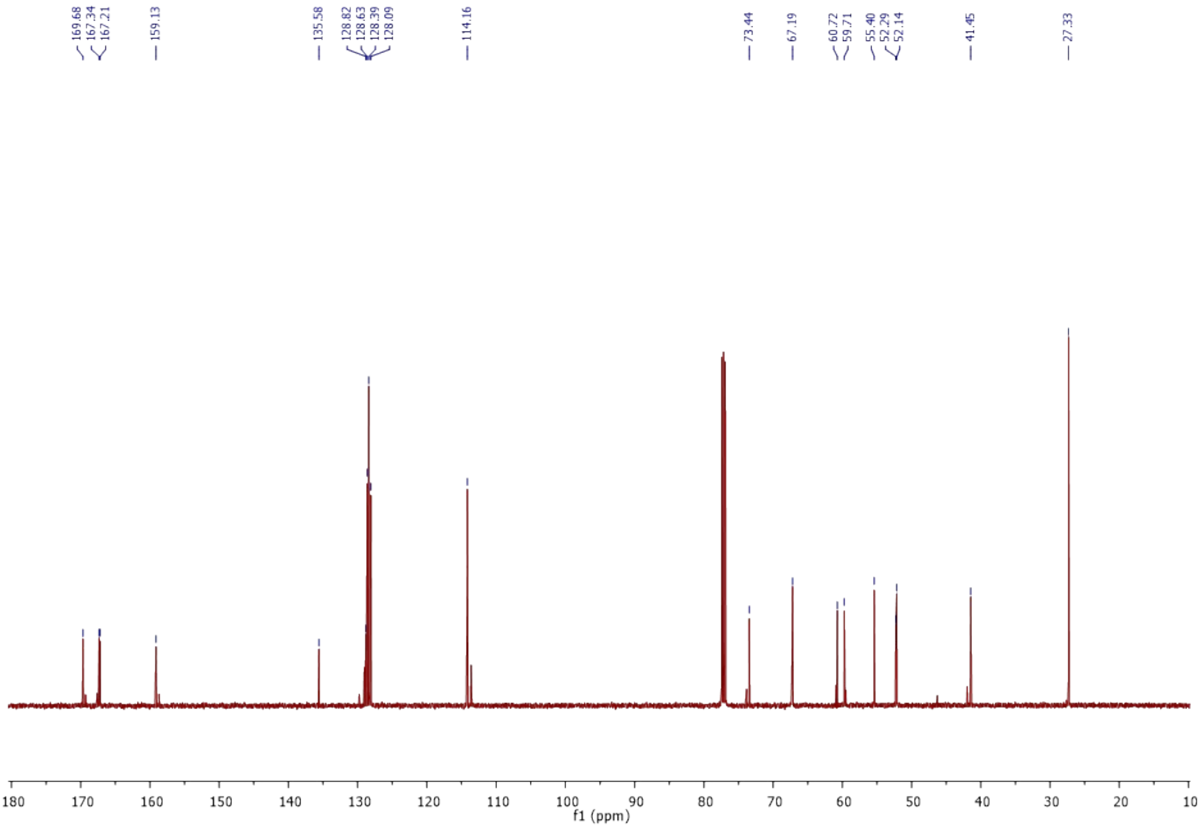
169.68
167.34
167.21
159.13
135.58
128.82
128.63
128.39
128.09
114.16
73.44
67.19
60.72
59.71
55.40
52.29
52.14
41.45
27.33
f1 (ppm)

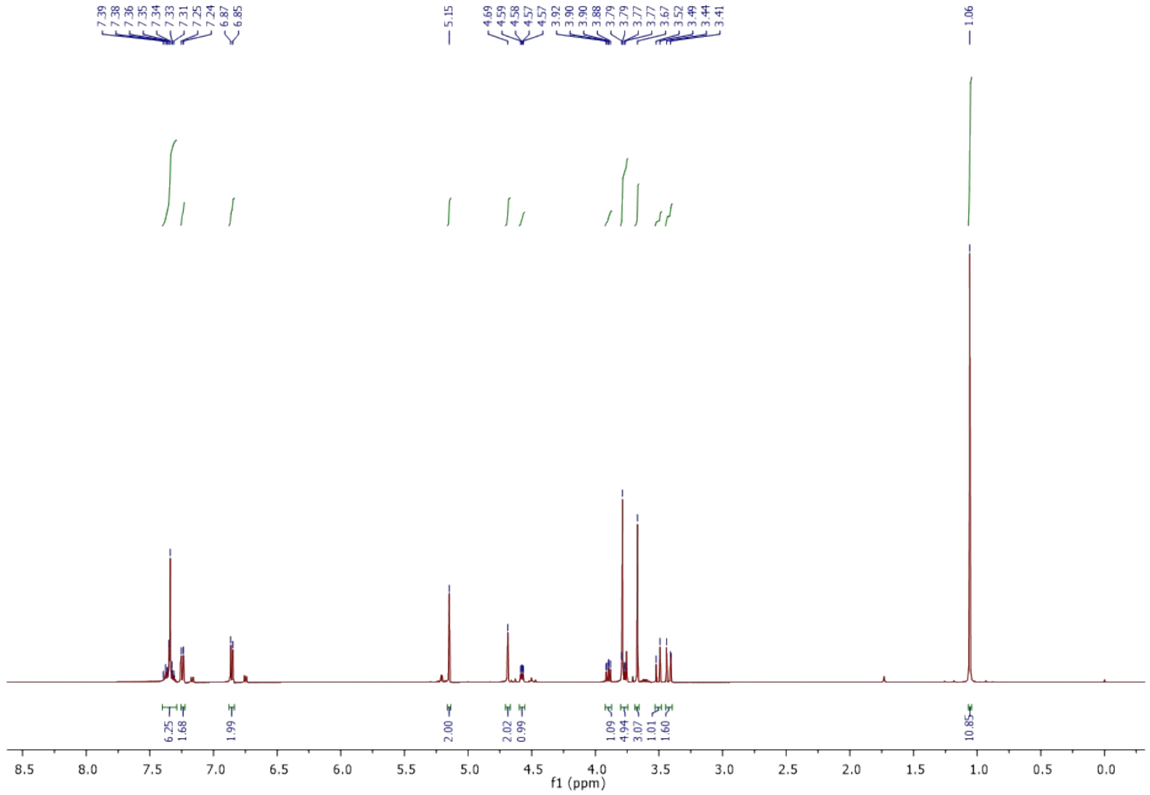
7.39
7.38
7.36
7.35
7.34
7.33
7.31
7.25
7.24
6.87
6.85
5.15
4.69
4.59
4.58
4.57
4.57
3.92
3.90
3.88
3.79
3.79
3.77
3.77
3.67
3.52
3.49
3.44
3.41
1.06
6.25
1.68
1.99
2.00
2.02
0.99
1.09
4.94
3.07
1.01
1.60
10.85
f1 (ppm)

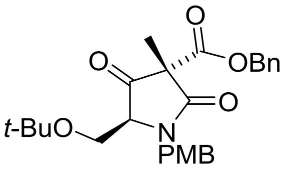

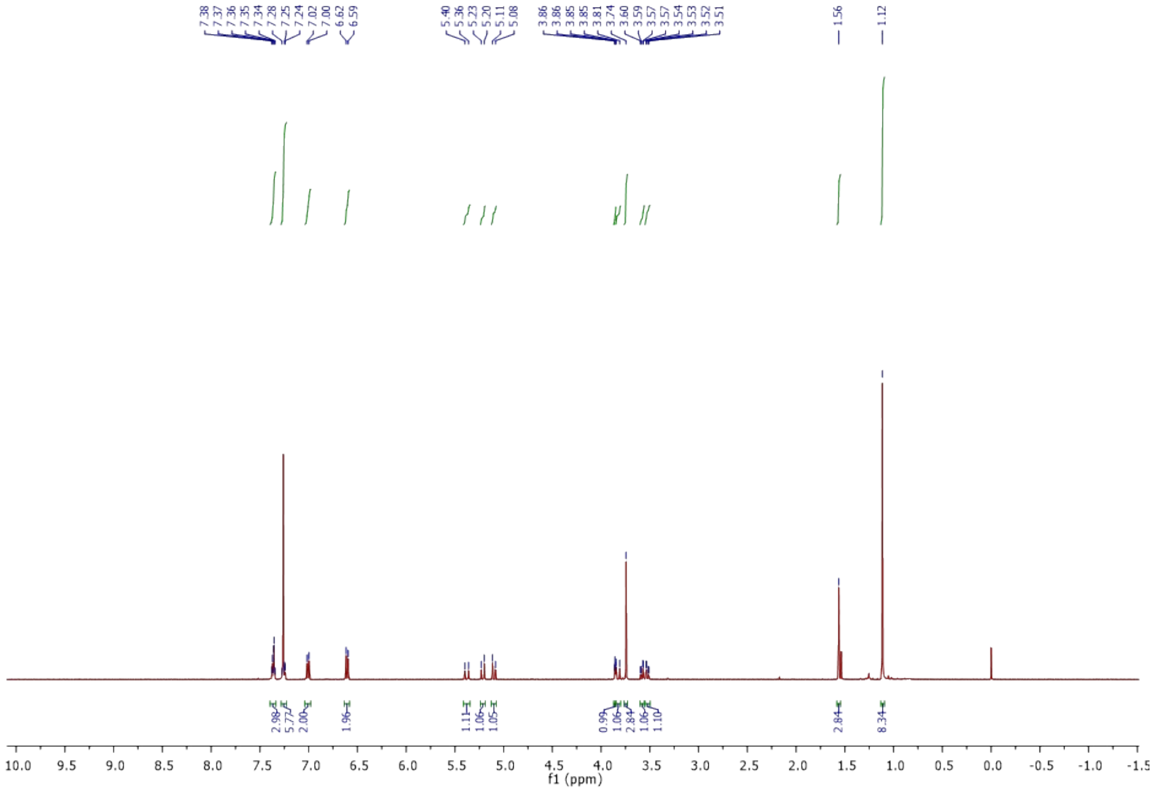

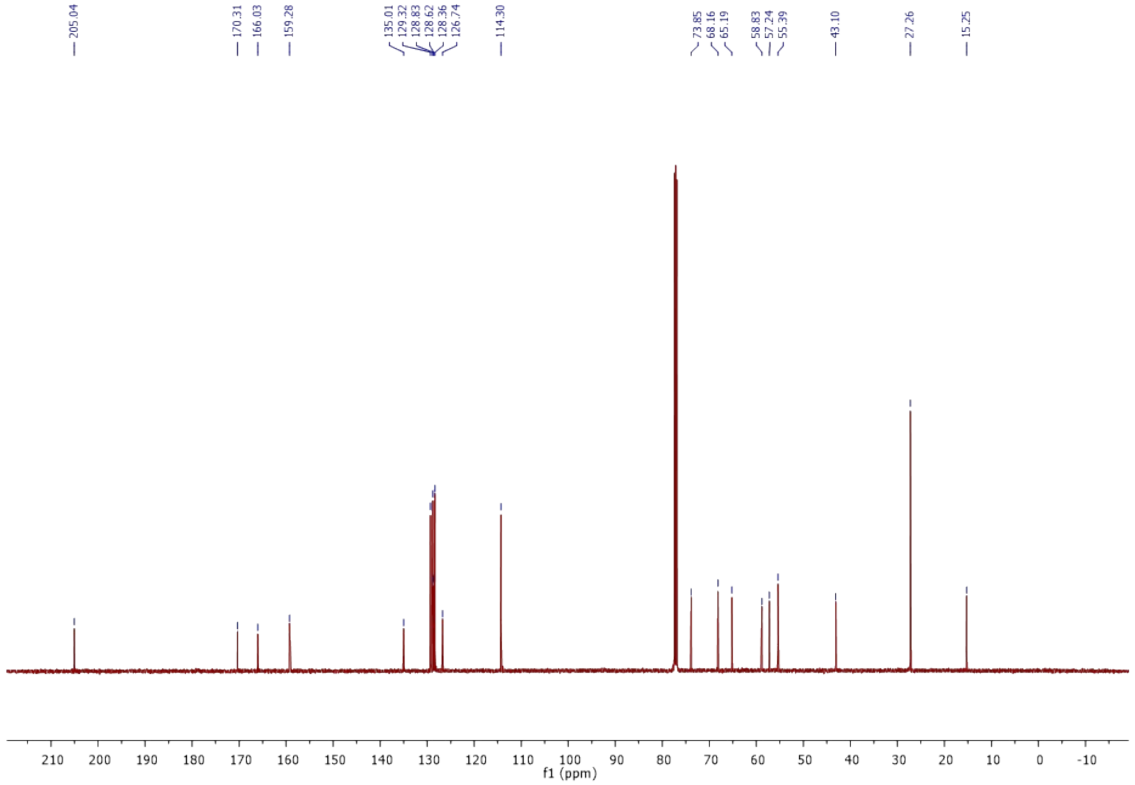

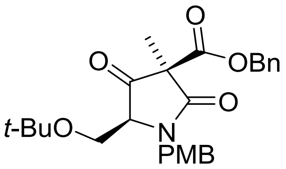

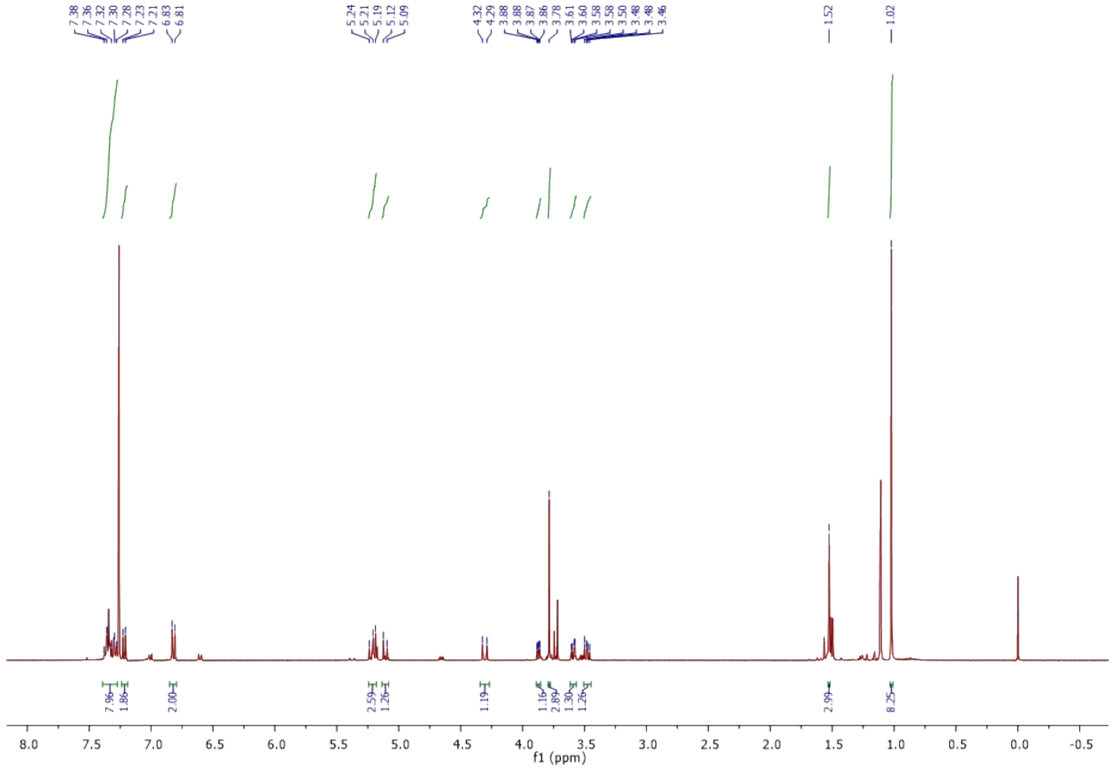
7.38
7.36
7.32
7.30
7.28
7.23
7.21
6.83
6.81
5.24
5.21
5.19
5.12
5.09
4.32
4.29
3.88
3.88
3.87
3.86
3.78
3.61
3.60
3.58
3.58
3.50
3.48
3.48
3.46
1.52
1.02
7.96
1.86
2.00
2.59
1.26
1.19
1.16
2.89
1.30
1.26
2.99
8.25
f1 (ppm)

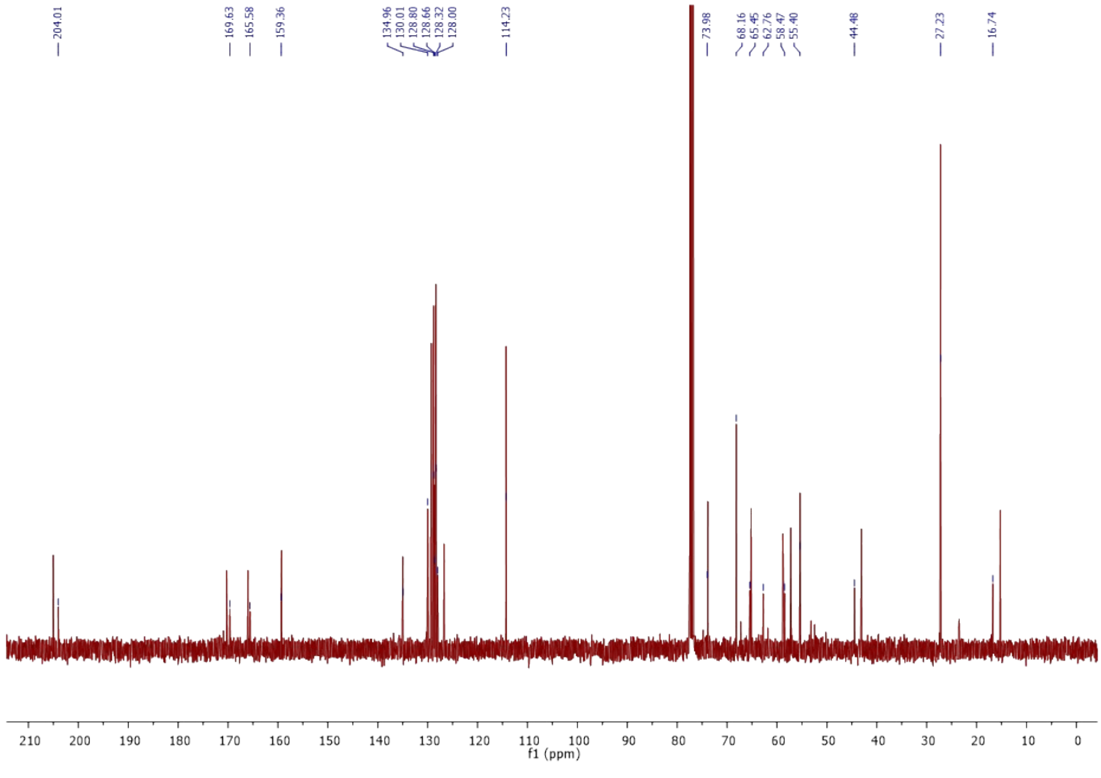
204.01
169.63
165.58
159.36
134.96
130.01
128.80
128.66
128.32
128.00
114.23
73.98
68.16
65.45
62.76
58.47
55.40
44.48
27.23
16.74
f1 (ppm)

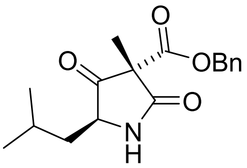

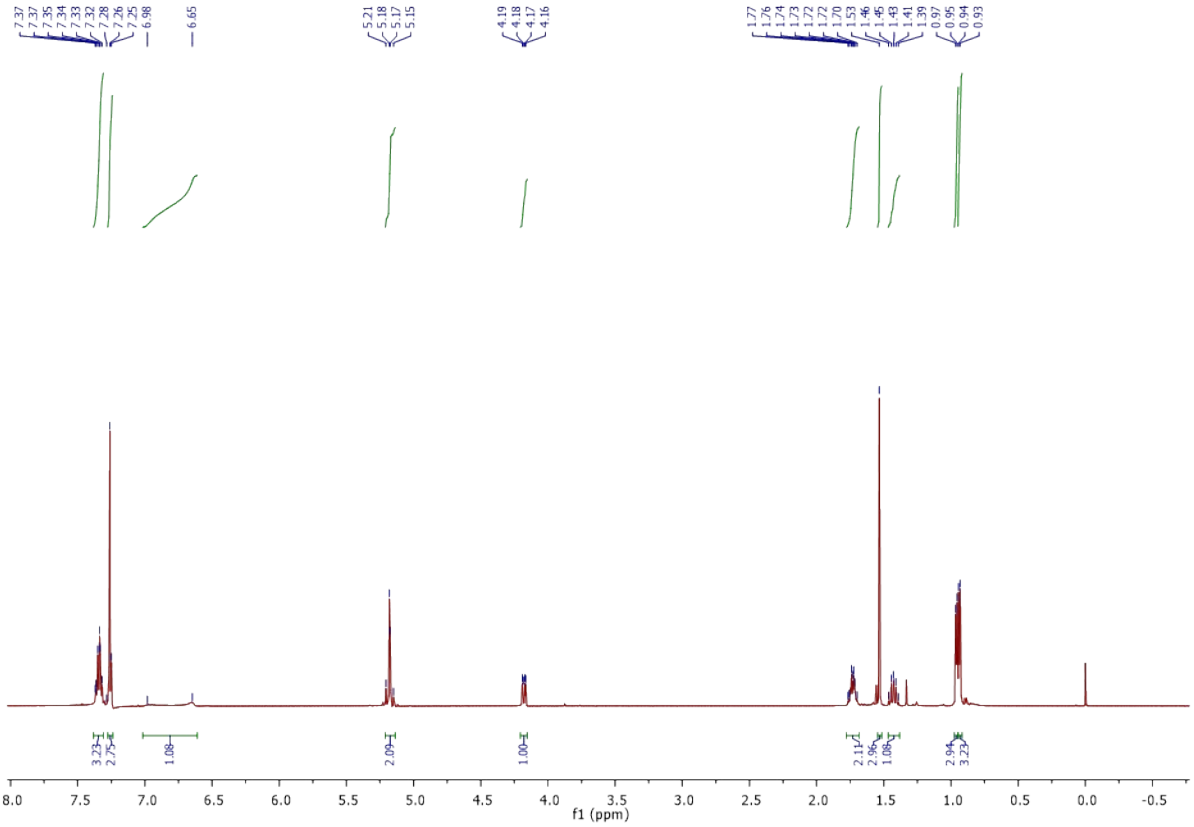

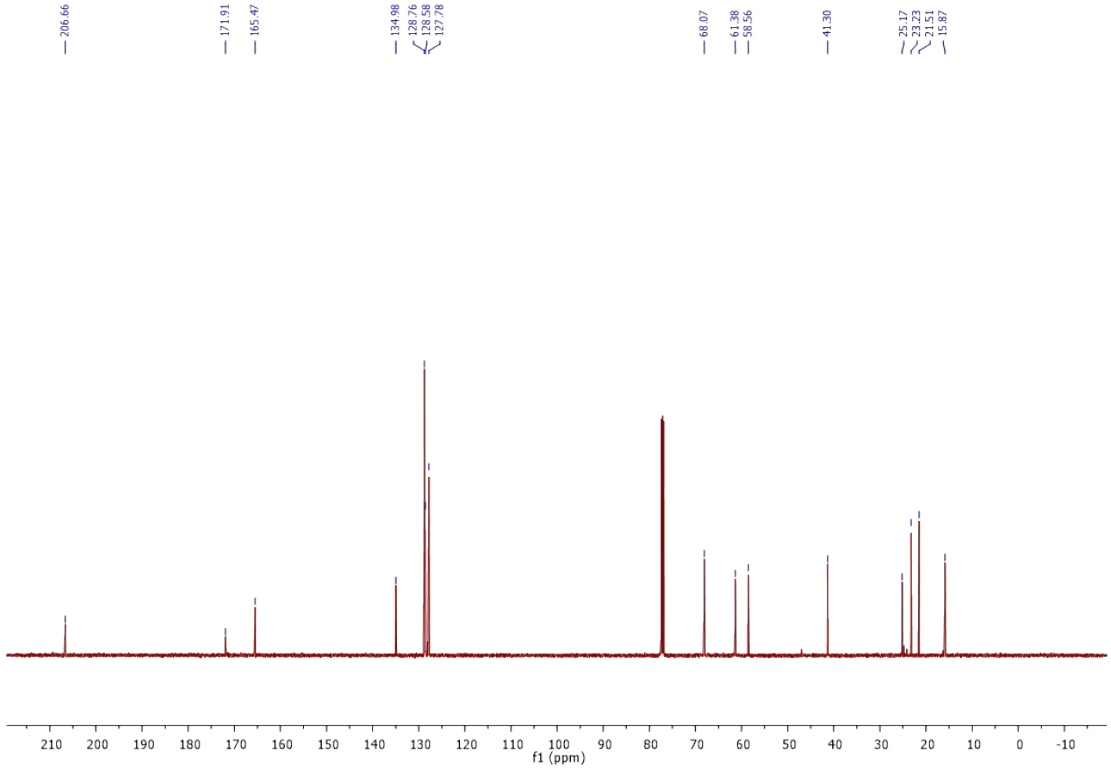

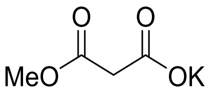
MeO
OK
O
O

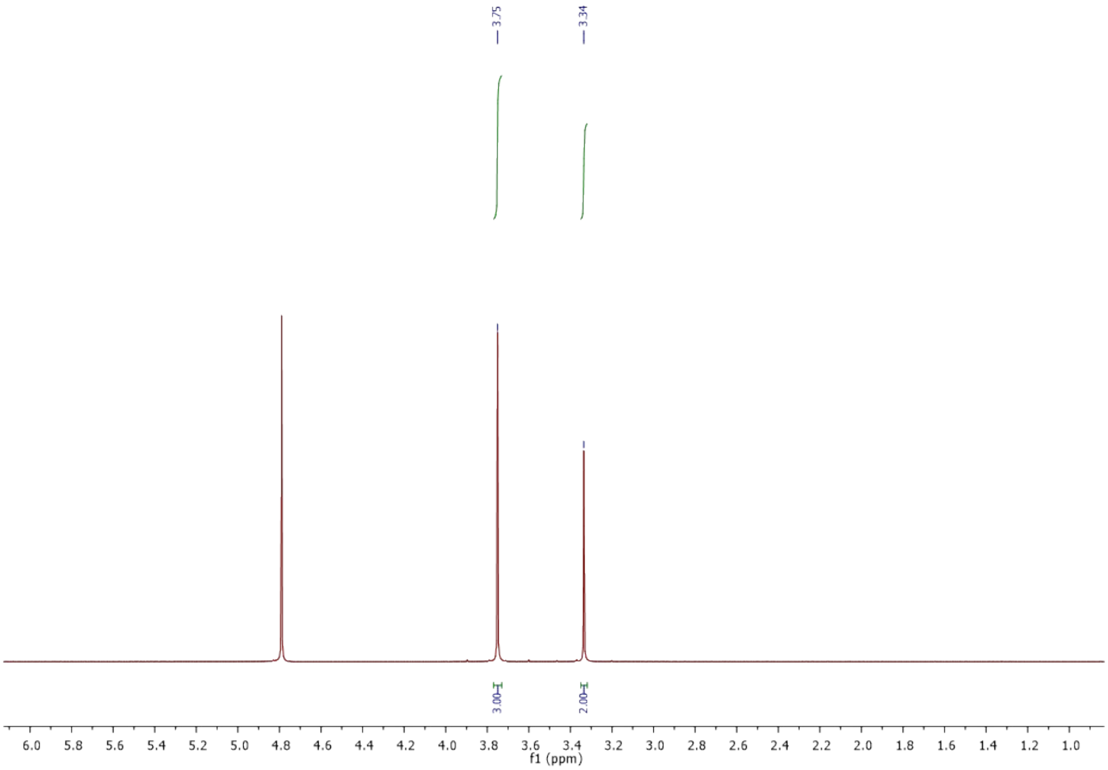
3.75
3.34
3.00
2.00
f1 (ppm)

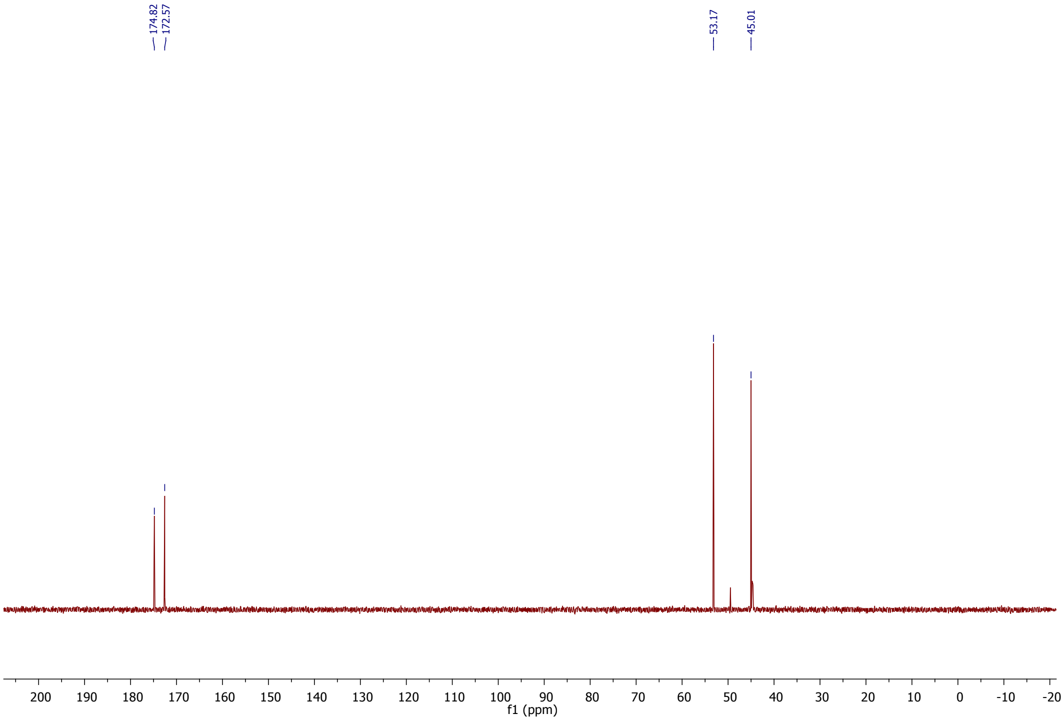
174.82
172.57
53.17
45.01
f1 (ppm)

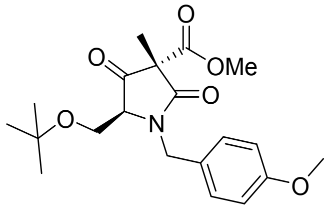
O
OMe
O
O
N
O
O

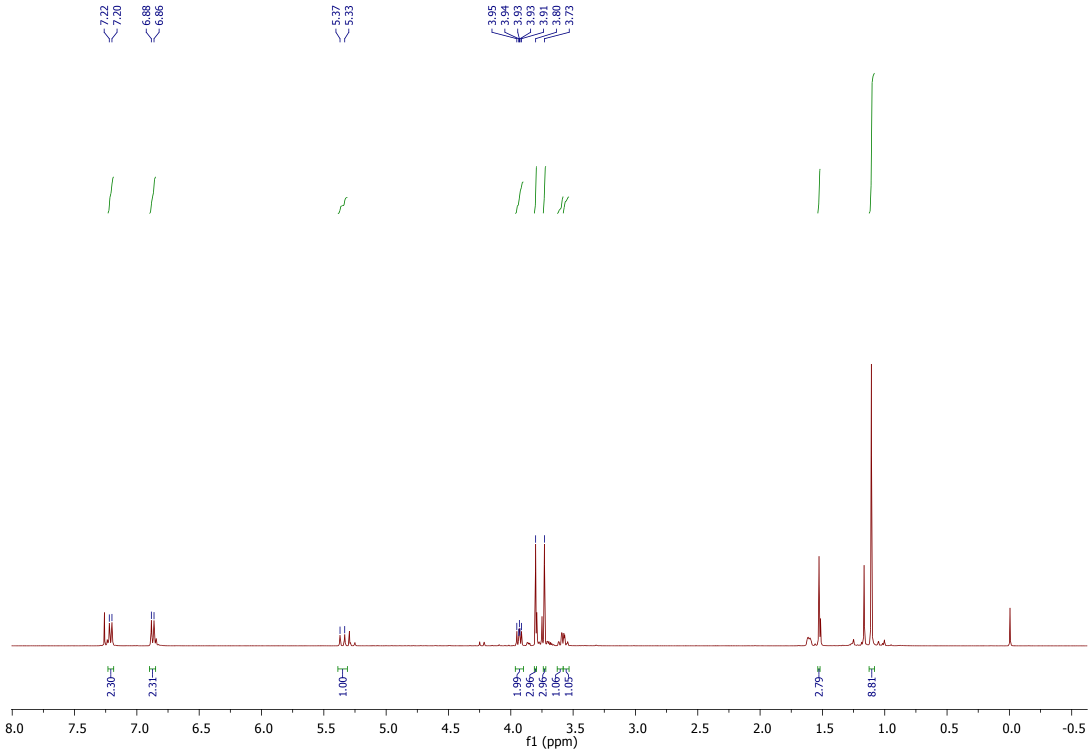
7.22
7.20
6.88
6.86
5.37
5.33
3.95
3.94
3.93
3.93
3.91
3.80
3.73
2.30
2.31
1.00
1.99
2.96
2.96
1.06
1.05
2.79
8.81
8.0 7.5 7.0 6.5 6.0 5.5 5.0 4.5 4.0 3.5 3.0 2.5 2.0 1.5 1.0 0.5 0.0 -0.5
f1 (ppm)

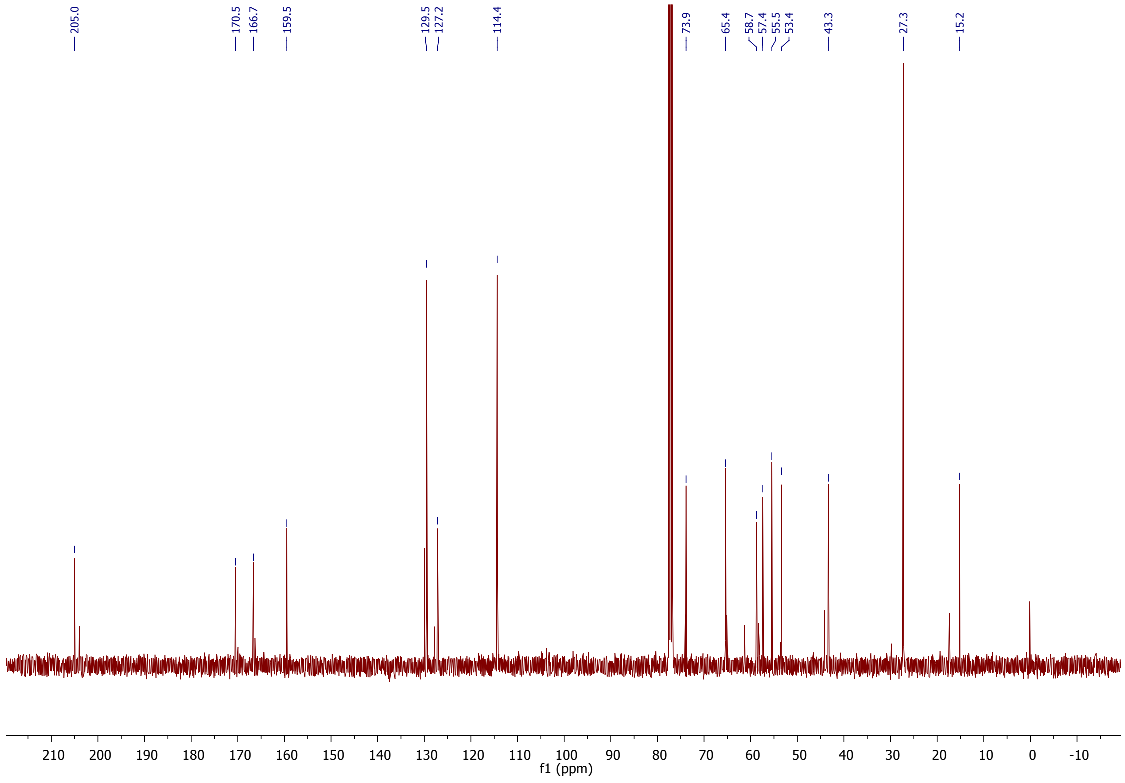
205.0
170.5
166.7
159.5
129.5
127.2
114.4
73.9
65.4
58.7
57.4
55.5
53.4
43.3
27.3
15.2
210 200 190 180 170 160 150 140 130 120 110 100 90 80 70 60 50 40 30 20 10 0 -10
f1 (ppm)

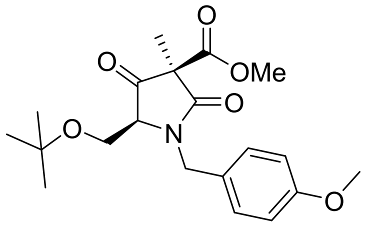
O
OMe
O
O
N
O
O

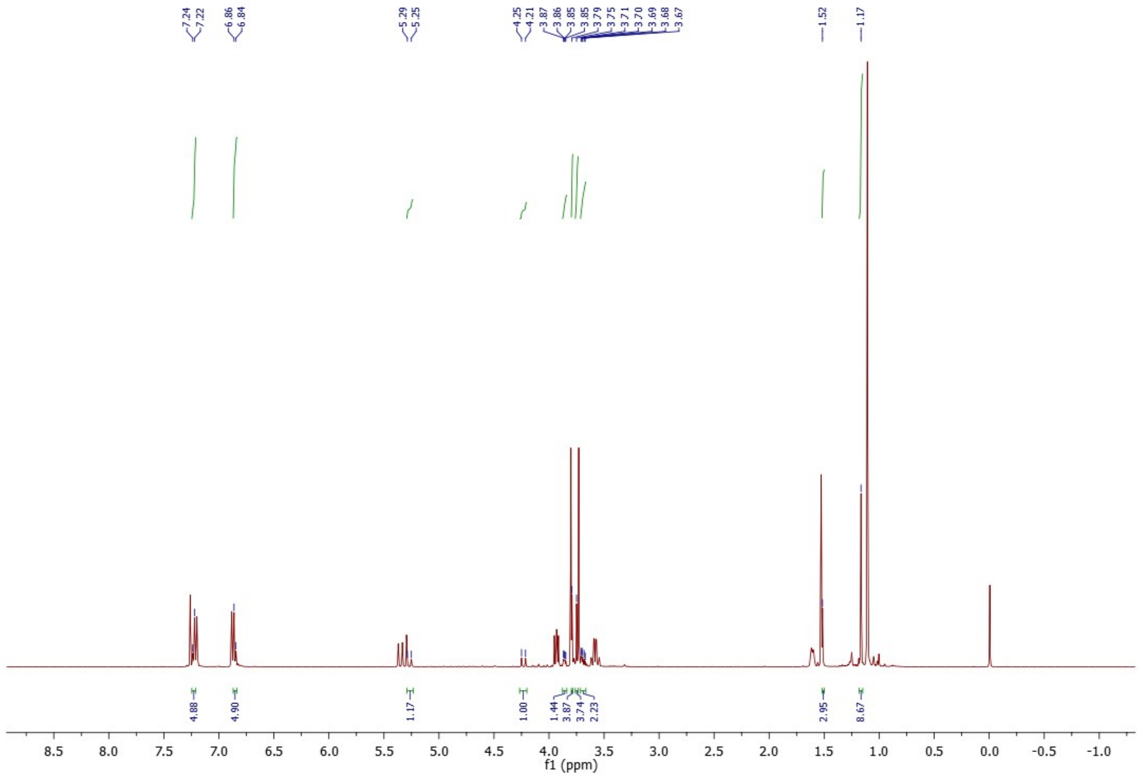
7.24
7.22
6.86
6.84
5.29
5.25
4.25
4.21
3.87
3.86
3.85
3.85
3.79
3.75
3.71
3.70
3.69
3.68
3.67
1.52
1.17
4.88
4.90
1.17
1.00
1.44
3.87
3.74
2.23
2.95
8.67
f1 (ppm)

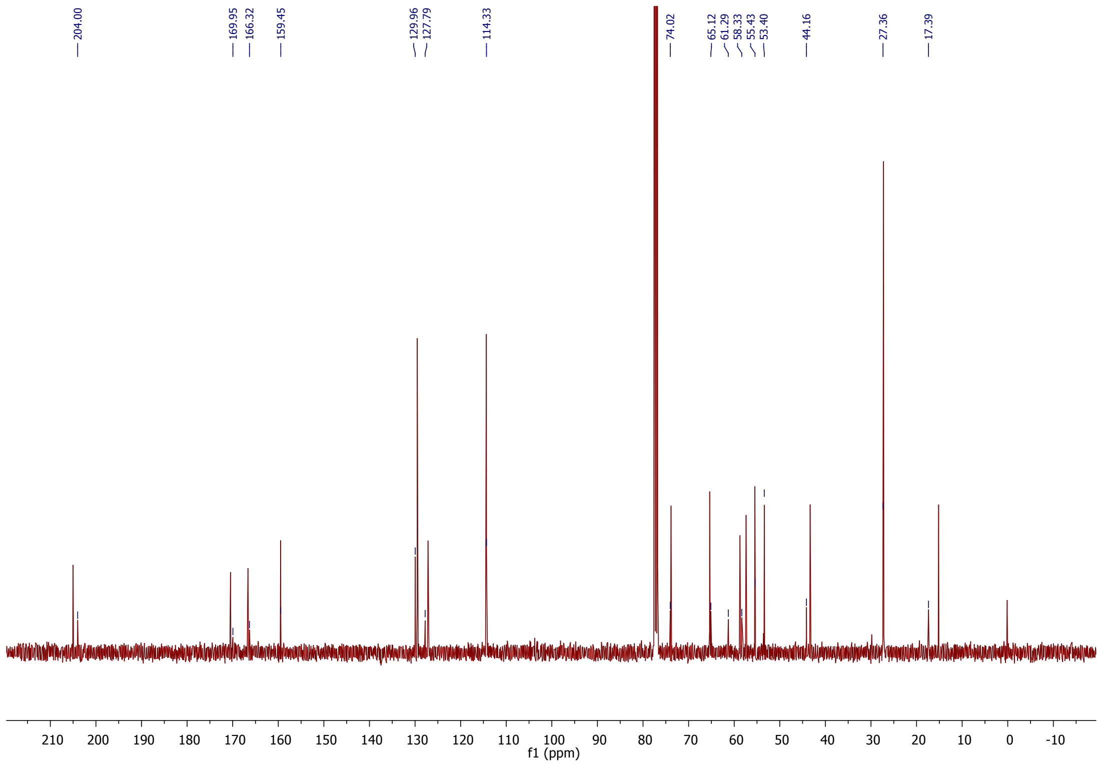
204.00
169.95
166.32
159.45
129.96
127.79
114.33
74.02
65.12
61.29
58.33
55.43
53.40
44.16
27.36
17.39
f1 (ppm)

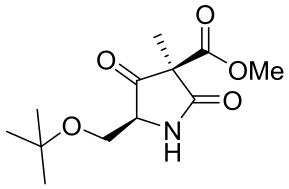
OMe
N
H

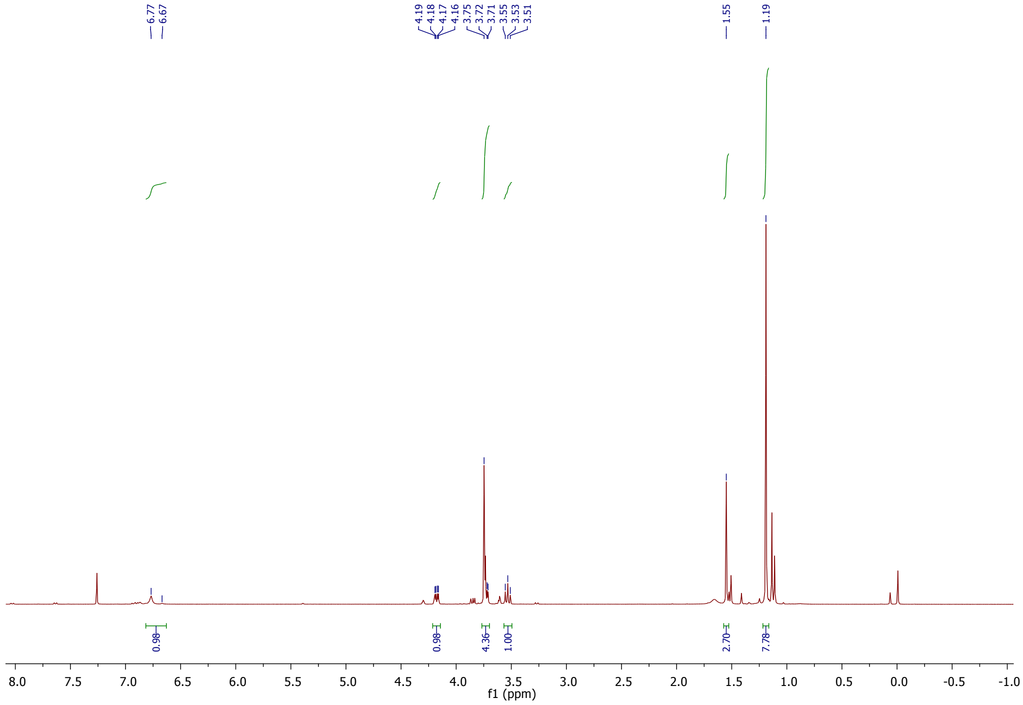
6.77
6.67
4.19
4.18
4.17
4.16
3.75
3.72
3.71
3.55
3.53
3.51
1.55
1.19
0.98
0.98
4.36
1.00
2.70
7.78
f1 (ppm)

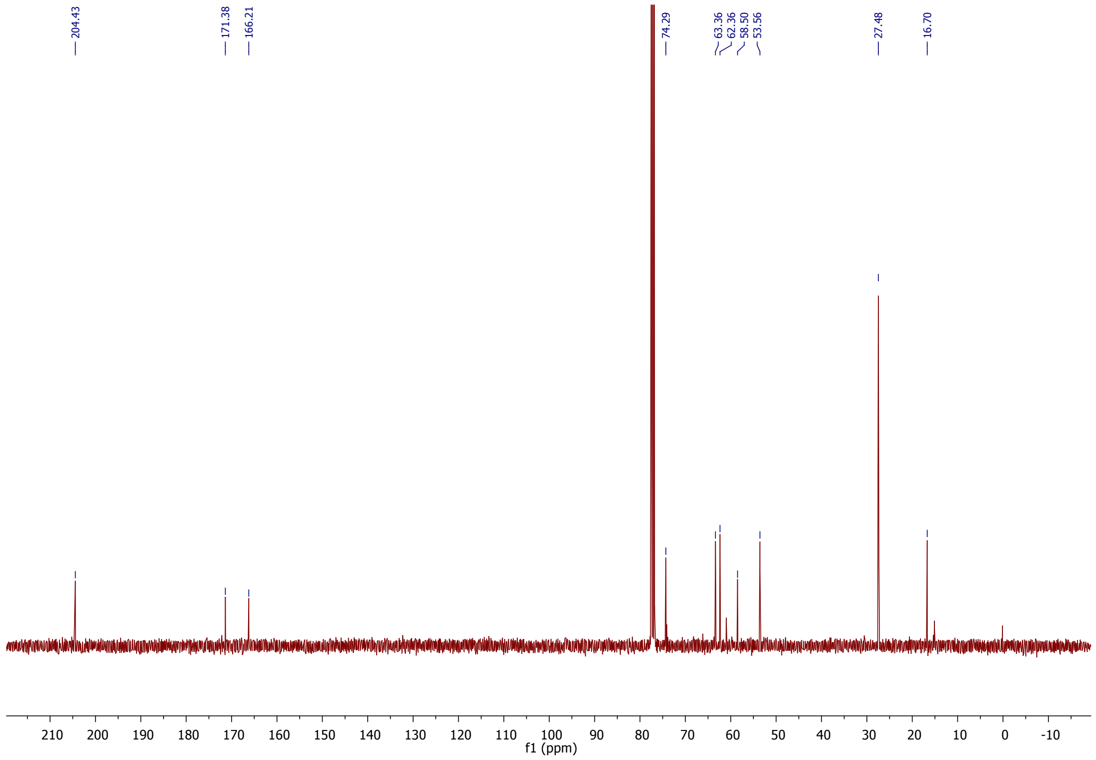
204.43
171.38
166.21
74.29
63.36
62.36
58.50
53.56
27.48
16.70
f1 (ppm)

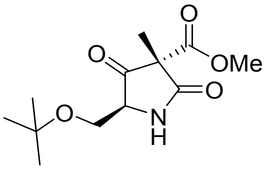
O
OMe
O
O
O
N
H

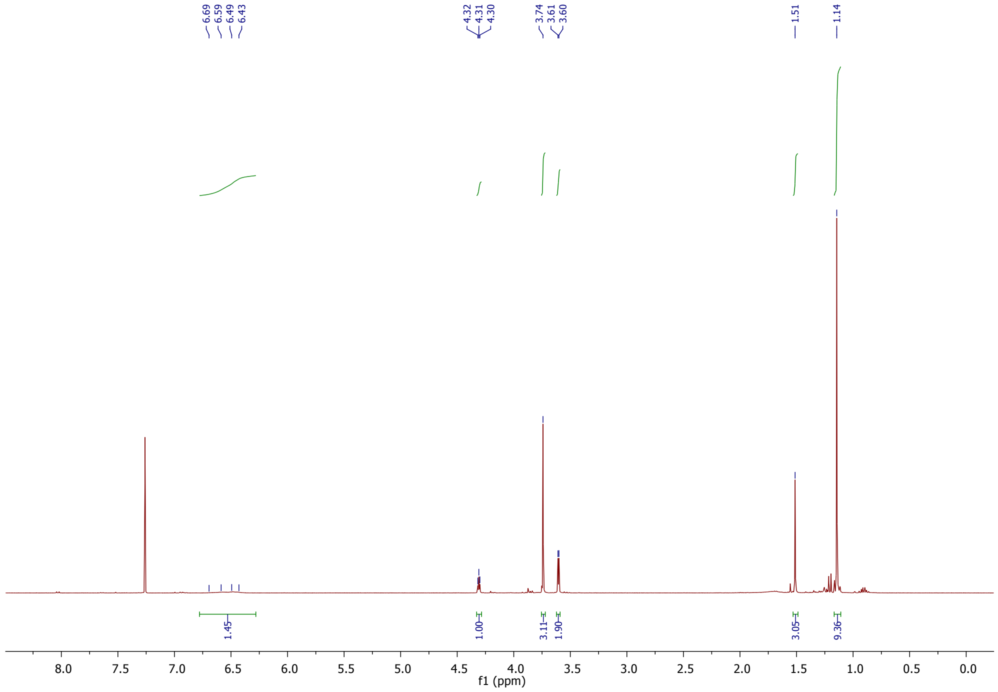
6.69
6.59
6.49
6.43
4.32
4.31
4.30
3.74
3.61
3.60
1.51
1.14
1.45
1.00
3.11
1.90
3.05
9.36
8.0 7.5 7.0 6.5 6.0 5.5 5.0 4.5 4.0 3.5 3.0 2.5 2.0 1.5 1.0 0.5 0.0
f1 (ppm)

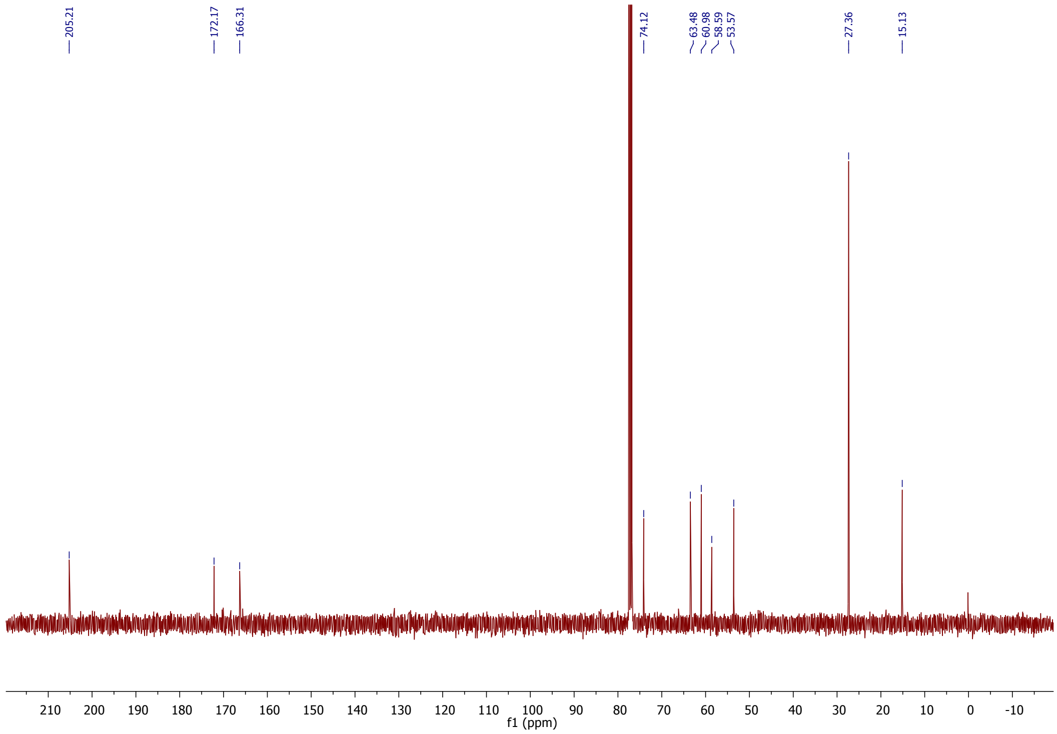
205.21
172.17
166.31
74.12
63.48
60.98
58.59
53.57
27.36
15.13
210 200 190 180 170 160 150 140 130 120 110 100 90 80 70 60 50 40 30 20 10 0 -10
f1 (ppm)

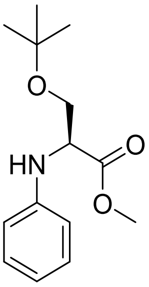
O
HN
O
O

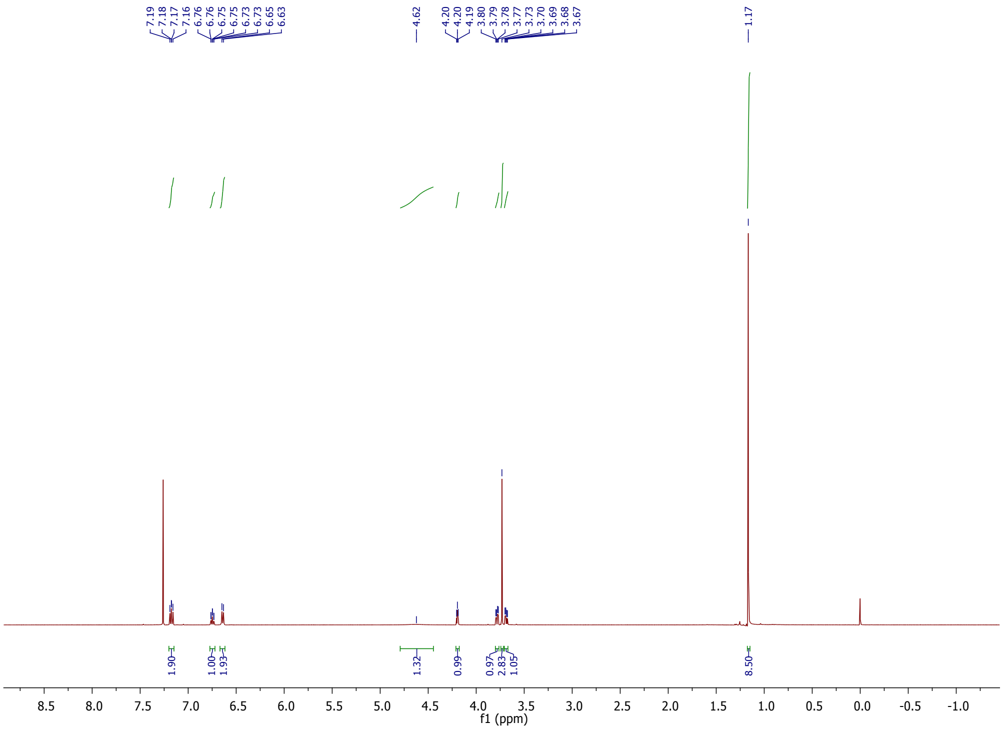
7.19
7.18
7.17
7.16
6.76
6.76
6.75
6.75
6.73
6.73
6.65
6.63
4.62
4.20
4.20
4.19
3.80
3.79
3.78
3.77
3.73
3.73
3.70
3.69
3.68
3.67
1.17
1.90
1.00
1.93
1.32
0.99
0.97
2.83
1.05
8.50
f1 (ppm)

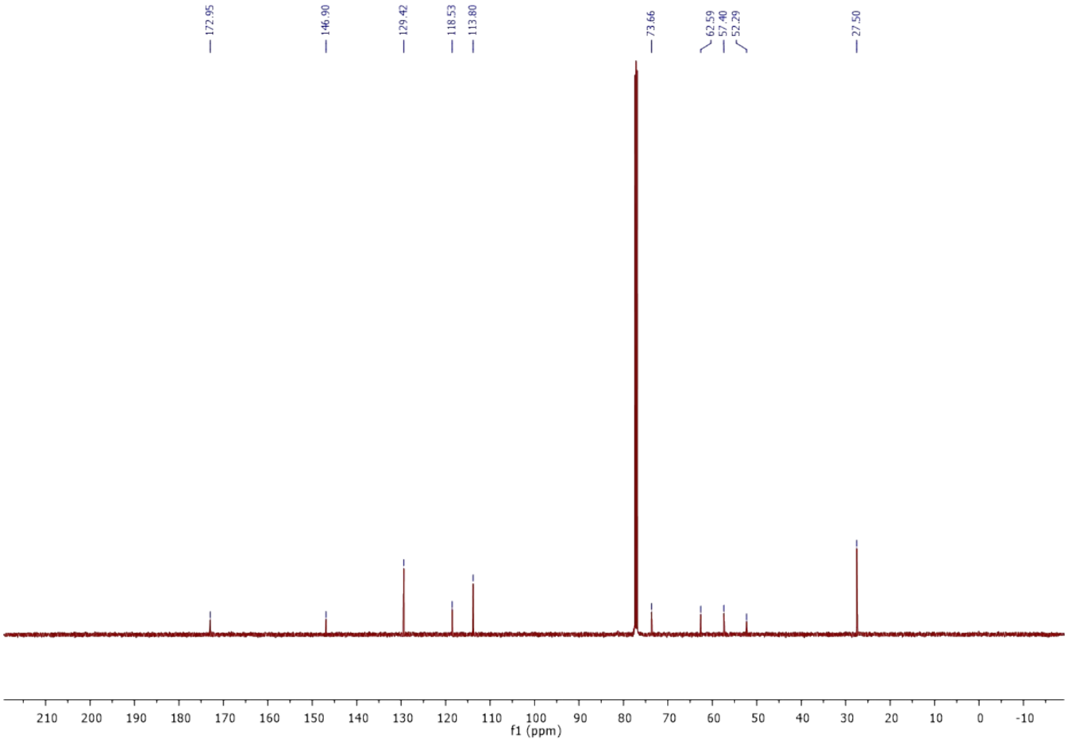
172.95
146.90
129.42
118.53
113.80
73.66
62.59
57.40
52.29
27.50
f1 (ppm)

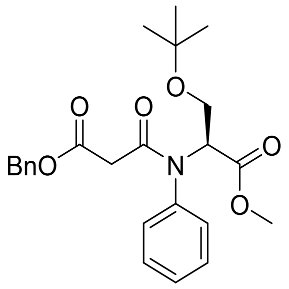
BnO
O
O
N
O
O
O

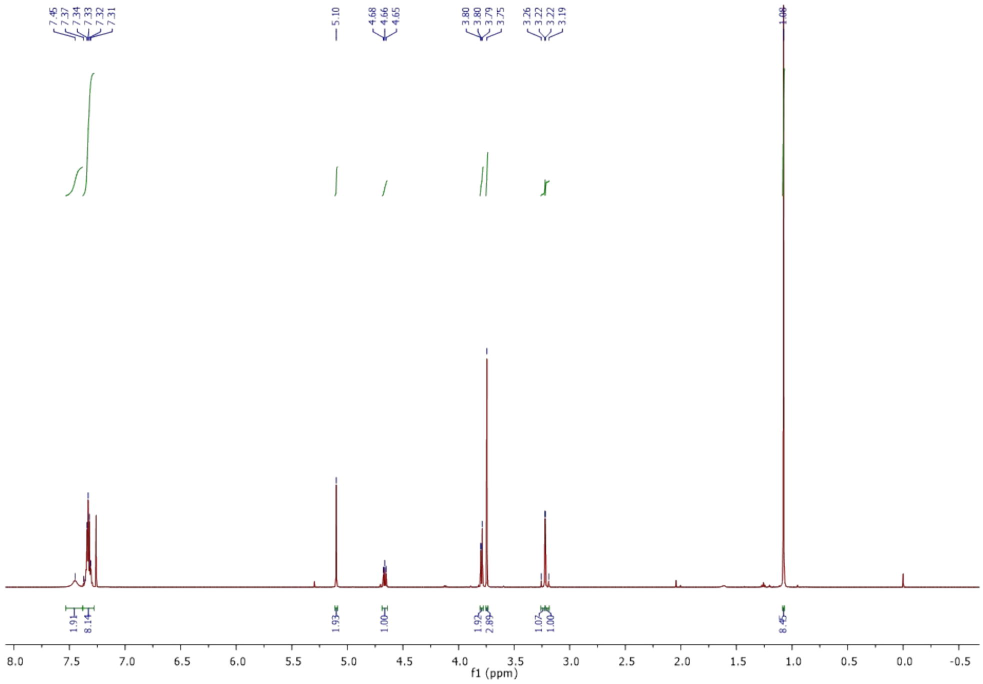
7.45
7.37
7.34
7.33
7.32
7.31
5.10
4.68
4.66
4.65
3.80
3.80
3.79
3.75
3.26
3.22
3.22
3.19
1.91
8.14
1.93
1.00
1.92
2.89
1.07
1.00
8.45
f1 (ppm)

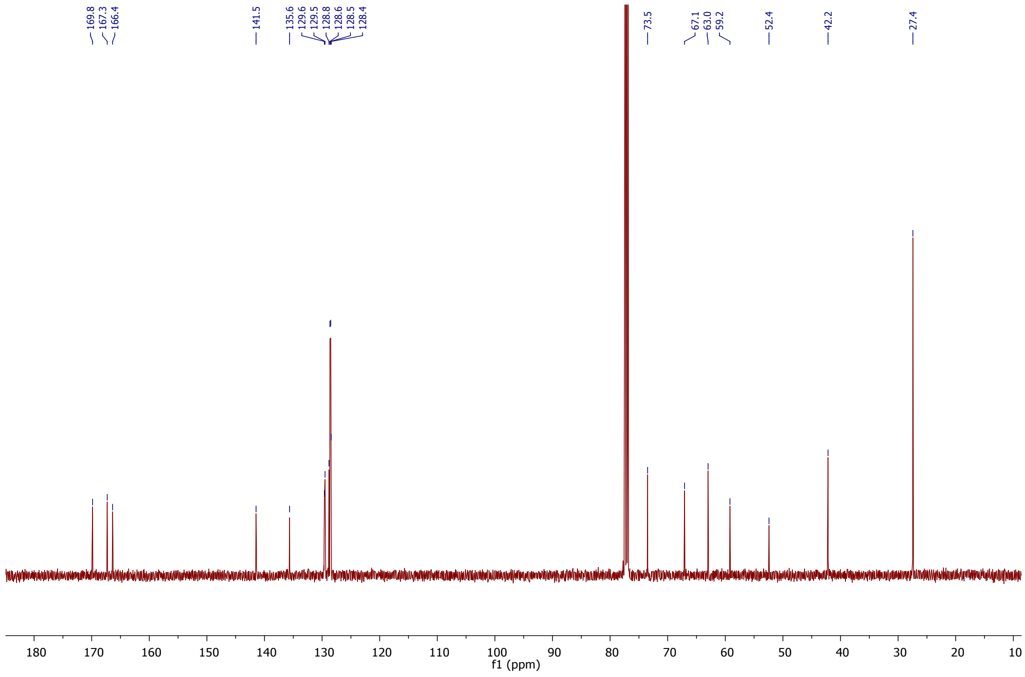
169.8
167.3
166.4
141.5
135.6
129.6
129.5
128.8
128.6
128.5
128.4
73.5
67.1
63.0
59.2
52.4
42.2
27.4
f1 (ppm)

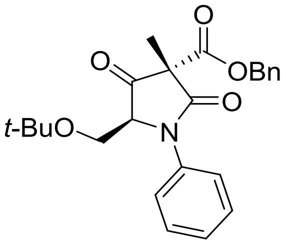
O
OBn
O
O
t-BuO
N

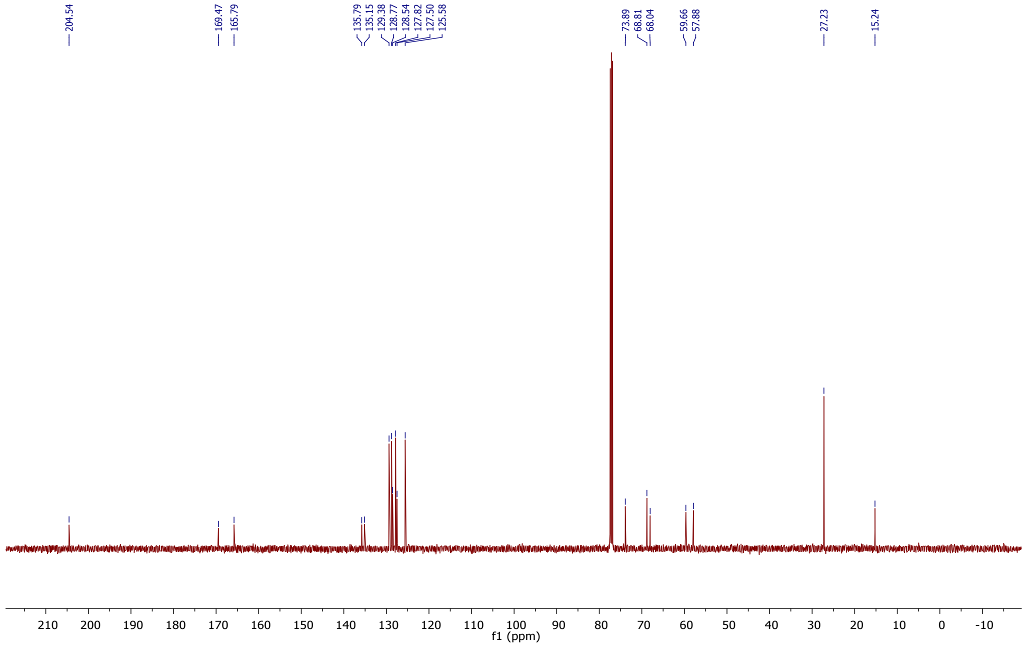
204.54
169.47
165.79
135.79
135.15
129.38
128.77
128.54
127.82
127.50
125.58
73.89
68.81
68.04
59.66
57.88
27.23
15.24
f1 (ppm)

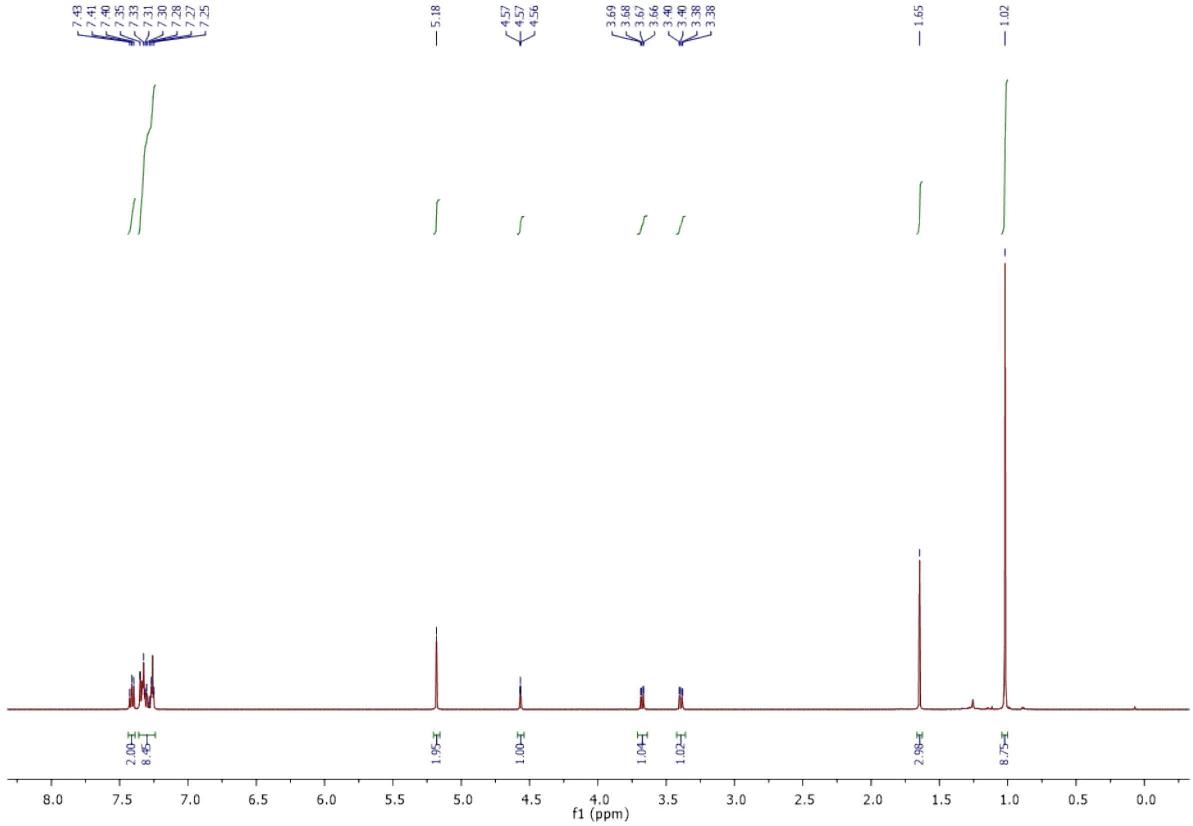
7.43
7.41
7.40
7.35
7.33
7.31
7.30
7.28
7.27
7.25
5.18
4.57
4.57
4.56
3.69
3.68
3.67
3.66
3.40
3.40
3.38
3.38
1.65
1.02
2.00
8.45
1.95
1.00
1.04
1.02
2.98
8.75
f1 (ppm)

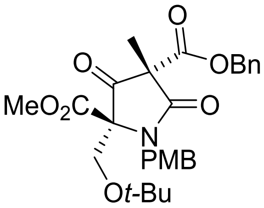

O
OBn
O
MeO2C
O
N
PMB
Ot-Bu

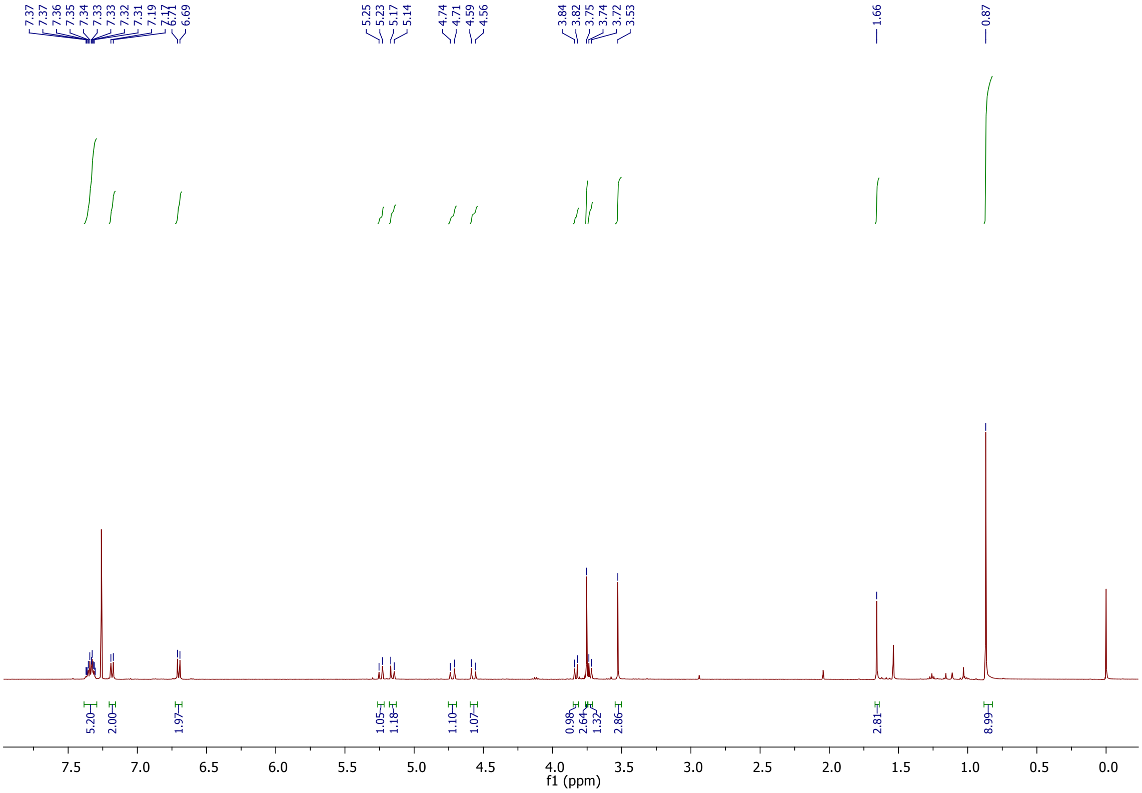

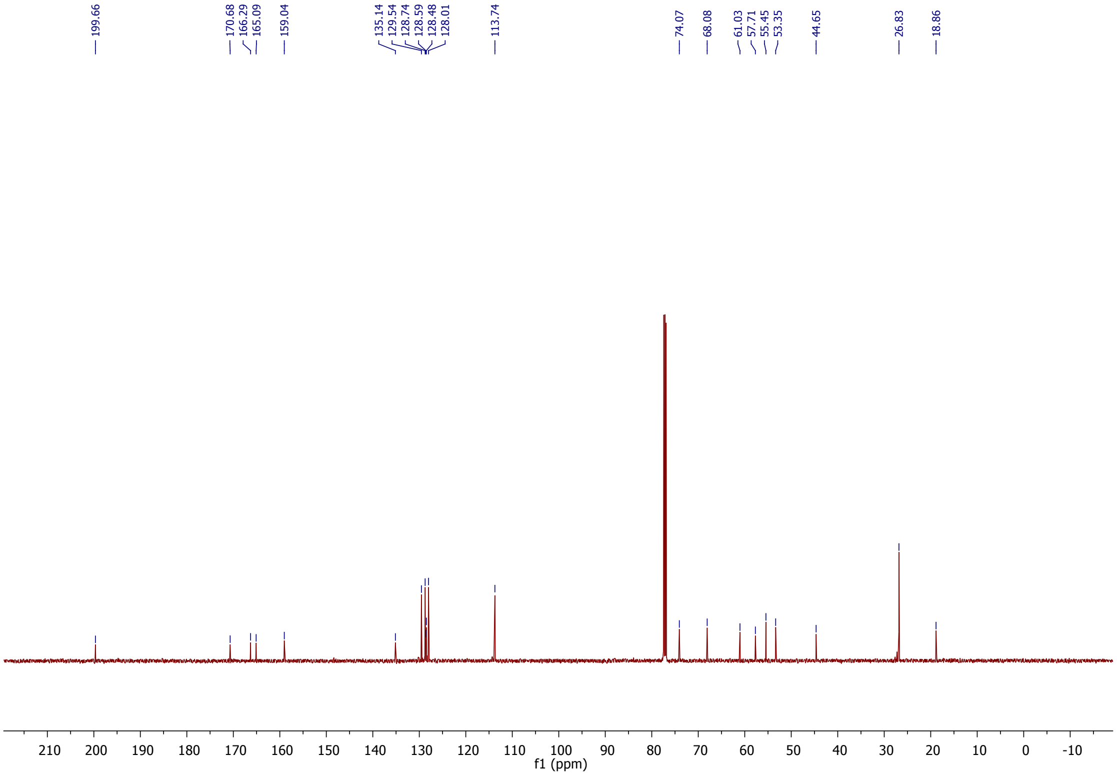

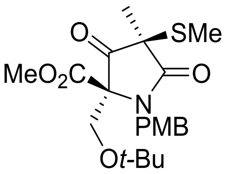
SMe
MeO2C
O
N
PMB
Ot-Bu

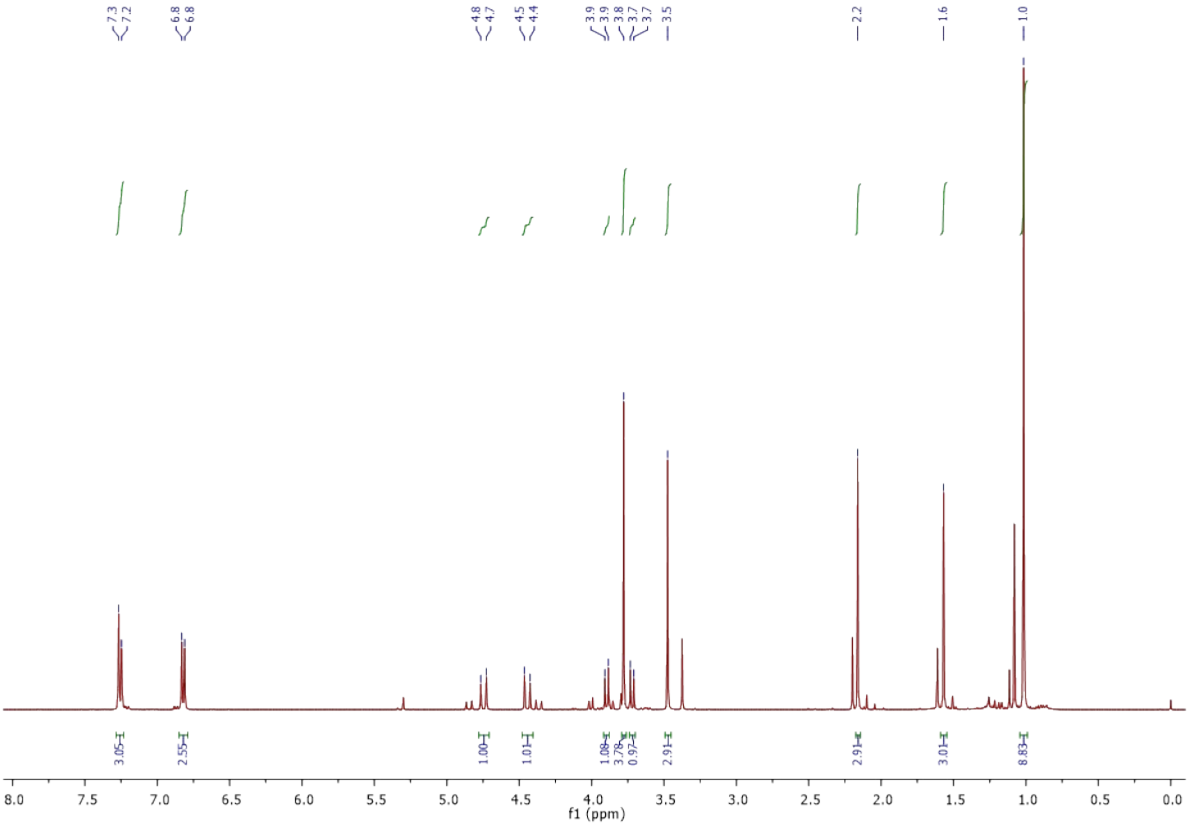

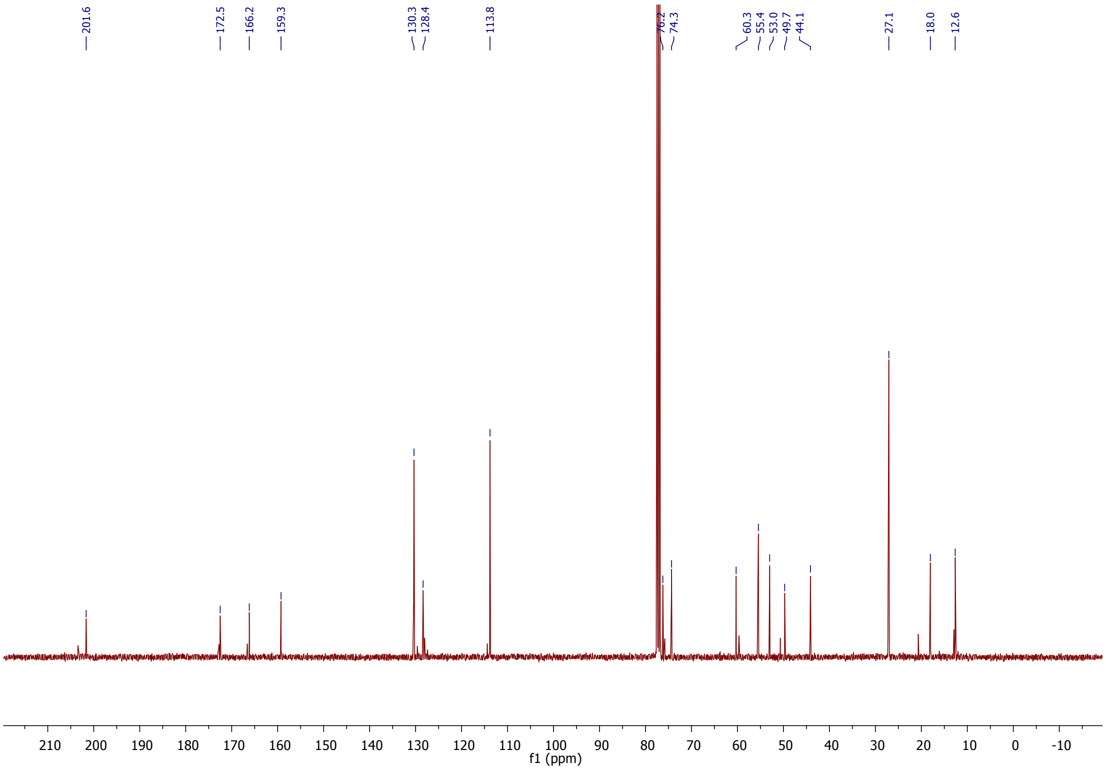

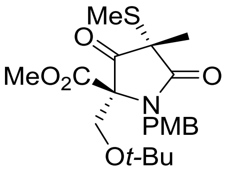
MeS
O
MeO2C
O
N
PMB
Ot-Bu

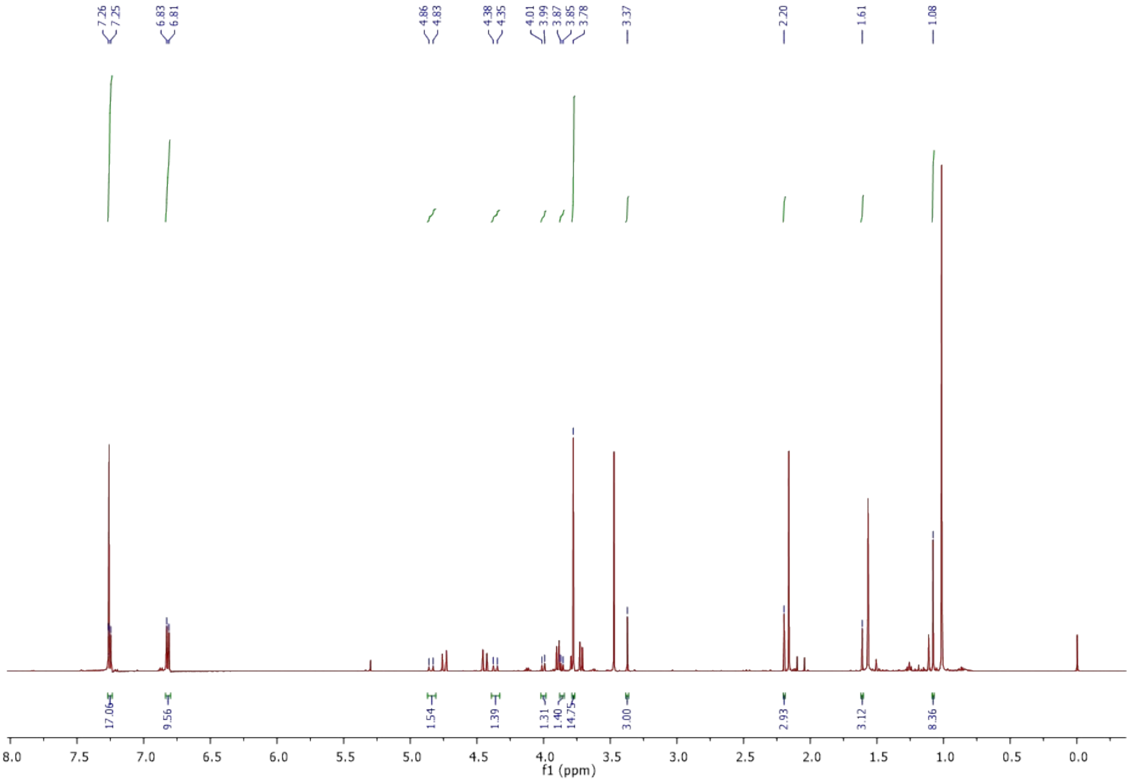
7.26
7.25
6.83
6.81
4.86
4.83
4.38
4.35
4.01
3.99
3.87
3.85
3.78
3.37
2.20
1.61
1.08
17.06
9.56
1.54
1.39
1.31
1.40
14.75
3.00
2.93
3.12
8.36
f1 (ppm)

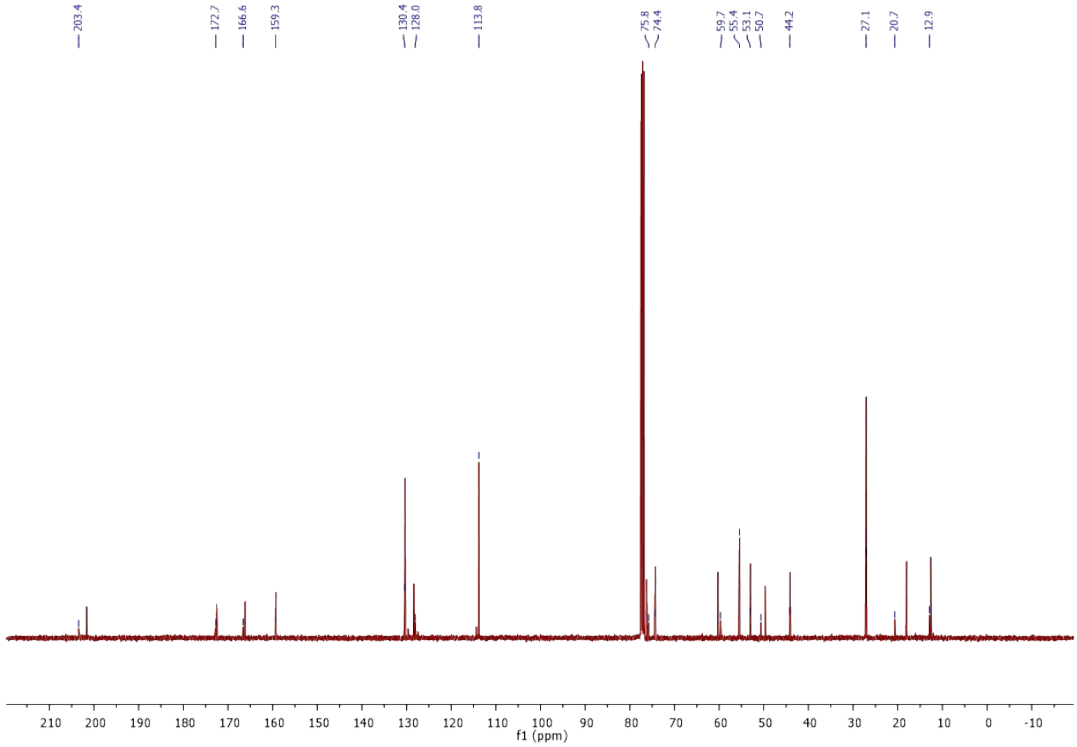
203.4
172.7
166.6
159.3
130.4
128.0
113.8
75.8
74.4
59.7
55.4
53.1
50.7
44.2
27.1
20.7
12.9
f1 (ppm)

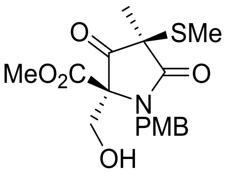
MeO2C
SMe
O
O
N
PMB
OH

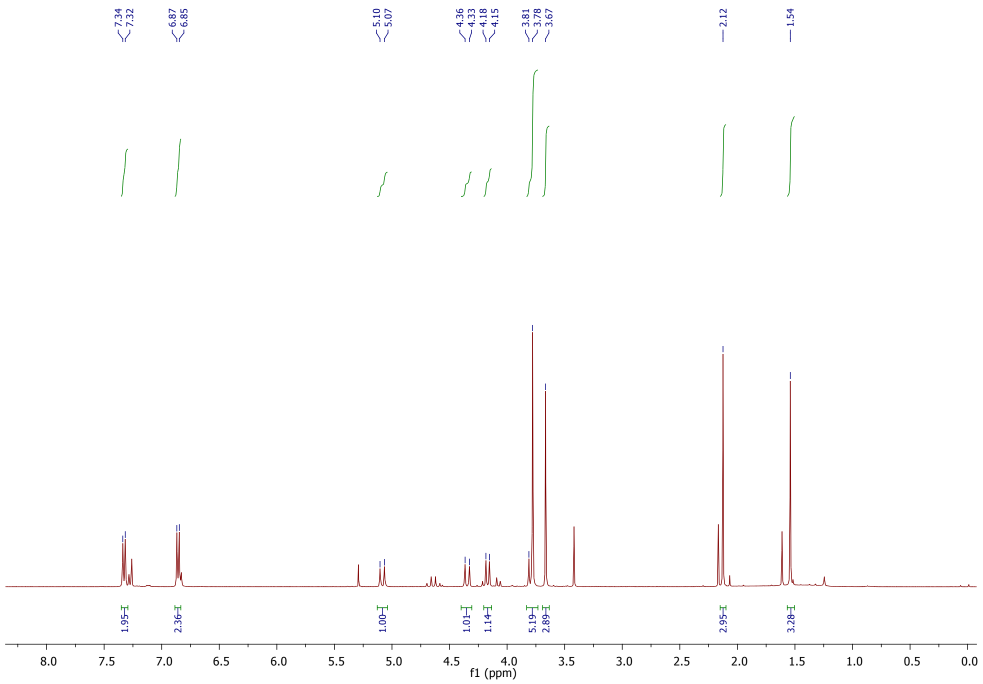
7.34
7.32
6.87
6.85
5.10
5.07
4.36
4.33
4.18
4.15
3.81
3.78
3.67
2.12
1.54
1.95
2.36
1.00
1.01
1.14
5.19
2.89
2.95
3.28
8.0
7.5
7.0
6.5
6.0
5.5
5.0
4.5
4.0
3.5
3.0
2.5
2.0
1.5
1.0
0.5
0.0
f1 (ppm)

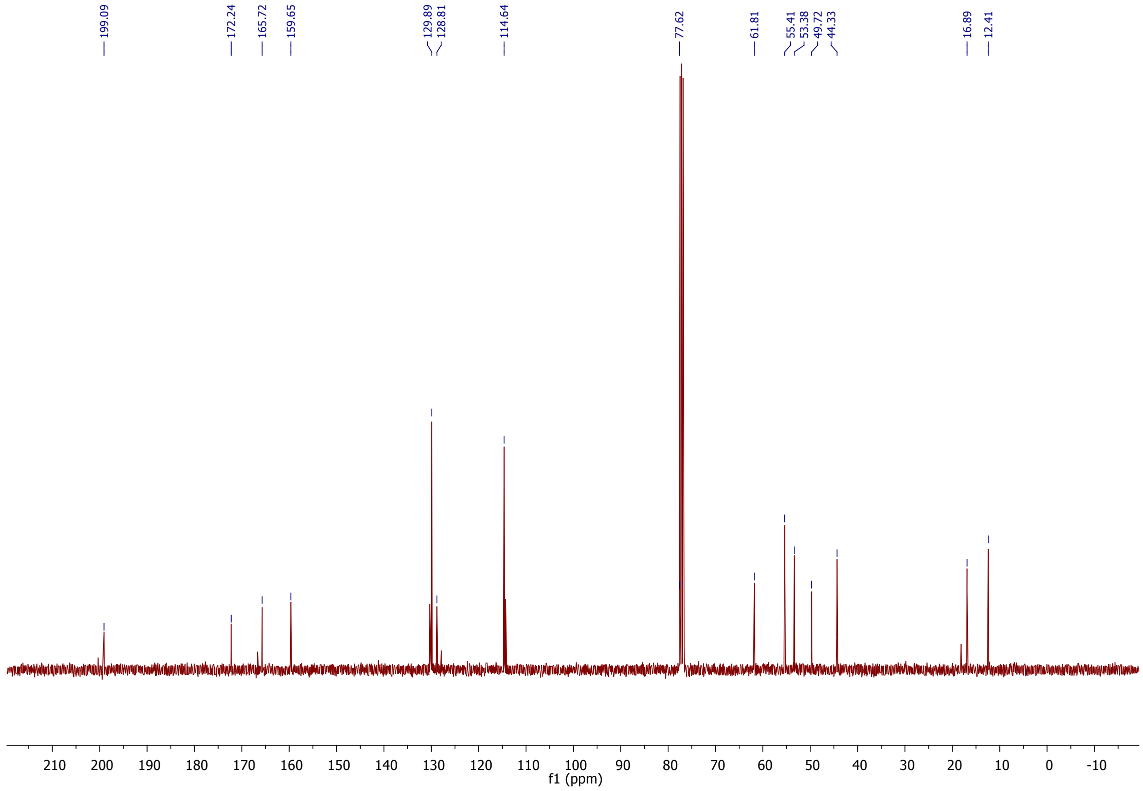
199.09
172.24
165.72
159.65
129.89
128.81
114.64
77.62
61.81
55.41
53.38
49.72
44.33
16.89
12.41
210
200
190
180
170
160
150
140
130
120
110
100
90
80
70
60
50
40
30
20
10
0
-10
f1 (ppm)

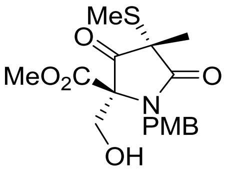

MeS
O
MeO2C
O
N
PMB
OH

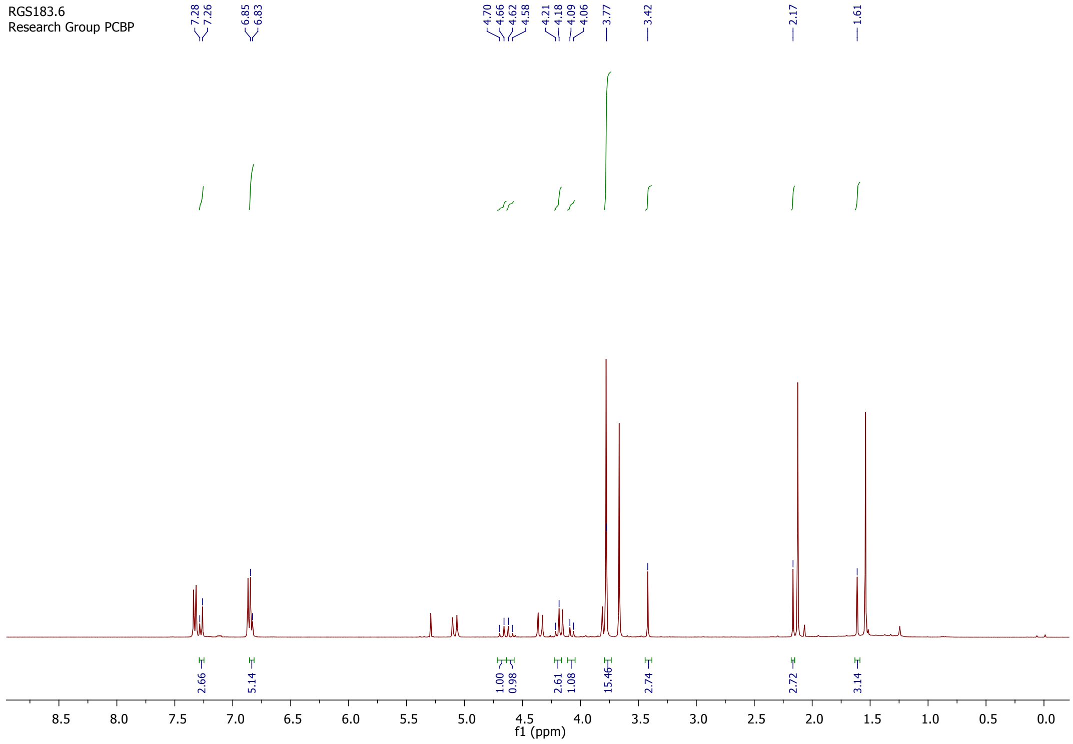

RGS183.6
Research Group PCBP
7.28
7.26
6.85
6.83
4.70
4.66
4.62
4.58
4.21
4.18
4.09
4.06
3.77
3.42
2.17
1.61
2.66
5.14
1.00
0.98
2.61
1.08
15.46
2.74
2.72
3.14
8.5 8.0 7.5 7.0 6.5 6.0 5.5 5.0 4.5 4.0 3.5 3.0 2.5 2.0 1.5 1.0 0.5 0.0
f1 (ppm)

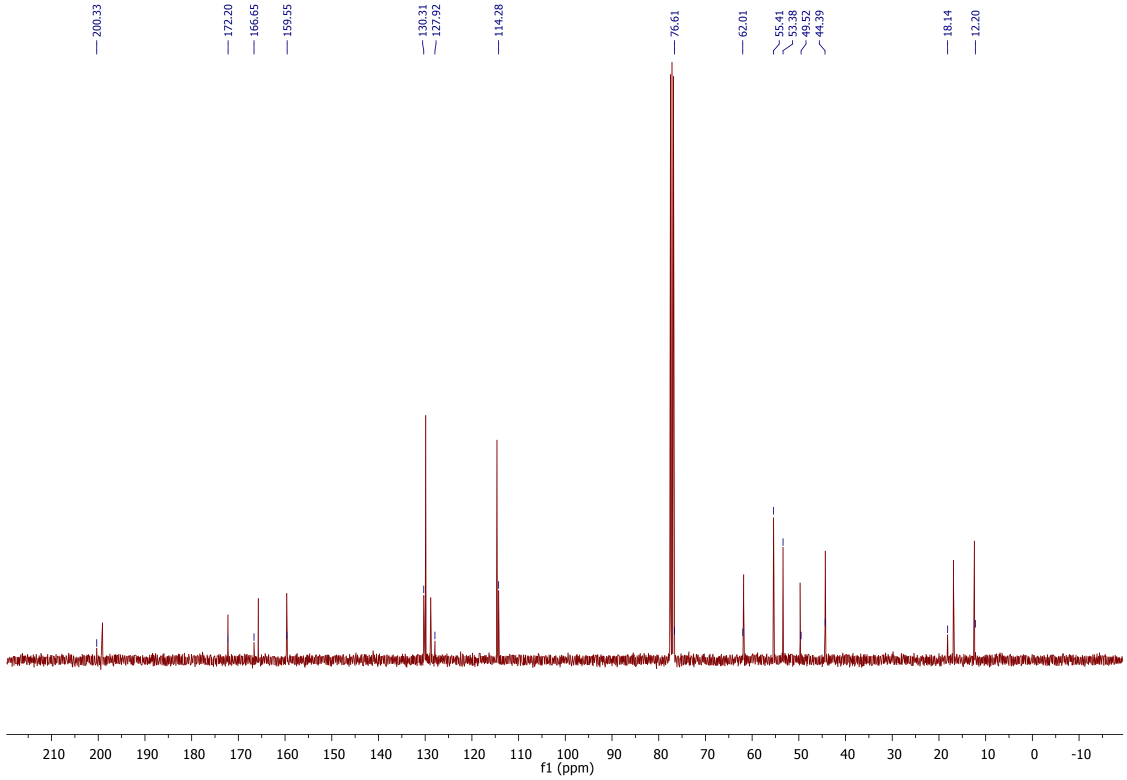

200.33
172.20
166.65
159.55
130.31
127.92
114.28
76.61
62.01
55.41
53.38
49.52
44.39
18.14
12.20
210 200 190 180 170 160 150 140 130 120 110 100 90 80 70 60 50 40 30 20 10 0 -10
f1 (ppm)

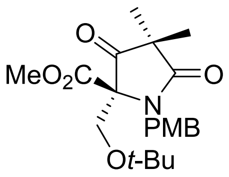
O
MeO2C
N
O
PMB
Ot-Bu

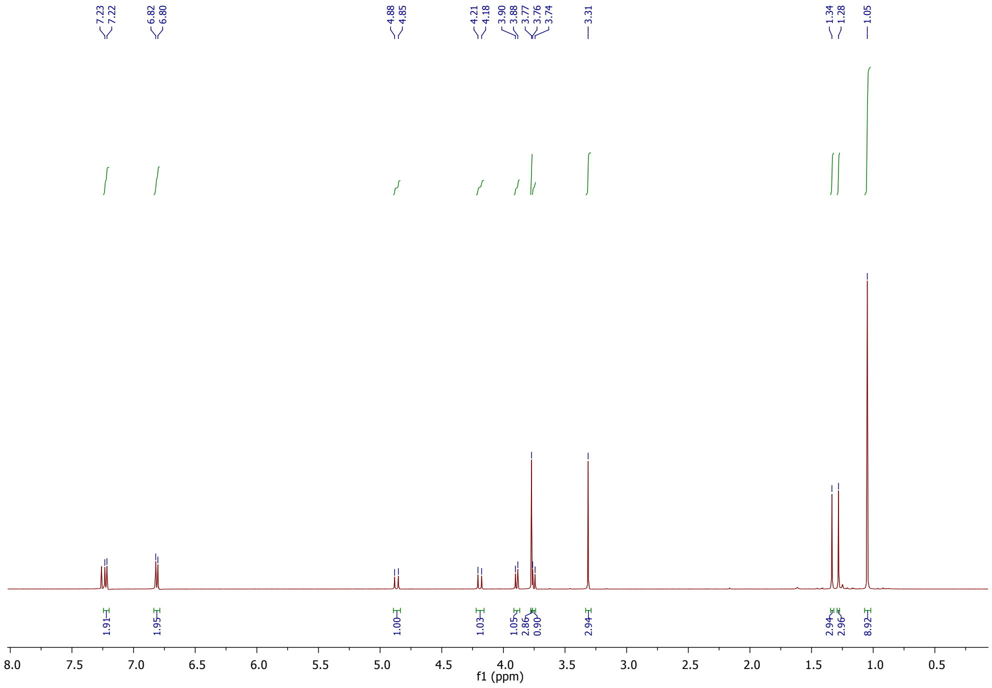
7.23
7.22
6.82
6.80
4.88
4.85
4.21
4.18
3.90
3.88
3.77
3.76
3.74
3.31
1.34
1.28
1.05
1.91
1.95
1.00
1.03
1.05
2.86
0.90
2.94
2.94
2.96
8.92
f1 (ppm)

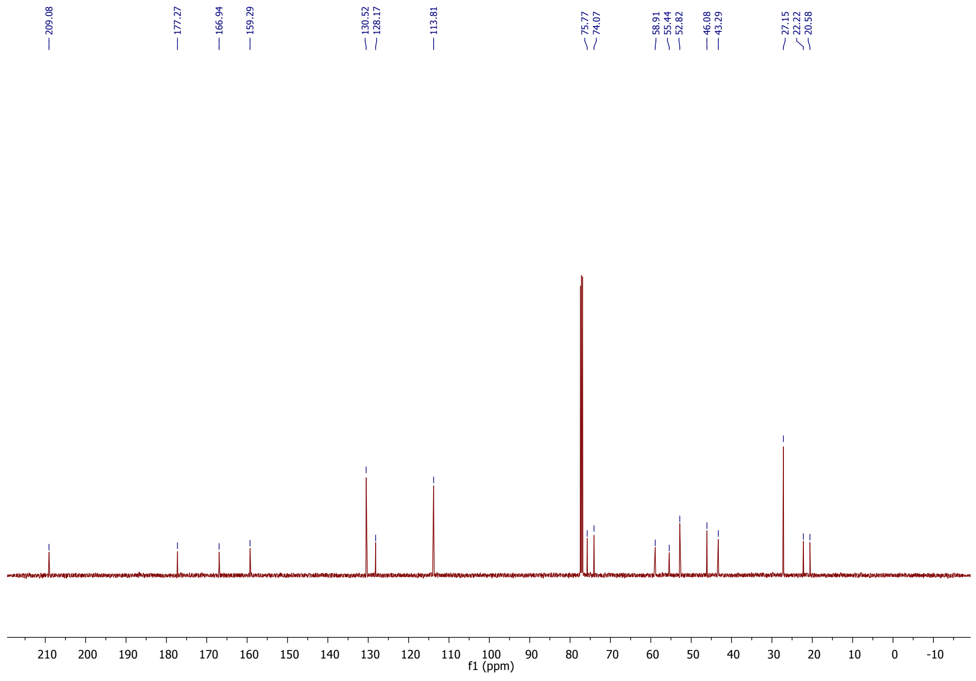
209.08
177.27
166.94
159.29
130.52
128.17
113.81
75.77
74.07
58.91
55.44
52.82
46.08
43.29
27.15
22.22
20.58
f1 (ppm)

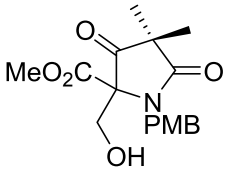
O
MeO2C
O
N
PMB
OH

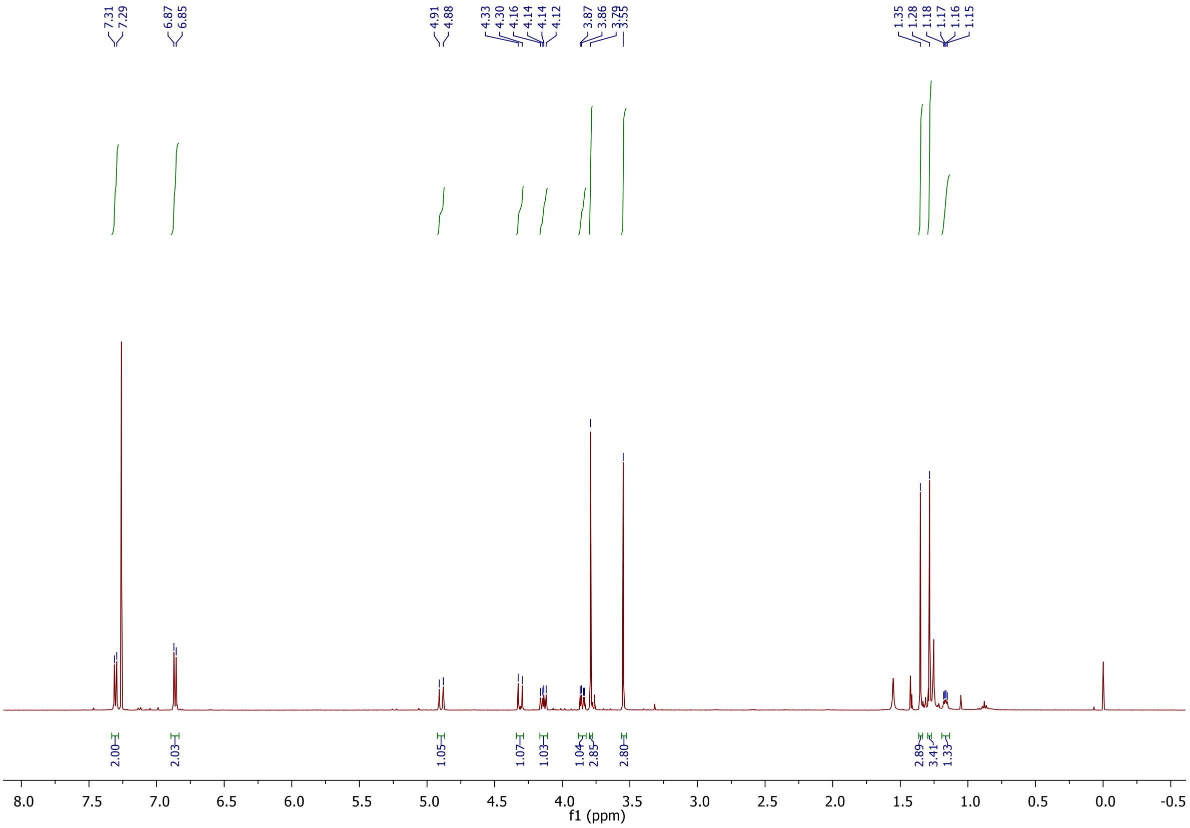
7.31
7.29
6.87
6.85
4.91
4.88
4.33
4.30
4.16
4.14
4.14
4.12
3.87
3.86
3.79
3.55
1.35
1.28
1.18
1.17
1.16
1.15
2.00
2.03
1.05
1.07
1.03
1.04
2.85
2.80
2.89
3.41
1.33
8.0 7.5 7.0 6.5 6.0 5.5 5.0 4.5 4.0 3.5 3.0 2.5 2.0 1.5 1.0 0.5 0.0 -0.5
f1 (ppm)

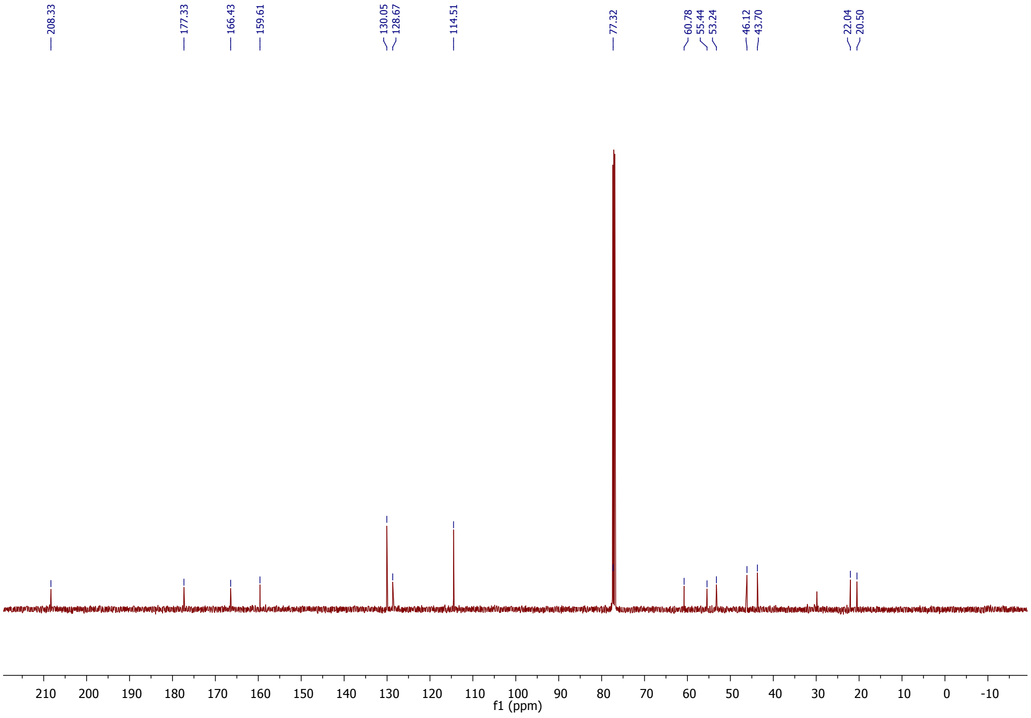
208.33
177.33
166.43
159.61
130.05
128.67
114.51
77.32
60.78
55.44
53.24
46.12
43.70
22.04
20.50
210 200 190 180 170 160 150 140 130 120 110 100 90 80 70 60 50 40 30 20 10 0 -10
f1 (ppm)

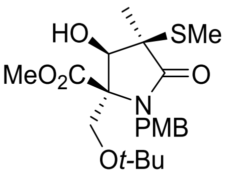
HO
SMe
MeO2C
O
N
PMB
Ot-Bu

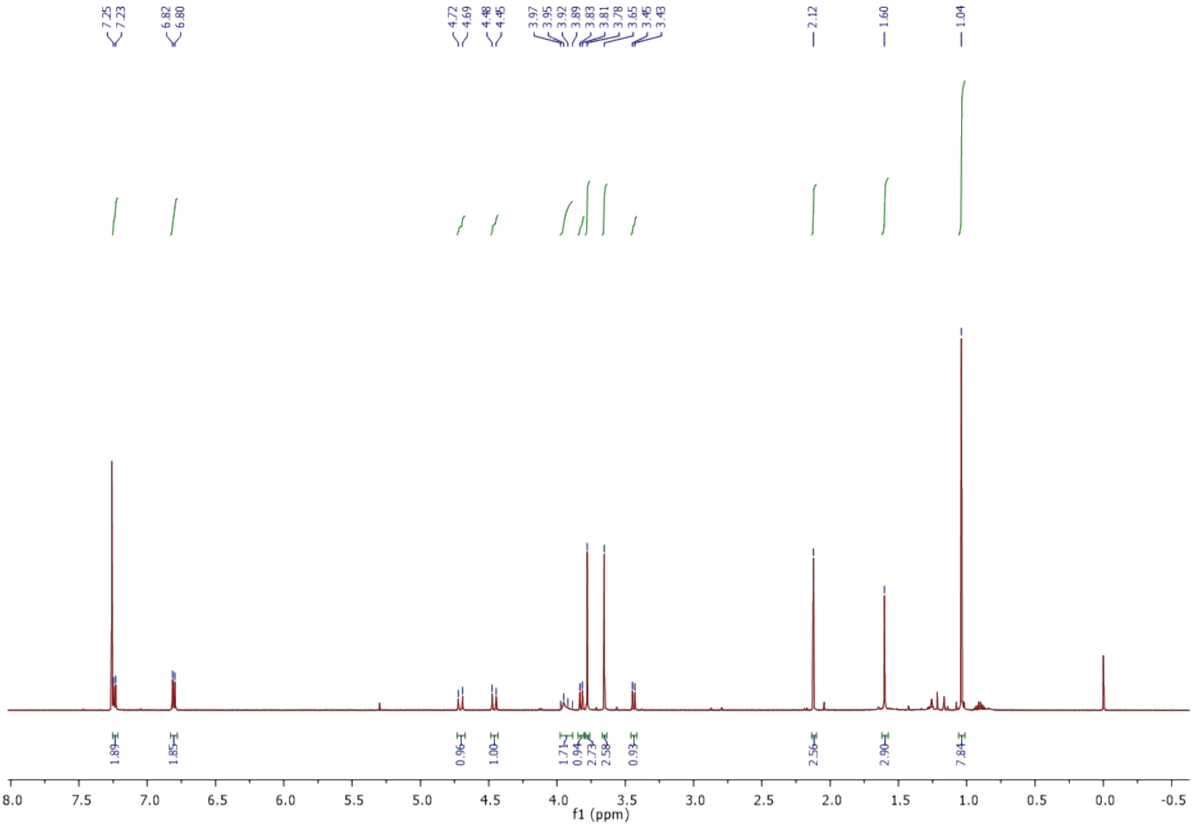
7.25
7.23
6.82
6.80
4.72
4.69
4.48
4.45
3.97
3.95
3.92
3.89
3.83
3.81
3.78
3.65
3.45
3.43
2.12
1.60
1.04
1.89
1.85
0.96
1.00
1.71
0.94
2.73
2.58
0.93
2.56
2.90
7.84
f1 (ppm)

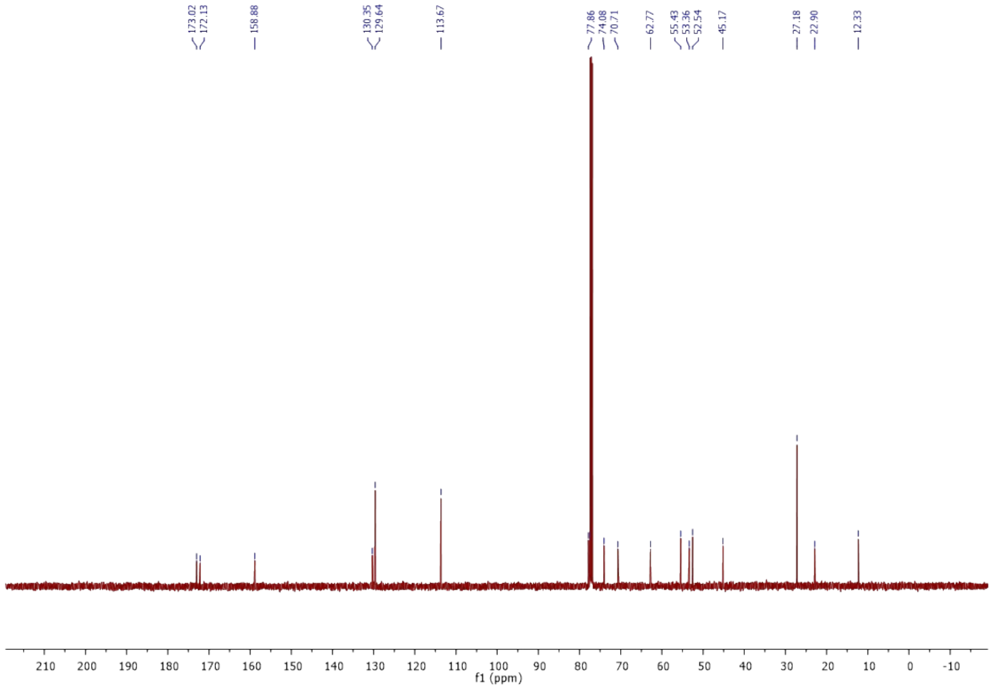
173.02
172.13
158.88
130.35
129.64
113.67
77.86
74.08
70.71
62.77
55.43
53.36
52.54
45.17
27.18
22.90
12.33
f1 (ppm)

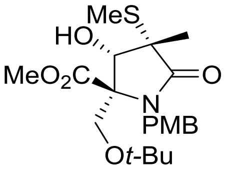
MeS
HO
MeO2C
O
N
PMB
Ot-Bu

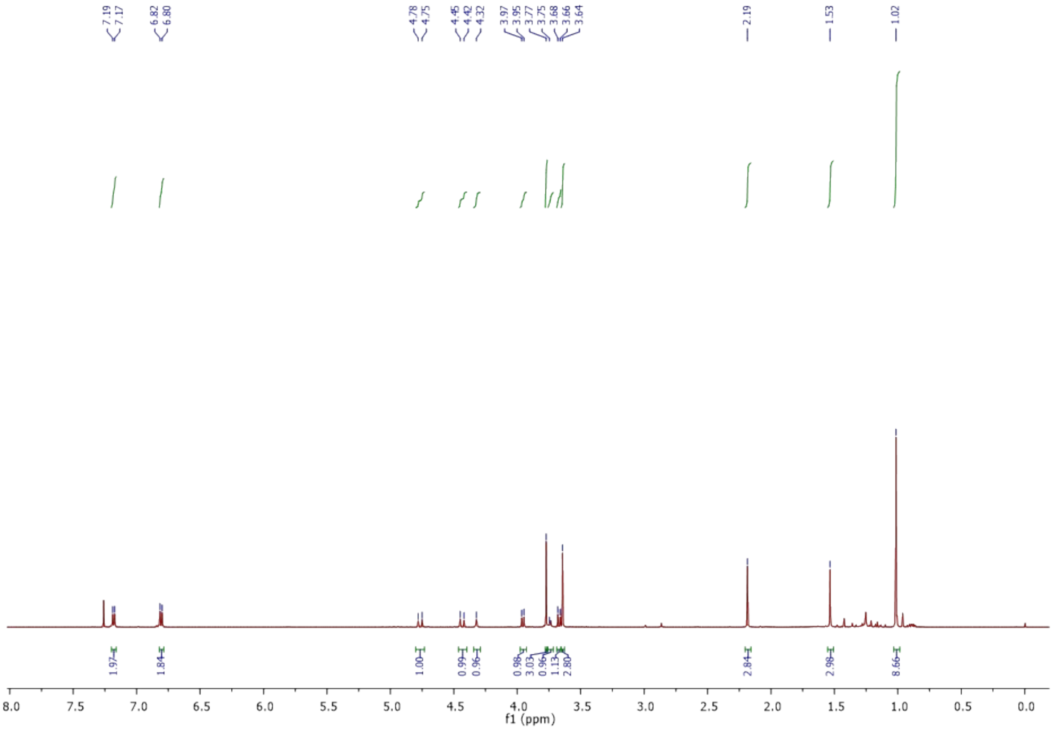
7.19
7.17
6.82
6.80
4.78
4.75
4.45
4.42
4.32
3.97
3.95
3.77
3.75
3.68
3.66
3.64
2.19
1.53
1.02
1.97
1.84
1.00
0.99
0.96
0.98
3.03
0.96
1.13
2.80
2.84
2.98
8.66
f1 (ppm)

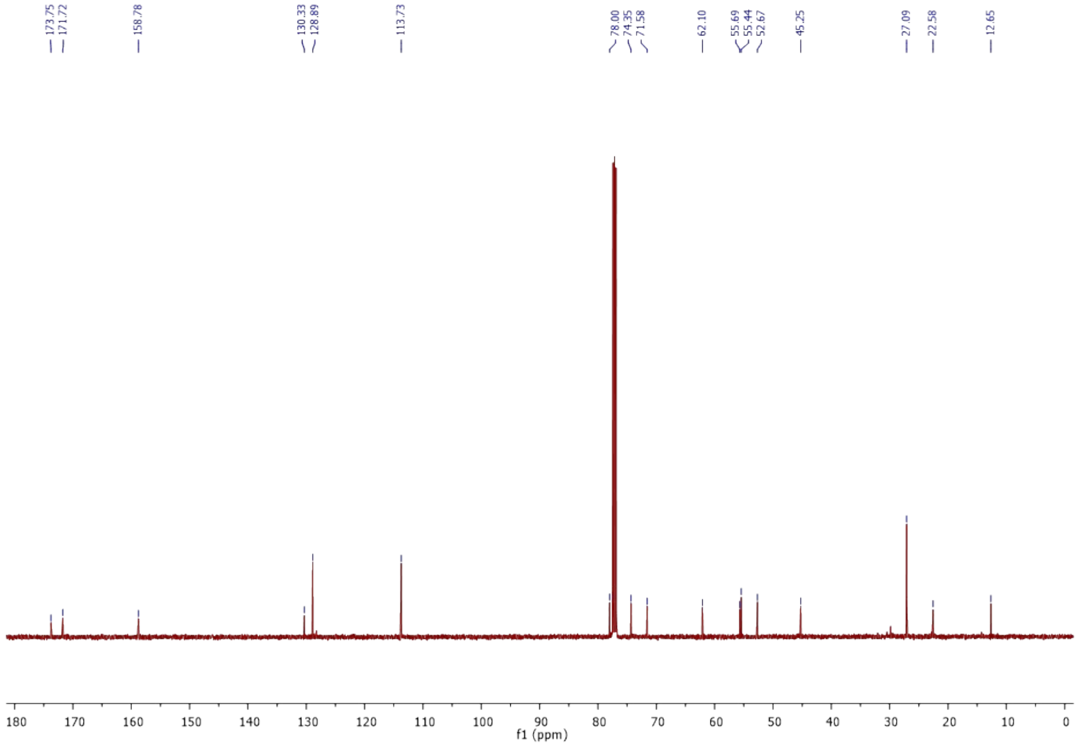
173.75
171.72
158.78
130.33
128.89
113.73
78.00
74.35
71.58
62.10
55.69
55.44
52.67
45.25
27.09
22.58
12.65
f1 (ppm)

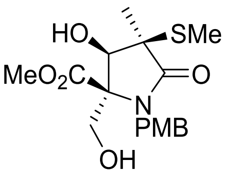

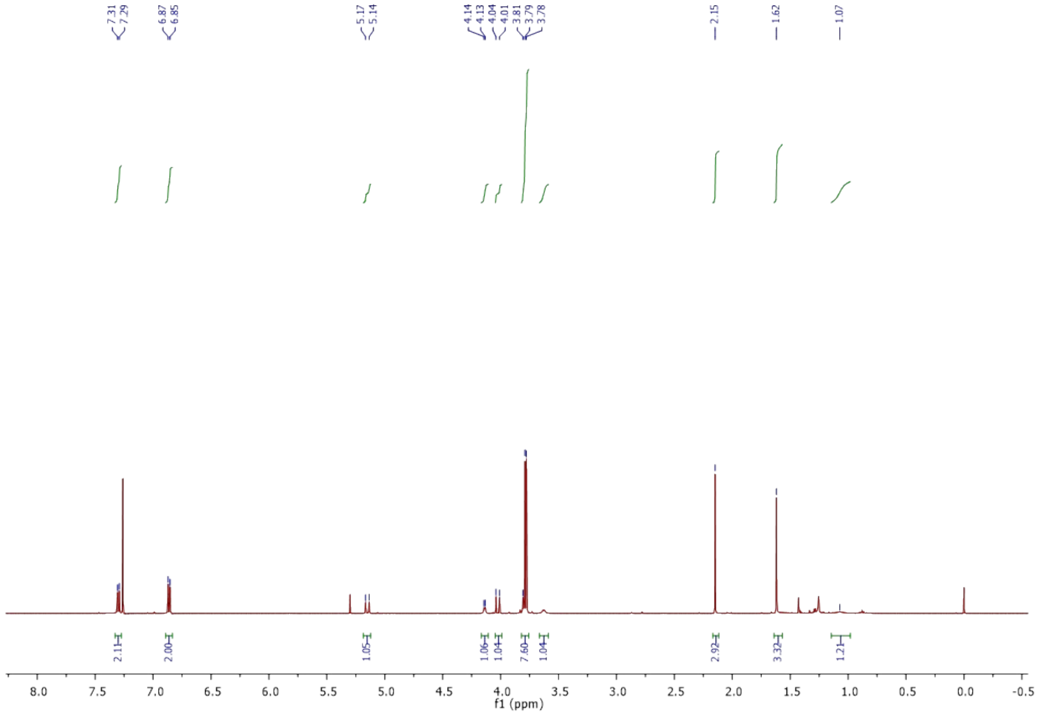

7.31
7.29
6.87
6.85
5.17
5.14
4.14
4.13
4.04
4.01
3.81
3.79
3.78
2.15
1.62
1.07
2.11
2.00
1.05
1.06
1.04
7.60
1.04
2.92
3.32
1.21
f1 (ppm)

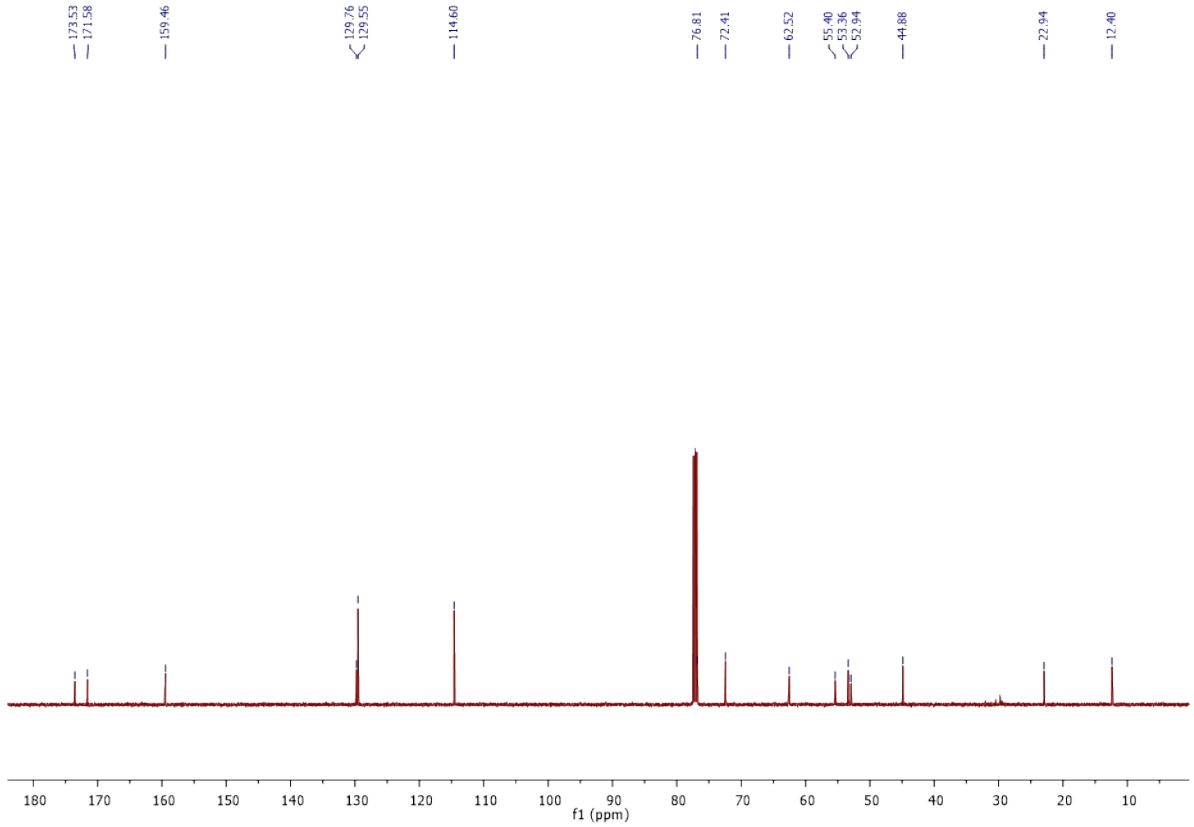

173.53
171.58
159.46
129.76
129.55
114.60
76.81
72.41
62.52
55.40
53.36
52.94
44.88
22.94
12.40
f1 (ppm)

HPLC traces

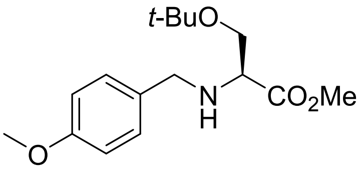

Racemic trace

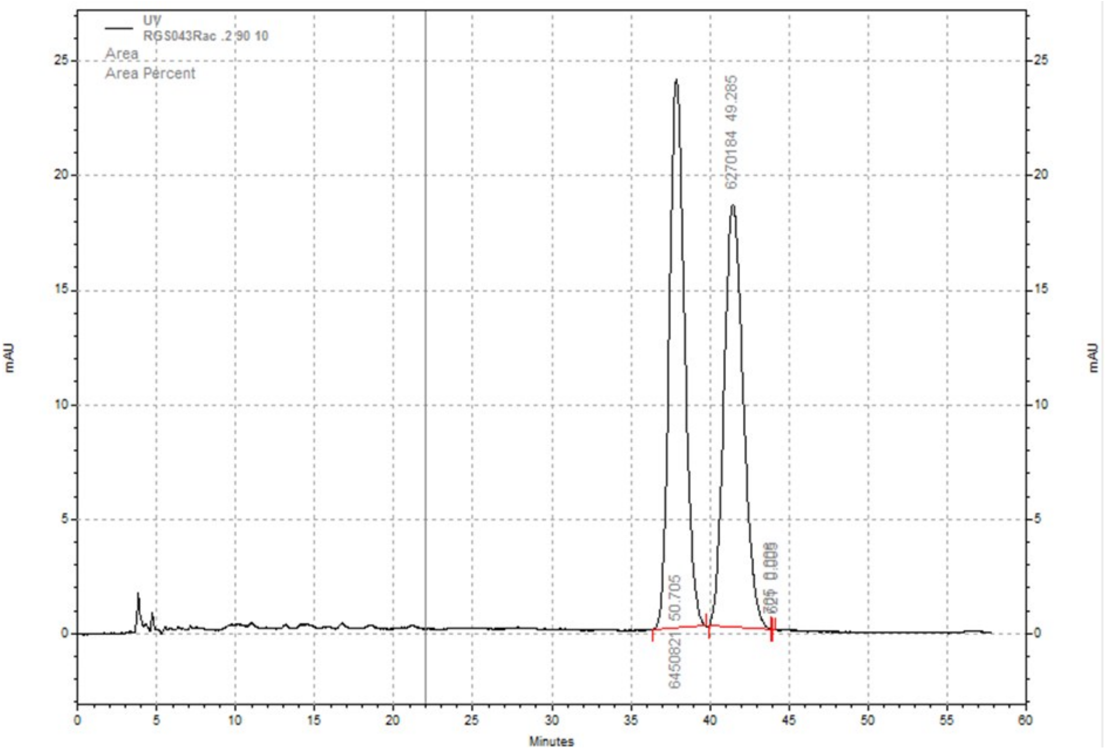

Non-racemic trace

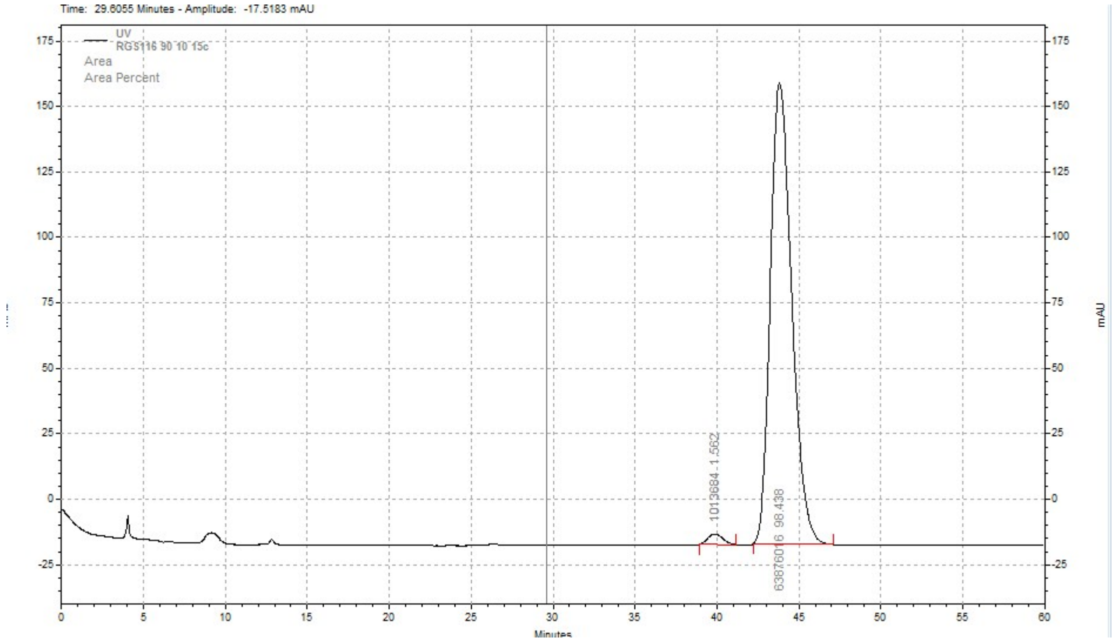

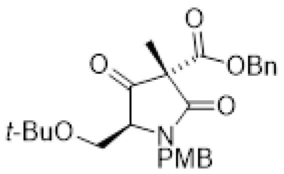

Racemic trace

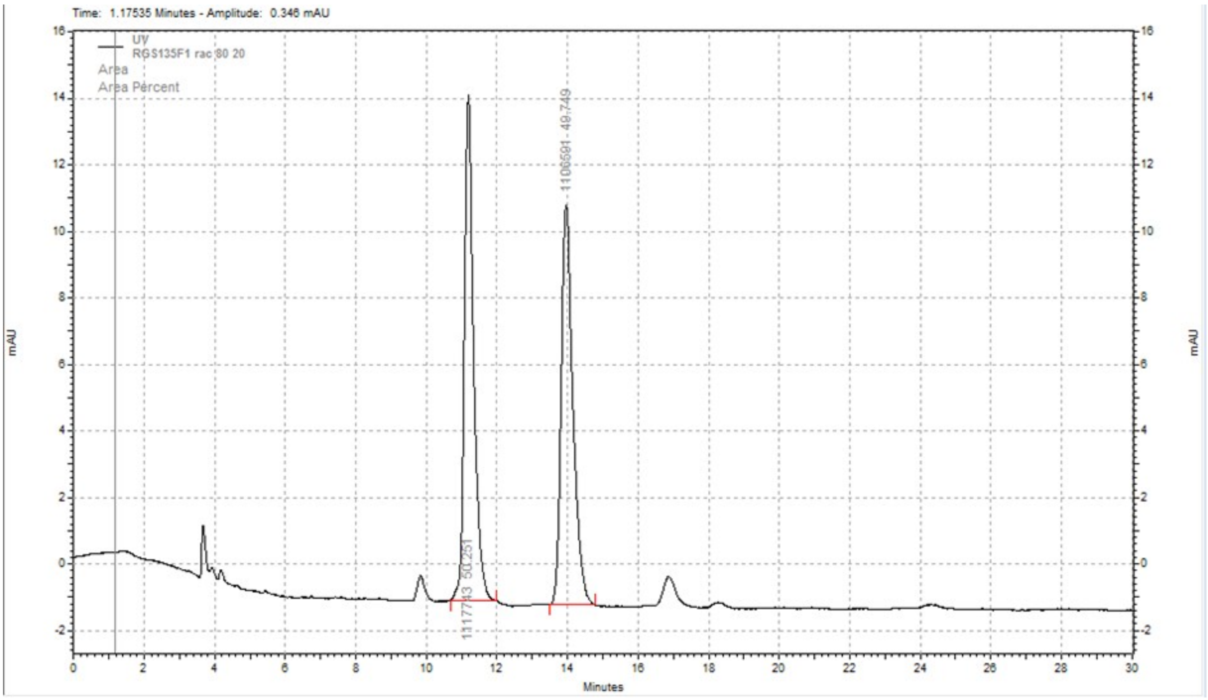

Non racemic trace

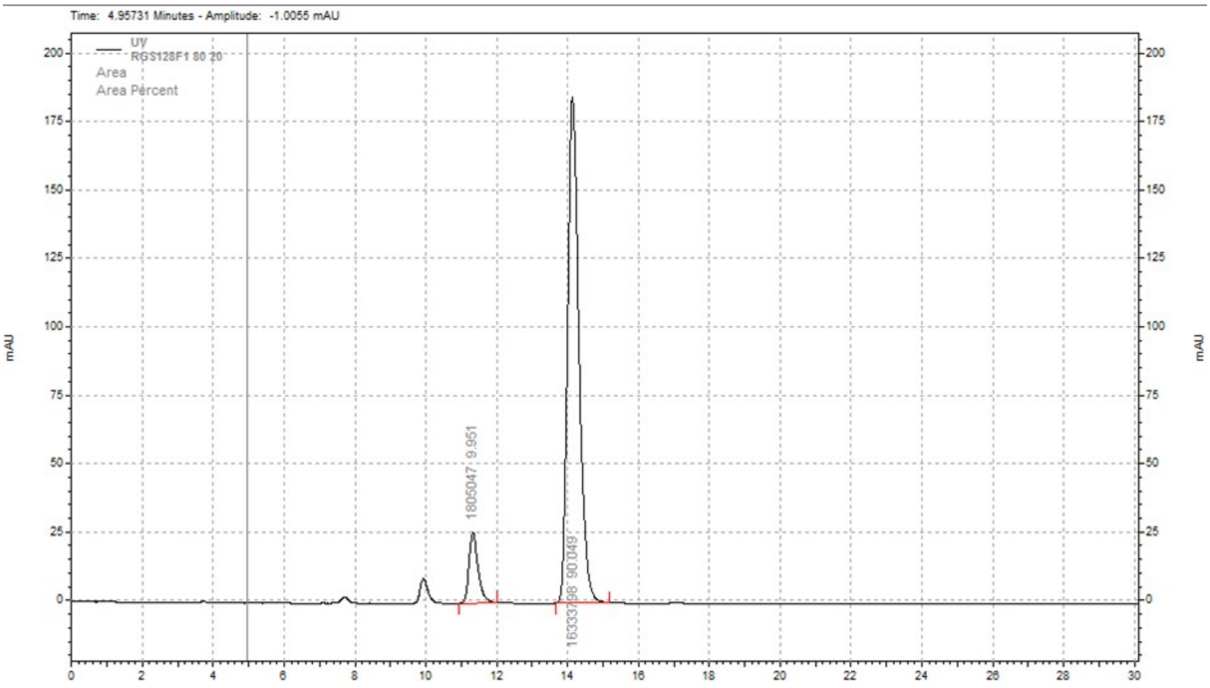

Trace of the crystals after a recrystallisation from IPA

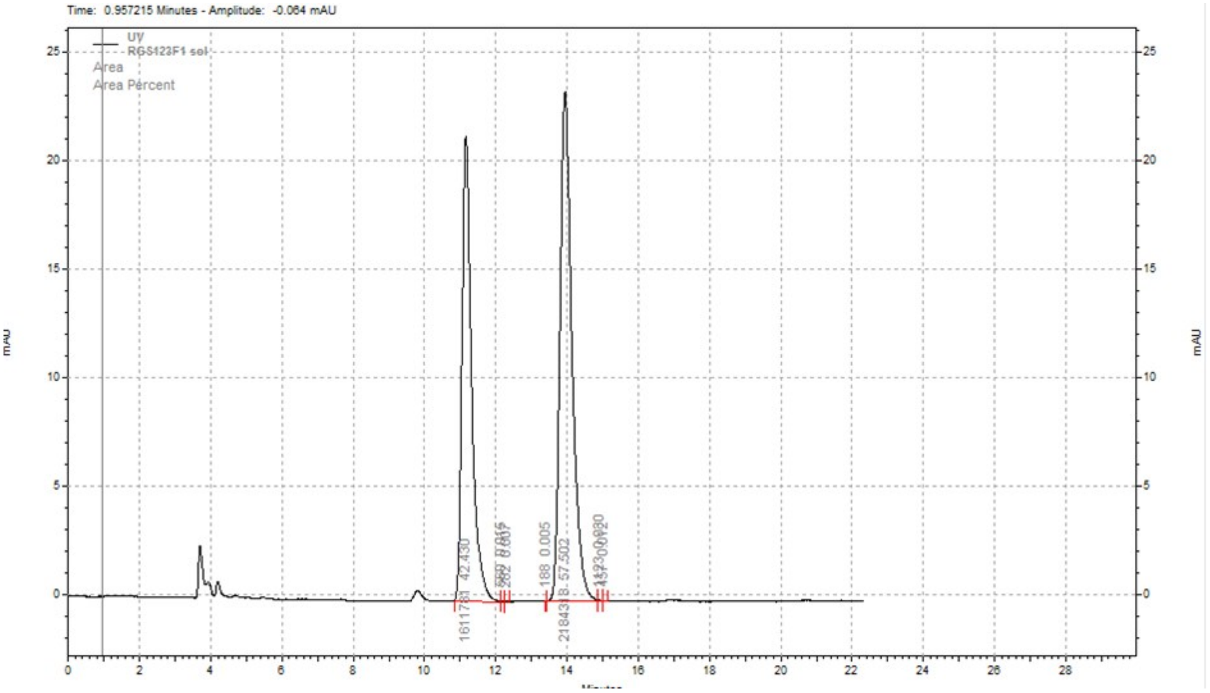

Trace of the supernatant after a recrystalisation from IPA

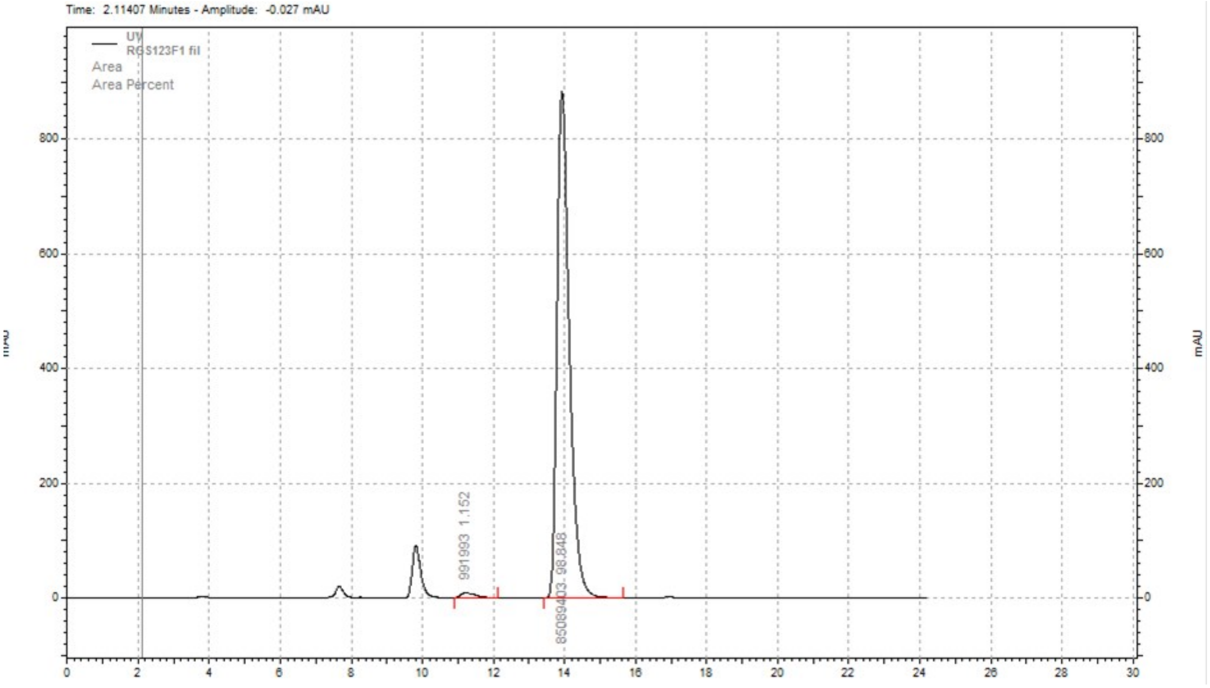

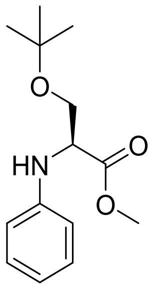

Racemic trace

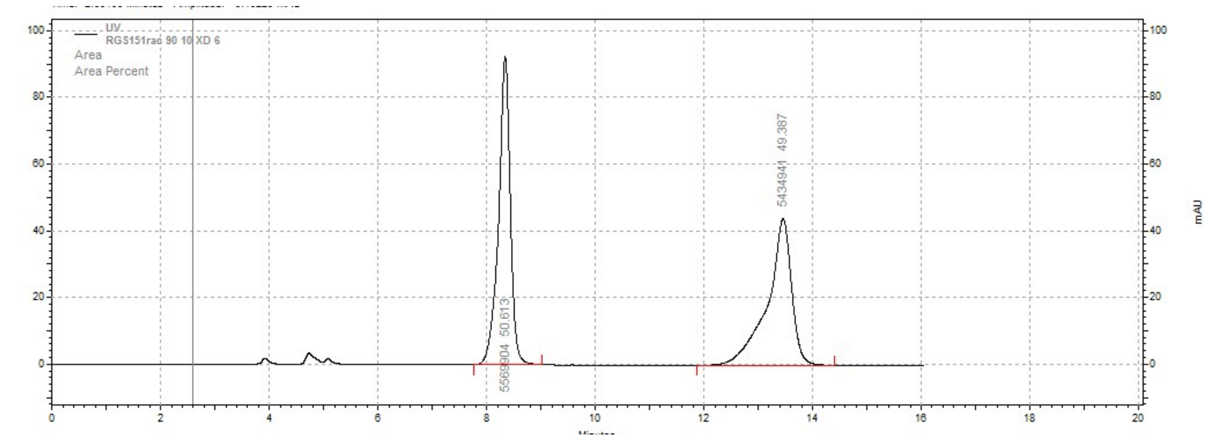

Non-racemic trace

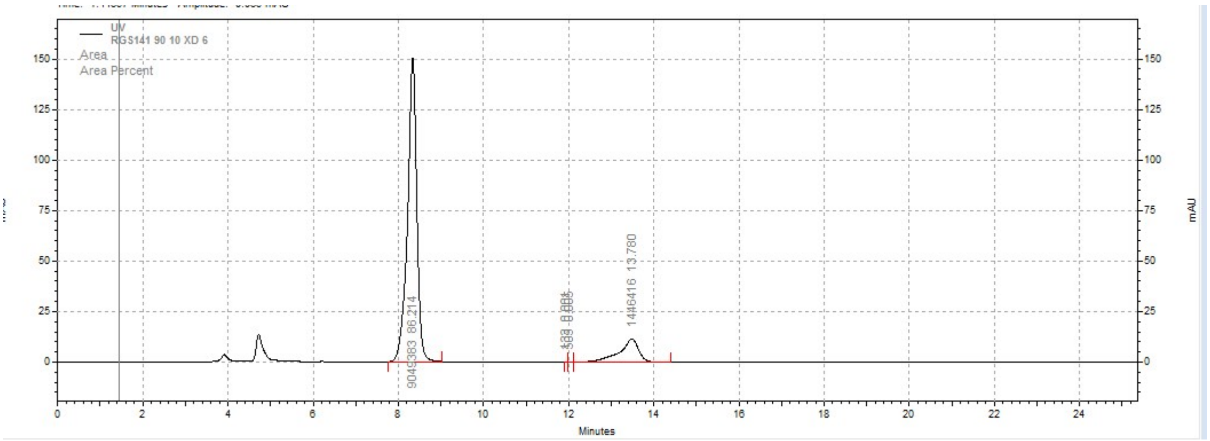

Trace of the crystals after a recrystallisation from petroleum ether (40-60 °C)

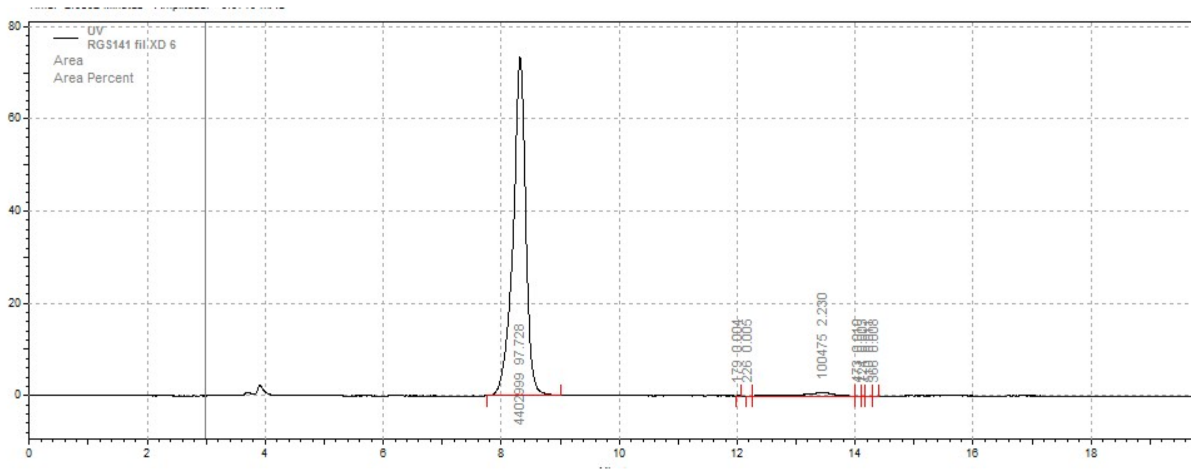

Trace of the supernatant after a recrystallisation from petroleum ether (40-60 °C)

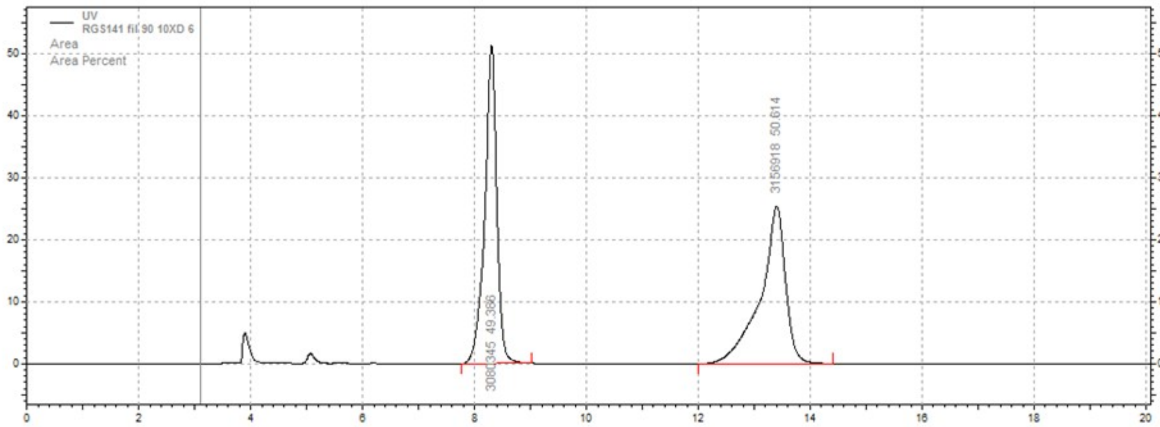

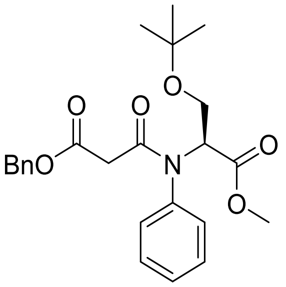

Racemic trace

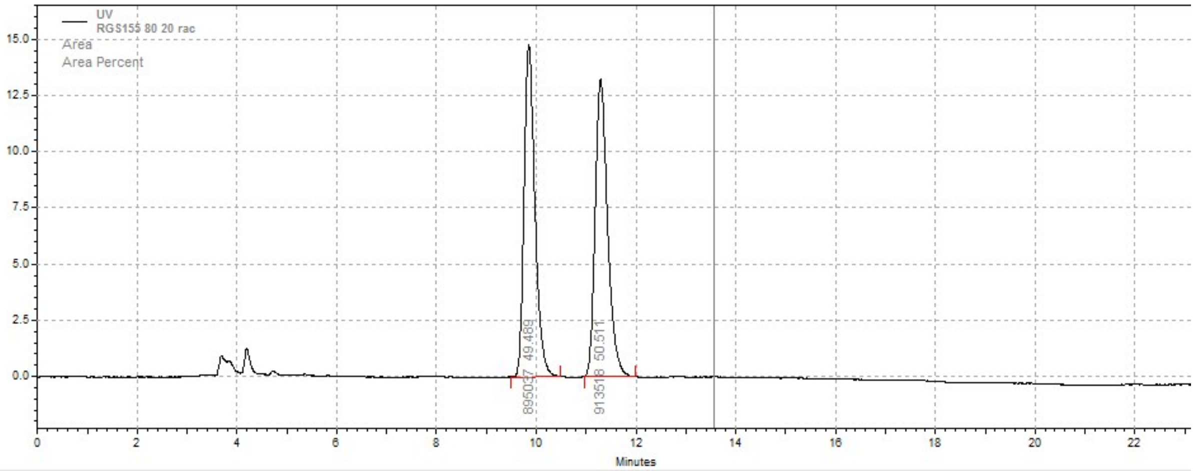

Non-racemic trace

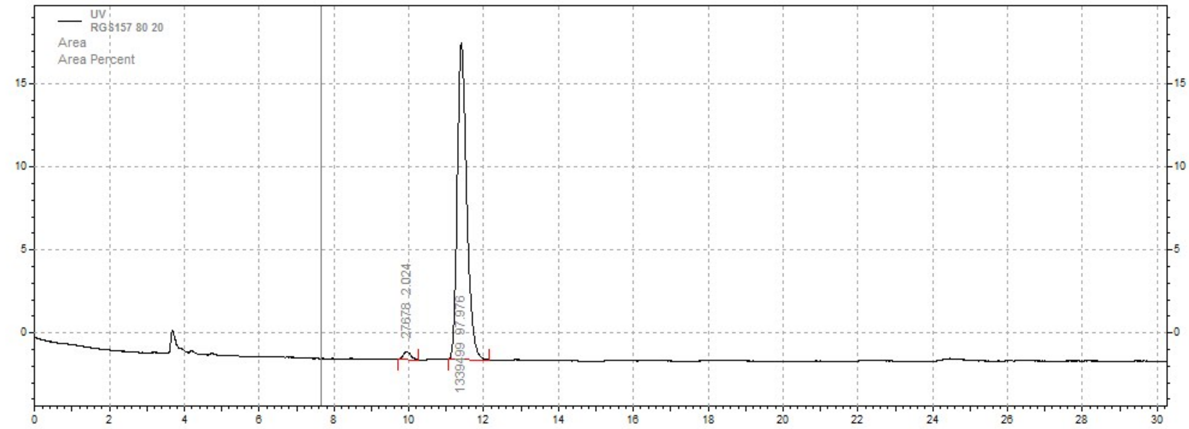

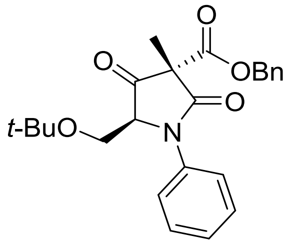

Racemic trace

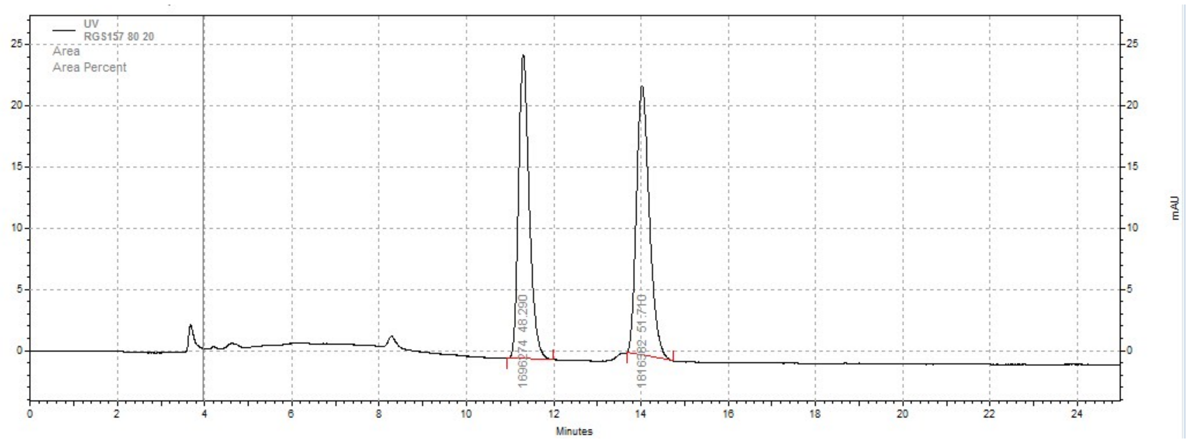

Non-racemic trace

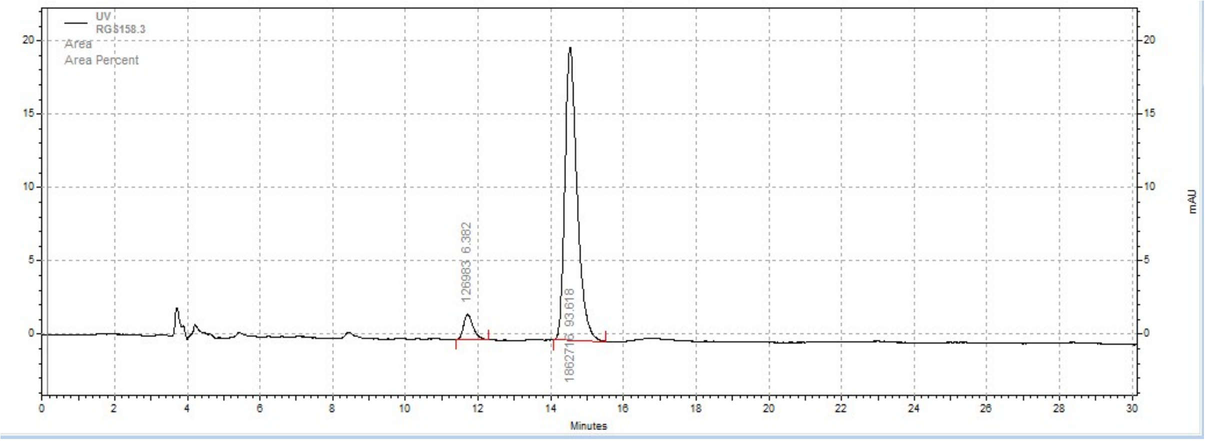

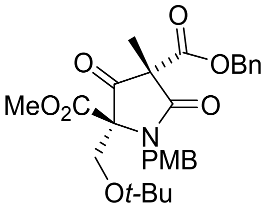

Racemic trace

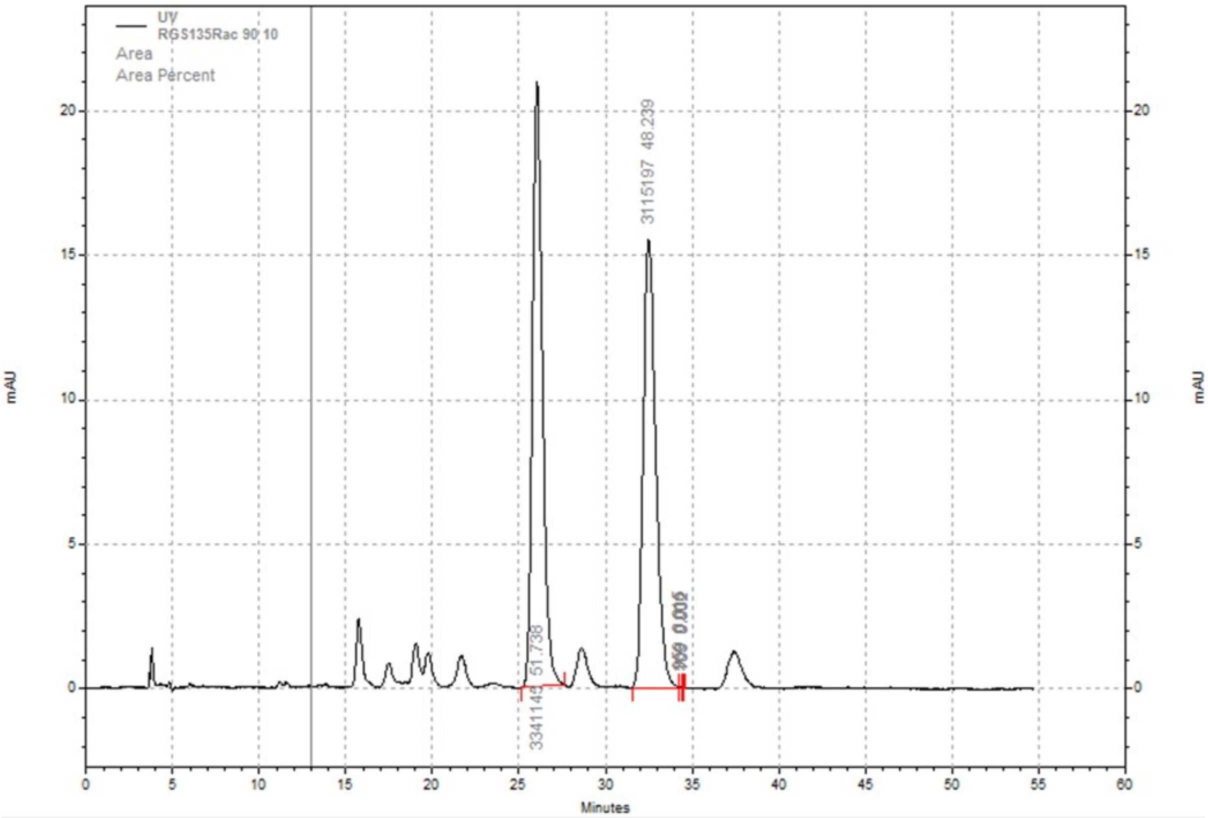

Non-racemic trace

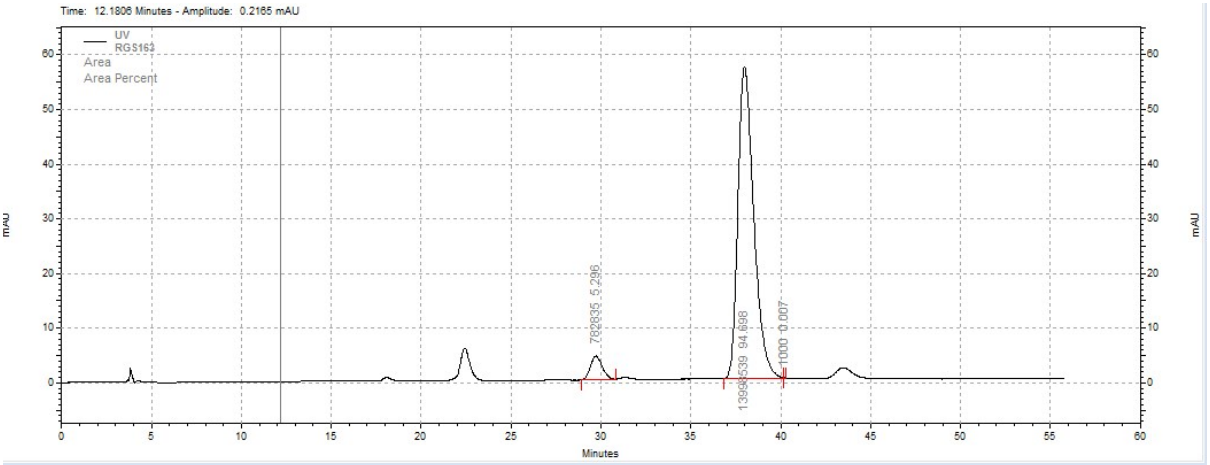

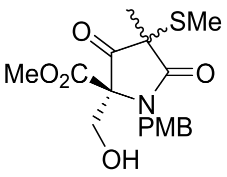

Racemic trace showing the major diastereomer

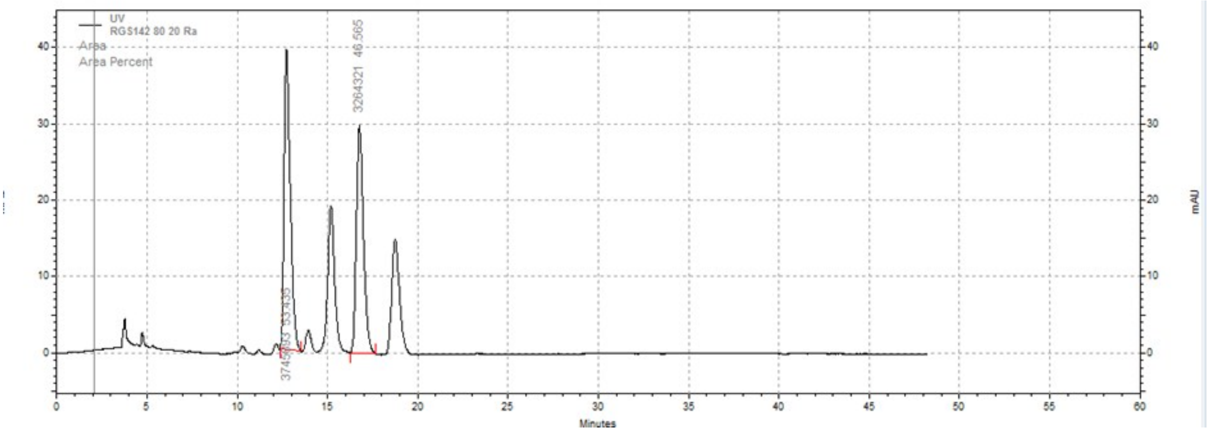

Non-racemic trace showing the major diastereomer

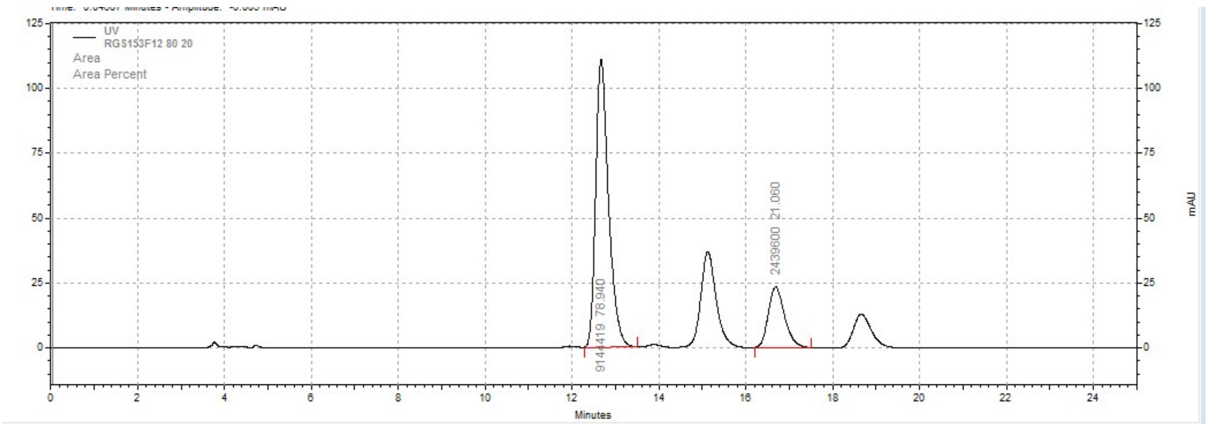

Racemic trace showing the major diastereomer

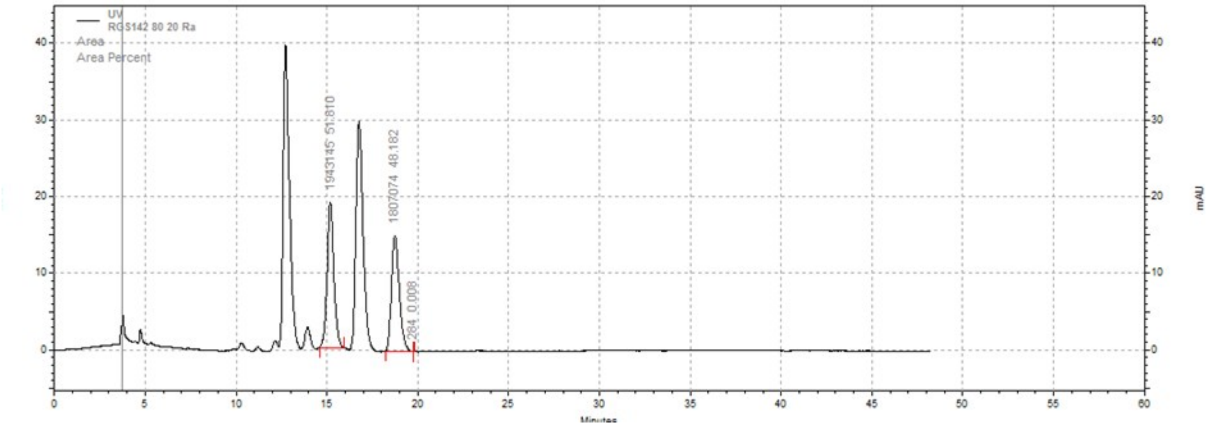

Racemic trace showing the minor diastereomer

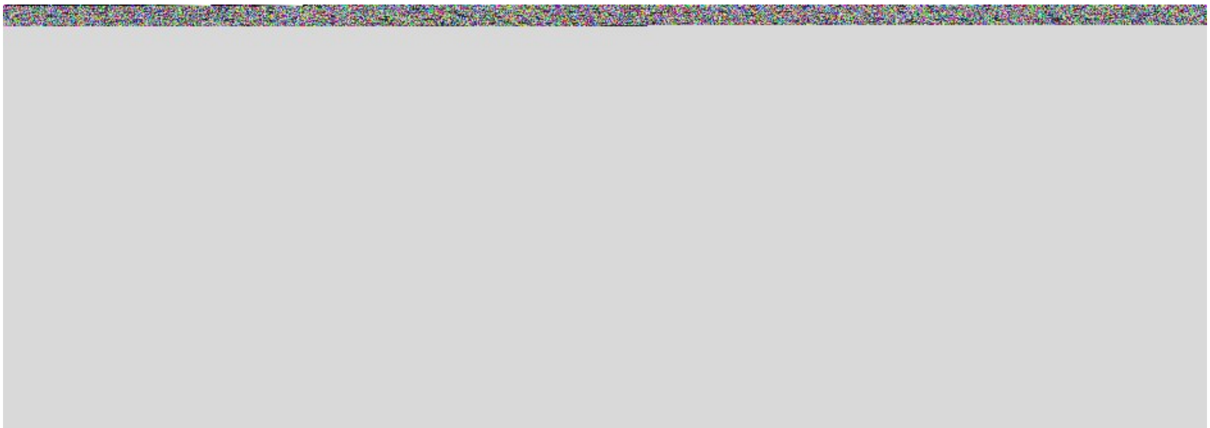

Non-racemic trace showing the minor diasteromer

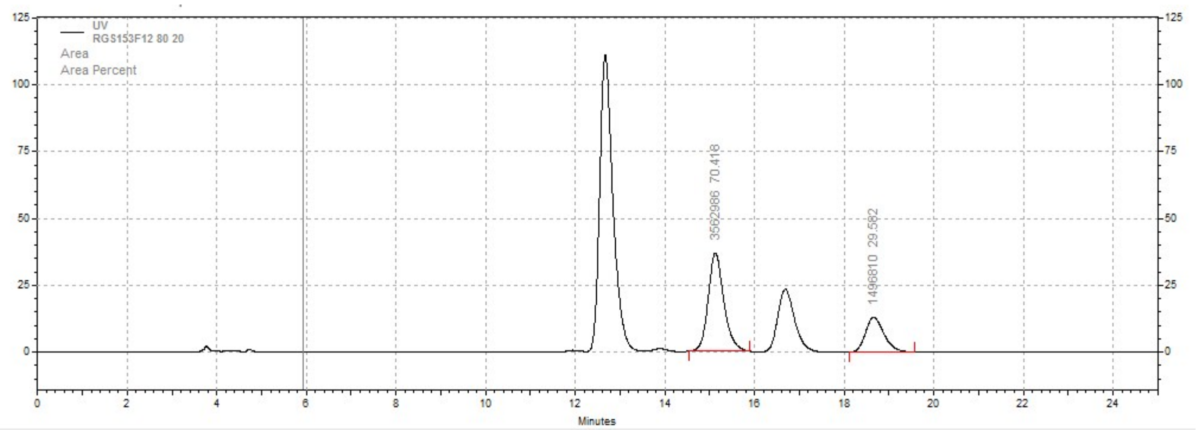

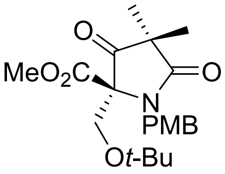

Non-racemic trace

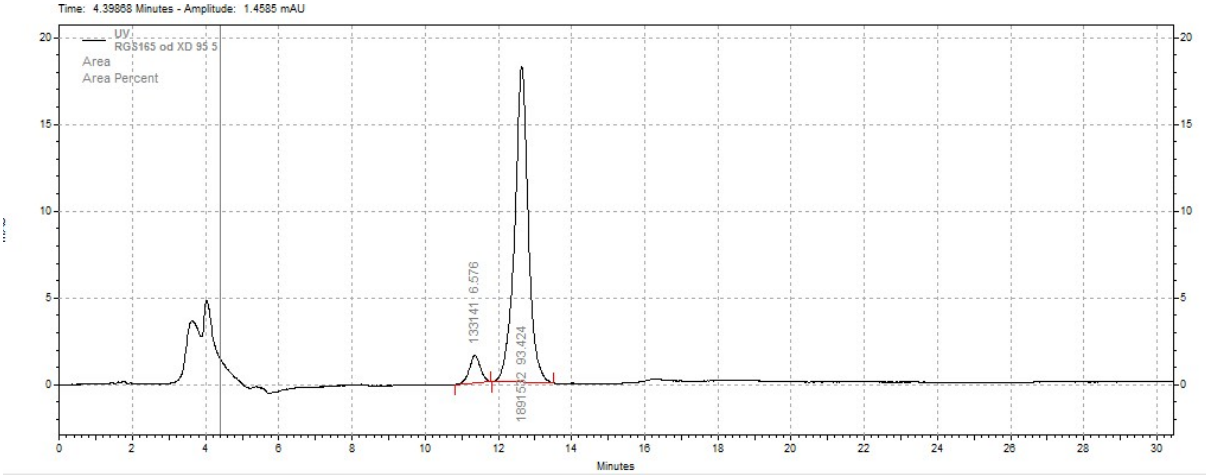

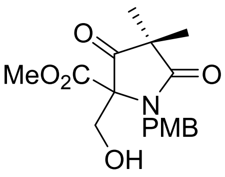

Racemic trace

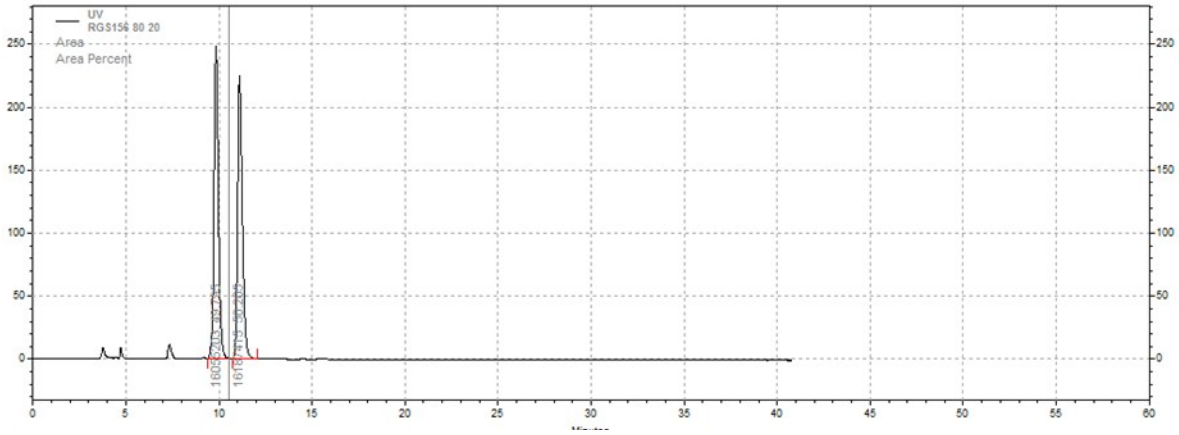

Non-racemic trace

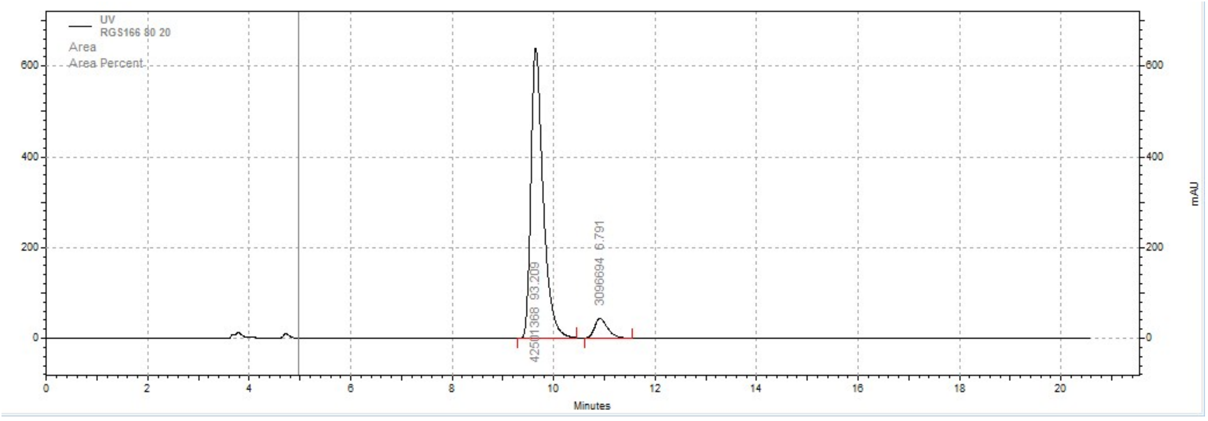

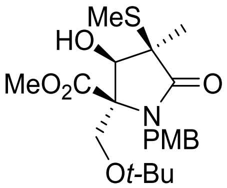

Racemic trace

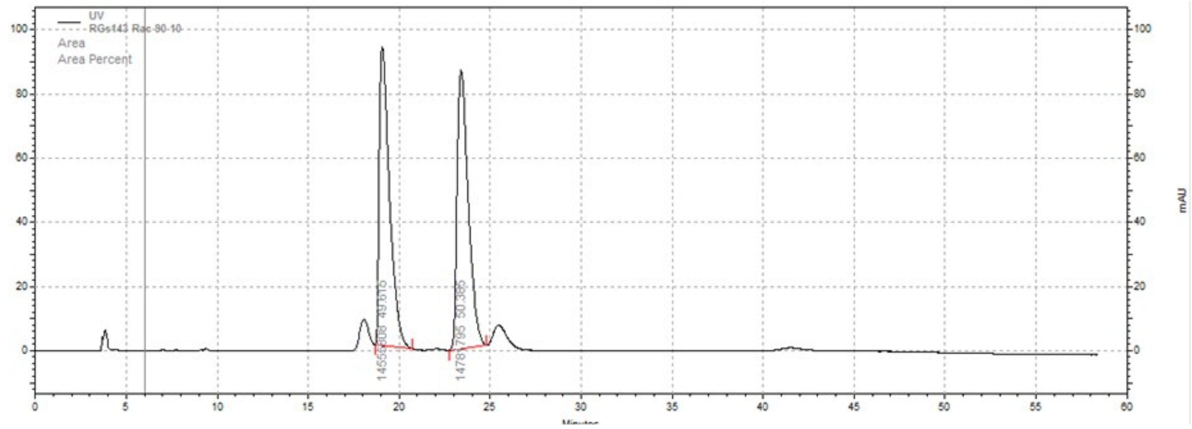

Non-racemic trace

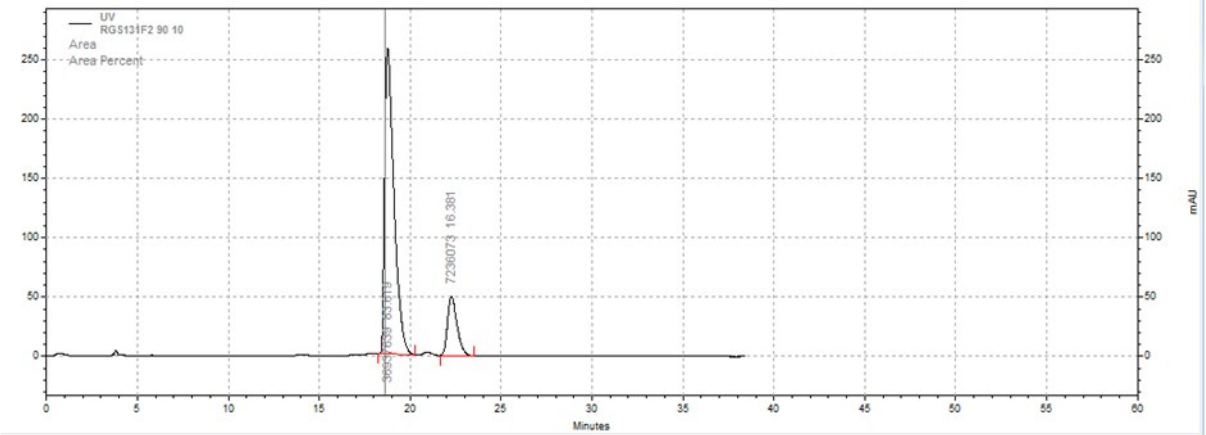

Trace of the supernatant after a recrystallisation from IPA

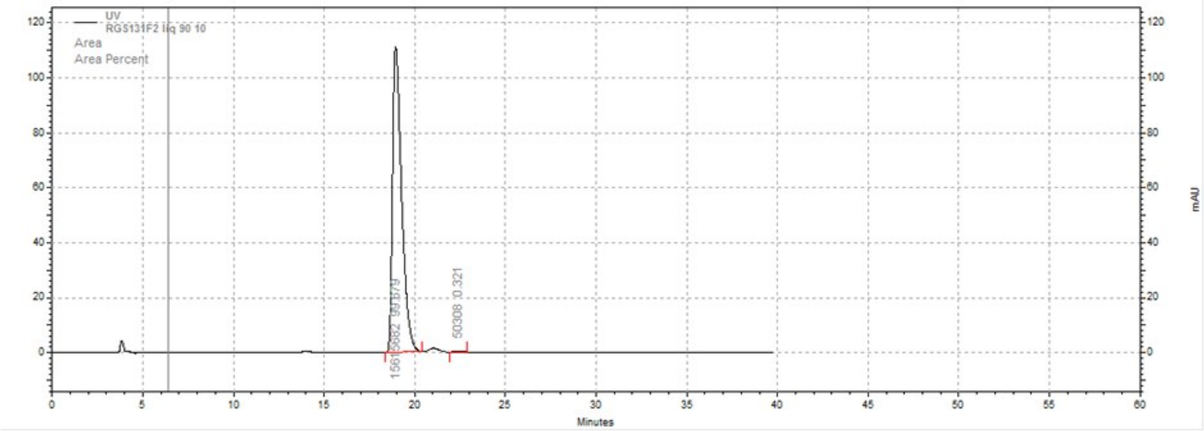

Trace of the crystals after a recrystallization from IPA

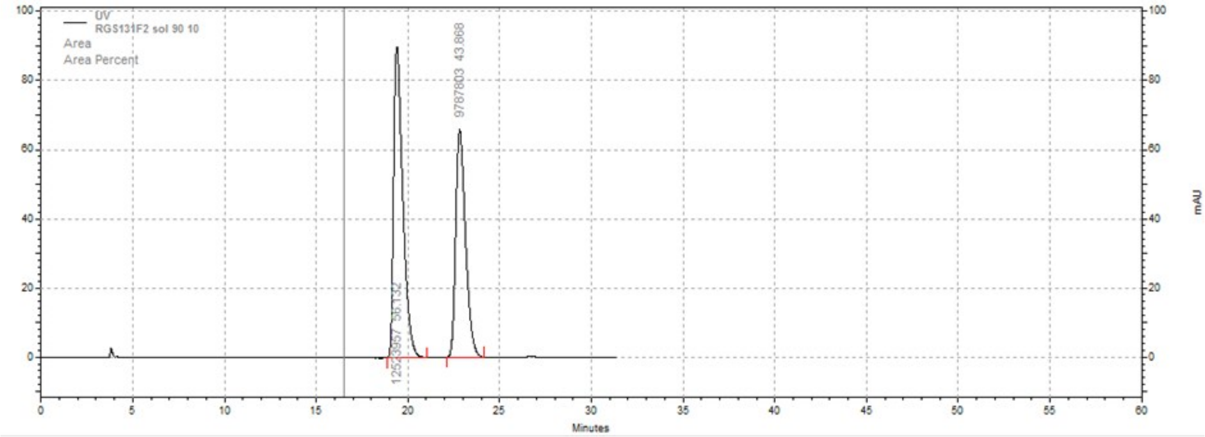

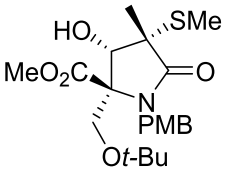

Racemic trace

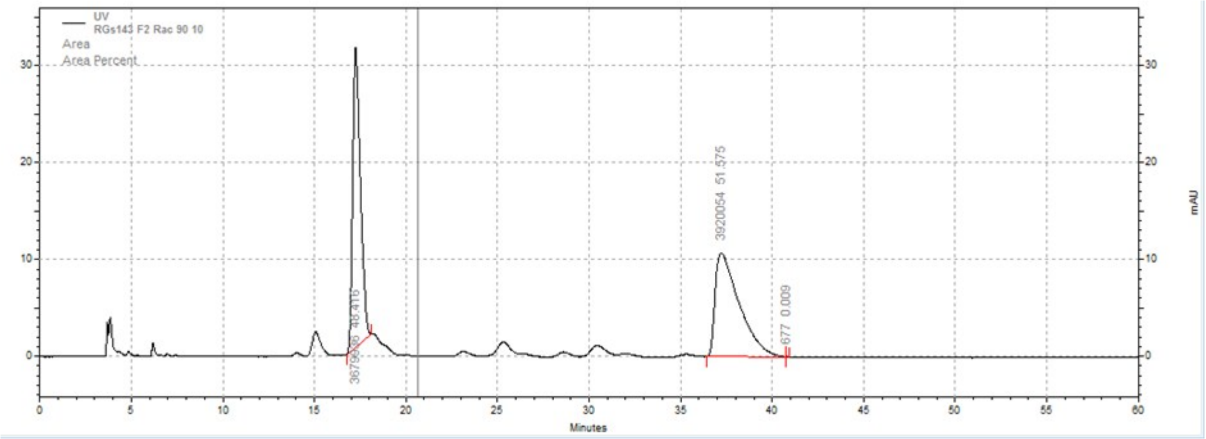

Non-racemic trace

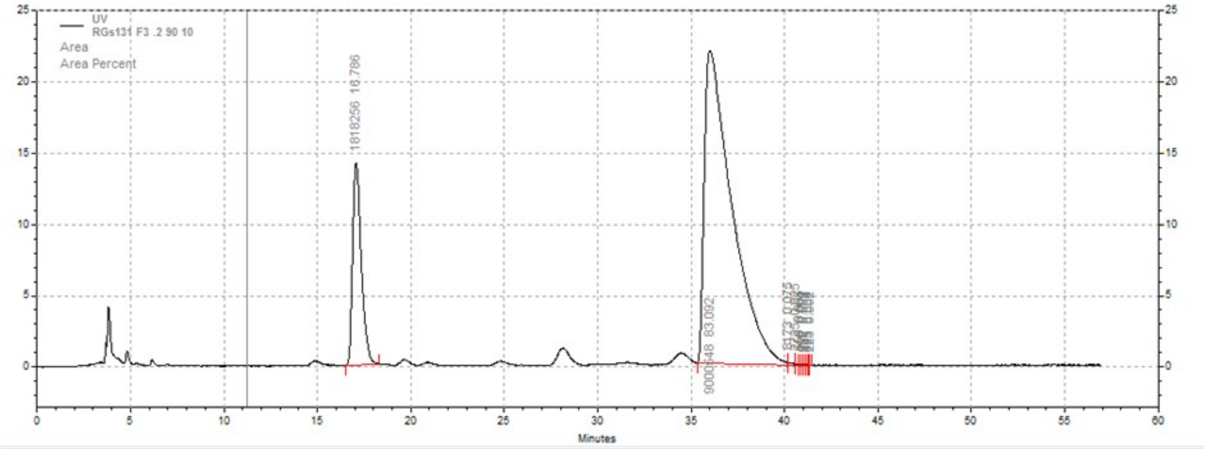

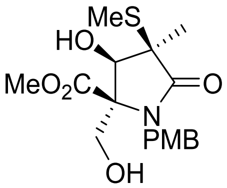

Racemic trace

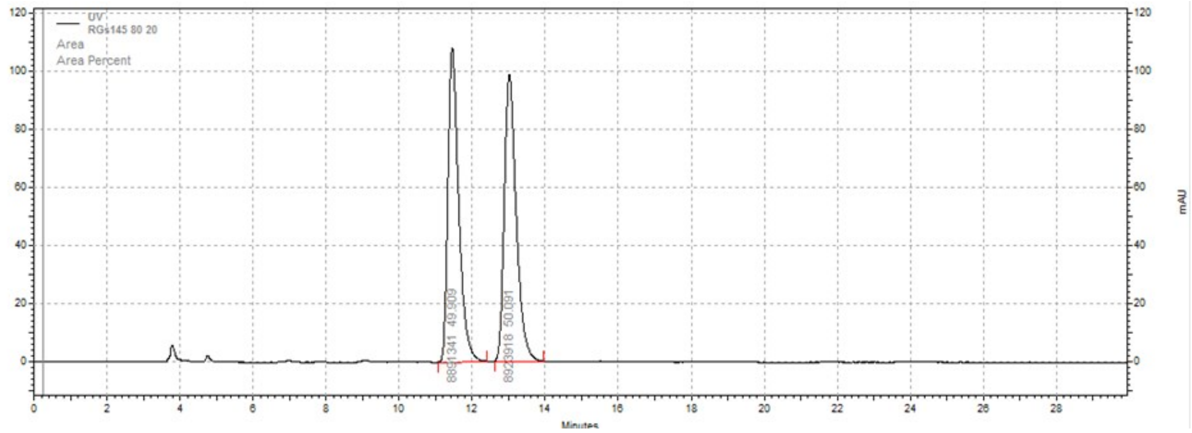

Non-racemic trace

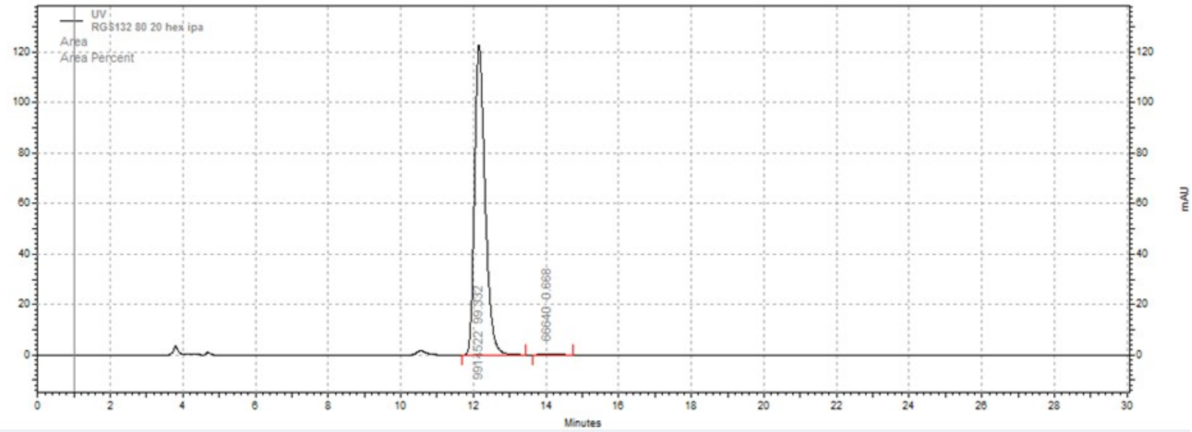

X RAY CRYTALLOGRAPHIC ESI

CCDC 1916072-1916076

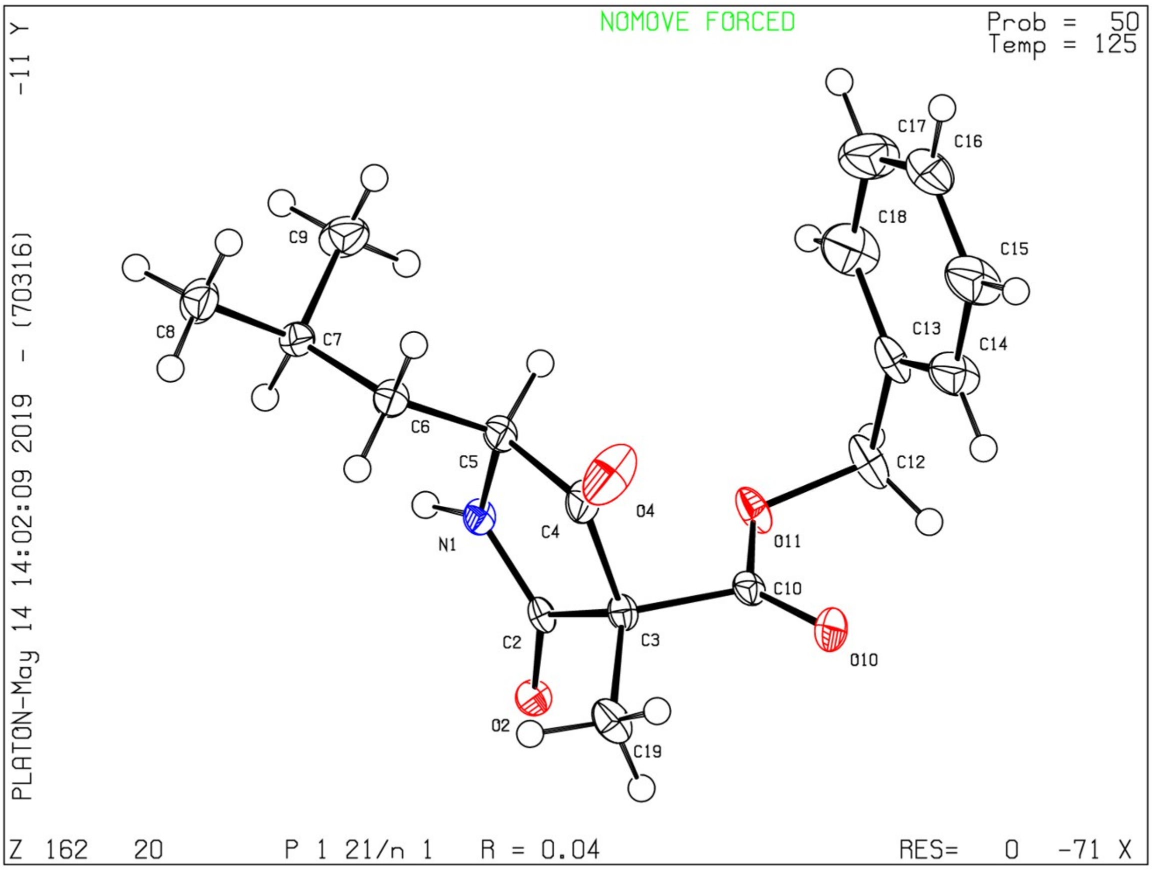
NOMOVE FORCED
Prob = 50
Temp = 125
-11 Y
PLATON-May 14 14:02:09 2019 - (70316)
C8
C9
C7
C6
C5
N1
C4
O4
C2
O2
C3
C19
C10
O10
O11
C12
C13
C14
C15
C16
C17
C18
Z 162 20
P 1 21/n 1 R = 0.04
RES= 0 -71 X

20

Data Collection 20/1916072

A colourless prism crystal of $C_{17}H_{21}NO_4$ having approximate dimensions of 0.120 x 0.050 x 0.030 mm was mounted in a loop. All measurements were made on a Rigaku XtaLAB P200 diffractometer using graphite monochromated Cu-Kα radiation.

Cell constants and an orientation matrix for data collection corresponded to a primitive monoclinic cell with dimensions:

a = 15.59060(16) Å

b = 5.83233(5) Å β = 112.9800(13)^{o}

c = 19.3142(2) Å

V = 1616.86(3) Å^3

For Z = 4 and F.W. = 303.36, the calculated density is 1.246 g/cm^3. The reflection conditions of:

h0l: h+l = 2n

0k0: k = 2n

uniquely determine the space group to be:

$P2_1/n$ (#14)

The data were collected at a temperature of -148 $\pm$ 1°C to a maximum 2θ value of 150.9^{o}.

Data Reduction

Of the 17824 reflections were collected, where 3279 were unique (R_{int} = 0.0247). Data were collected and processed using CrysAlisPro (Rigaku Oxford Diffraction). [1]

The linear absorption coefficient, μ, for Cu-Kα radiation is 7.268 cm^{-1}. An empirical absorption correction was applied which resulted in transmission factors ranging from 0.743 to 0.978. The data were corrected for Lorentz and polarization effects. A correction for secondary extinction[2] was applied (coefficient = 0.008530).

Structure Solution and Refinement

The structure was solved by direct methods[3] and expanded using Fourier techniques. The non-hydrogen atoms were refined anisotropically. Some hydrogen atoms were refined isotropically and the rest were refined using the riding model.The final cycle of full-matrix least-squares refinement[4] on F^2 was based on 3279 observed reflections and 204 variable parameters and converged (largest parameter shift was 0.00 times its esd) with unweighted and weighted agreement factors of:

$$R1 = \Sigma \,||Fo| - |Fc|| \,/\, \Sigma\, |Fo| = 0.0411$$

$$wR2 = [\,\Sigma\,(\,w\,(Fo^2 - Fc^2)^2\,)/\,\Sigma\, w(Fo^2)^2]^{1/2} = 0.1119$$

The goodness of fit[5] was 1.04. Unit weights were used. The maximum and minimum peaks on the final difference Fourier map corresponded to 0.35 and -0.22 $e^-/Å^3$, respectively.

Neutral atom scattering factors were taken from International Tables for Crystallography (IT), Vol. C, Table 6.1.1.4 [6]. Anomalous dispersion effects were included in Fcalc[7]; the values for $\Delta f'$ and $\Delta f''$ were those of Creagh and McAuley[8]. The values for the mass attenuation coefficients are those of Creagh and Hubbell[9]. All calculations were performed using the CrystalStructure[10] crystallographic software package except for refinement, which was performed using SHELXL Version 2014/7[11].

References

(1) CrysAlisPro: Data Collection and Processing Software, Rigaku Corporation (2015). Tokyo 196-8666, Japan.

(2) Larson, A.C. (1970), Crystallographic Computing, 291-294. F.R. Ahmed, ed. Munksgaard, Copenhagen (equation 22, with V replaced by the cell volume).

(3) SIR2011: Burla, M. C., Caliandro, R., Camalli, M., Carrozzini, B., Cascarano, G. L., Giacovazzo, C., Mallamo, M., Mazzone, A., Polidori, G. and Spagna, R. (2012). J. Appl. Cryst. 45, 357-361.

(4) Least Squares function minimized: (SHELXL Version 2014/7)

$\Sigma w(F_o^2 - F_c^2)^2$ where w = Least Squares weights.

(5) Goodness of fit is defined as:

$[\Sigma w(F_o^2 - F_c^2)^2/(N_o - N_v)]^{1/2}$

where: N_o = number of observations

N_V = number of variables

(6) International Tables for Crystallography, Vol.C (1992). Ed. A.J.C. Wilson, Kluwer Academic Publishers, Dordrecht, Netherlands, Table 6.1.1.4, pp. 572.

(7) Ibers, J. A. & Hamilton, W. C.; Acta Crystallogr., 17, 781 (1964).

(8) Creagh, D. C. & McAuley, W.J .; "International Tables for Crystallography", Vol C, (A.J.C. Wilson, ed.), Kluwer Academic Publishers, Boston, Table 4.2.6.8, pages 219-222 (1992).

(9) Creagh, D. C. & Hubbell, J.H..; "International Tables for Crystallography", Vol C, (A.J.C. Wilson, ed.), Kluwer Academic Publishers, Boston, Table 4.2.4.3, pages 200-206 (1992).

(10) CrystalStructure 4.2: Crystal Structure Analysis Package, Rigaku Corporation (2000-2015). Tokyo 196-8666, Japan.

(11) SHELXL Version 2014/7: Sheldrick, G. M. (2008). Acta Cryst. A64, 112-122.

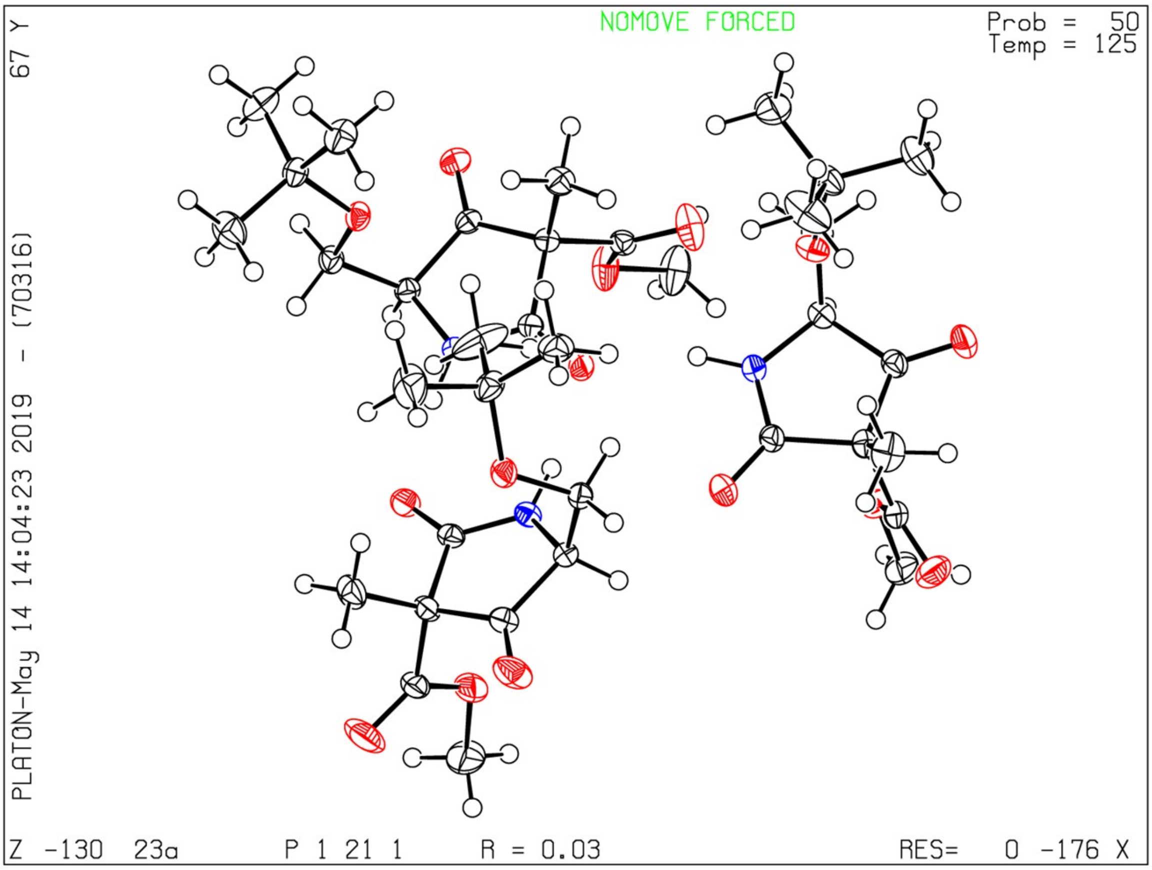
NOMOVE FORCED
Prob = 50
Temp = 125
67 Y
PLATON-May 14 14:04:23 2019 - (70316)
Z -130 23a
P 1 21 1
R = 0.03
RES= 0 -176 X

23a

Data Collection 23a/1916075

A colourless prism crystal of $C_{12}H_{19}NO_5$ having approximate dimensions of 0.200 x 0.200 x 0.200 mm was mounted in a loop. All measurements were made on a Rigaku XtaLAB P200 diffractometer using graphite monochromated Cu-Kα radiation.

Cell constants and an orientation matrix for data collection corresponded to a primitive monoclinic cell with dimensions:

a = 8.41466(6) Å

b = 22.42870(13) Å β = 107.7070(8)^{o}

c = 11.38440(9) Å

V = 2046.79(3) Å^3

For Z = 6 and F.W. = 257.29, the calculated density is 1.252 g/cm^3. Based on the reflection conditions of:

0k0: k = 2n

packing considerations, a statistical analysis of intensity distribution, and the successful solution and refinement of the structure, the space group was determined to be:

$P2_1$ (#4)

The data were collected at a temperature of -148 ± 1°C to a maximum 2θ value of 150.7^{o}.

Data Reduction

Of the 22771 reflections were collected, where 7799 were unique (R_{int} = 0.0113). Data were collected and processed using CrysAlisPro (Rigaku Oxford Diffraction). [1]

The linear absorption coefficient, μ, for Cu-Kα radiation is 8.185 cm^{-1}. An empirical absorption correction was applied which resulted in transmission factors ranging from 0.740 to 0.849. The data were corrected for Lorentz and polarization effects.

Structure Solution and Refinement

The structure was solved by direct methods[2] and expanded using Fourier techniques. The non-hydrogen atoms were refined anisotropically. Some hydrogen atoms were refined isotropically and the rest were refined using the riding model.The final cycle of full-matrix least-squares refinement[3] on F^2 was based on 7799 observed reflections and 499 variable parameters and converged (largest parameter shift was 0.00 times its esd) with unweighted and weighted agreement factors of:

$$R1 = \Sigma ||Fo| - |Fc|| / \Sigma |Fo| = 0.0272$$

$$wR2 = [\Sigma (w (Fo^2 - Fc^2)^2)/ \Sigma w(Fo^2)^2]^{1/2} = 0.0749$$

The goodness of fit[4] was 1.02. Unit weights were used. The maximum and minimum peaks on the final difference Fourier map corresponded to 0.22 and -0.18 $e^-/Å^3$, respectively. The final Flack parameter [5] was -0.01(5), indicating that the present absolute structure is correct. [6]

Neutral atom scattering factors were taken from International Tables for Crystallography (IT), Vol. C, Table 6.1.1.4 [7]. Anomalous dispersion effects were included in Fcalc[8]; the values for Δf' and Δf" were those of Creagh and McAuley[9]. The values for the mass attenuation coefficients are those of Creagh and Hubbell[10]. All calculations were performed using the CrystalStructure[11] crystallographic software package except for refinement, which was performed using SHELXL Version 2014/7[12].

References

(1) CrysAlisPro: Data Collection and Processing Software, Rigaku Corporation (2015). Tokyo 196-8666, Japan.

(2) SHELXT Version 2014/4: Sheldrick, G. M. (2014). Acta Cryst. A70, C1437.

(3) Least Squares function minimized: (SHELXL Version 2014/7)

$\Sigma w(F_o^2-F_c^2)^2$ where w = Least Squares weights.

(4) Goodness of fit is defined as:

$[\Sigma w(F_o^2-F_c^2)^2/(N_o-N_v)]^{1/2}$

where: N_o = number of observations

N_v = number of variables

(5) Parsons, S., Flack, H.D. and Wagner, T. Acta Cryst. B69 (2013) 249-259.

(6) Flack, H.D. and Bernardinelli (2000), J. Appl. Cryst. 33, 114-1148.

(7) International Tables for Crystallography, Vol.C (1992). Ed. A.J.C. Wilson, Kluwer Academic Publishers, Dordrecht, Netherlands, Table 6.1.1.4, pp. 572.

(8) Ibers, J. A. & Hamilton, W. C.; Acta Crystallogr., 17, 781 (1964).

(9) Creagh, D. C. & McAuley, W.J .; "International Tables for Crystallography", Vol C, (A.J.C. Wilson, ed.), Kluwer Academic Publishers, Boston, Table 4.2.6.8, pages 219-222 (1992).

(10) Creagh, D. C. & Hubbell, J.H..; "International Tables for Crystallography", Vol C, (A.J.C. Wilson, ed.), Kluwer Academic Publishers, Boston, Table 4.2.4.3, pages 200-206 (1992).

(11) CrystalStructure 4.3: Crystal Structure Analysis Package, Rigaku Corporation (2000-2018). Tokyo 196-8666, Japan.

(12) SHELXL Version 2014/7: Sheldrick, G. M. (2008). Acta Cryst. A64, 112-122.

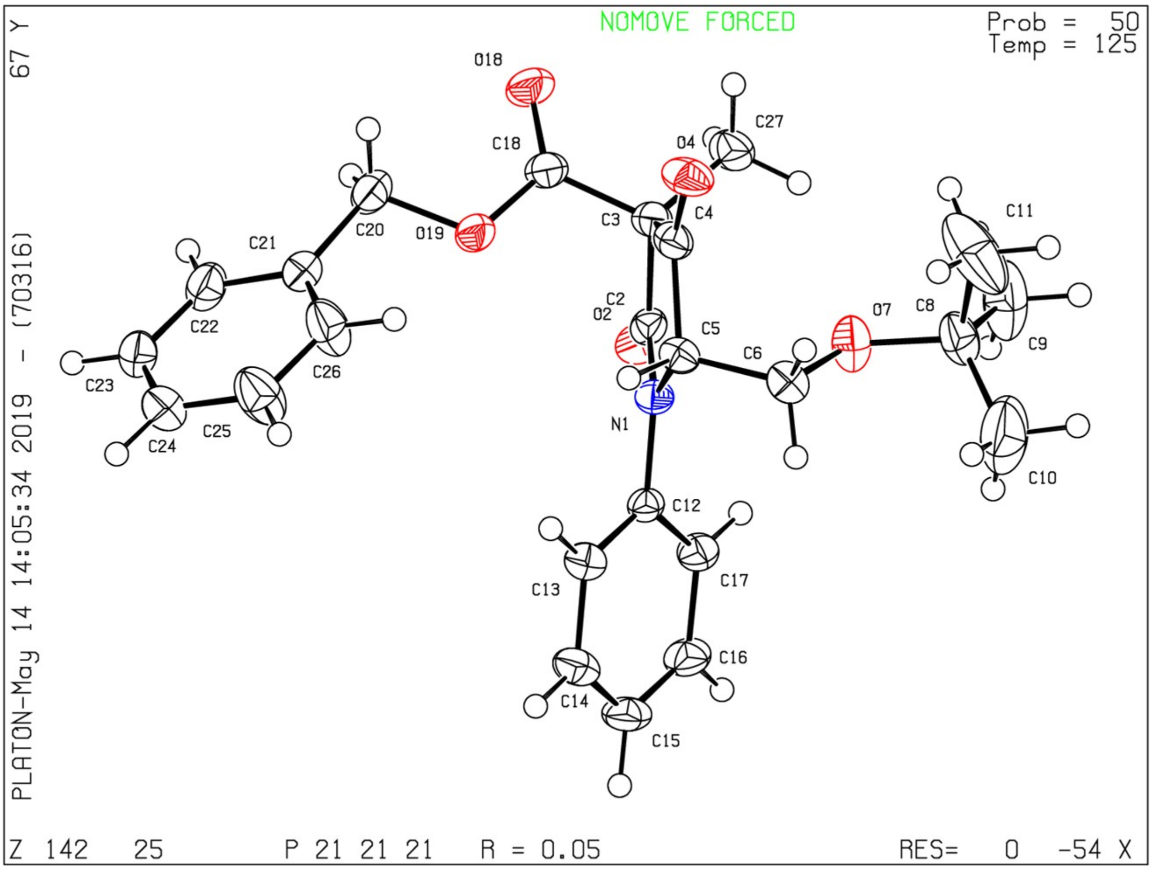

NOMOVE FORCED
Prob = 50
Temp = 125
67 Y
PLATON-May 14 14:05:34 2019 - (70316)
O18
C18
C27
O4
C20
O19
C3
C4
C21
C11
C22
C2
O2
C5
O7
C8
C6
C9
C23
C26
N1
C24
C25
C10
C12
C13
C17
C16
C14
C15
Z 142 25
P 21 21 21
R = 0.05
RES= 0 -54 X

25

Data Collection 25/1916076

A colourless prism crystal of $C_{24}H_{27}NO_5$ having approximate dimensions of 0.200 x 0.020 x 0.020 mm was mounted in a loop. All measurements were made on a Rigaku XtaLAB P200 diffractometer using graphite monochromated Cu-Kα radiation.

Cell constants and an orientation matrix for data collection corresponded to a primitive orthorhombic cell with dimensions:

a = 6.24701(5) Å

b = 17.78990(14) Å

c = 20.26860(16) Å

V = 2252.52(3) Å^3

For Z = 4 and F.W. = 409.48, the calculated density is 1.207 g/cm^3. The reflection conditions of:

h00: h = 2n

0k0: k = 2n

00l: l = 2n

uniquely determine the space group to be:

$P2_12_12_1$ (#19)

The data were collected at a temperature of -148 $\pm$ 1°C to a maximum 2θ value of 151.1^{o}.

Data Reduction

Of the 26719 reflections were collected, where 4594 were unique (R_{int} = 0.0203). Data were collected and processed using CrysAlisPro (Rigaku Oxford Diffraction). [1]

The linear absorption coefficient, μ, for Cu-Kα radiation is 6.885 cm^{-1}. An empirical absorption correction was applied which resulted in transmission factors ranging from 0.726 to 0.986. The data were corrected for Lorentz and polarization effects.

Structure Solution and Refinement

The structure was solved by direct methods[2] and expanded using Fourier techniques. The non-hydrogen atoms were refined anisotropically. Hydrogen atoms were refined using the riding model. The final cycle of full-matrix least-squares refinement[3] on F^2 was based on 4594 observed reflections and 271 variable parameters and converged (largest parameter shift was 0.00 times its esd) with unweighted and weighted agreement factors of:

$$R1 = \Sigma \,||Fo| - |Fc|| \,/\, \Sigma\, |Fo| = 0.0454$$

$$wR2 = [\, \Sigma\, (w (Fo^2 - Fc^2)^2)/ \Sigma\, w(Fo^2)^2]^{1/2} = 0.1331$$

The goodness of fit[4] was 1.06. Unit weights were used. The maximum and minimum peaks on the final difference Fourier map corresponded to 0.80 and -0.35 $e^-/Å^3$, respectively. The final Flack parameter [5] was -0.00(4), indicating that the present absolute structure is correct. [6]

Neutral atom scattering factors were taken from International Tables for Crystallography (IT), Vol. C, Table 6.1.1.4 [7]. Anomalous dispersion effects were included in Fcalc[8]; the values for Δf' and Δf" were those of Creagh and McAuley[9]. The values for the mass attenuation coefficients are those of Creagh and Hubbell[10]. All calculations were performed using the CrystalStructure[11] crystallographic software package except for refinement, which was performed using SHELXL Version 2014/7[12].

References

(1) CrysAlisPro: Data Collection and Processing Software, Rigaku Corporation (2015). Tokyo 196-8666, Japan.

(2) SHELXT Version 2014/4: Sheldrick, G. M. (2014). Acta Cryst. A70, C1437.

(3) Least Squares function minimized: (SHELXL Version 2014/7)

$\Sigma w(F_o^2-F_c^2)^2$ where w = Least Squares weights.

(4) Goodness of fit is defined as:

$[\Sigma w(F_o^2-F_c^2)^2/(N_o-N_v)]^{1/2}$

where: N_o = number of observations

N_v = number of variables

(5) Parsons, S., Flack, H.D. and Wagner, T. Acta Cryst. B69 (2013) 249-259.

(6) Flack, H.D. and Bernardinelli (2000), J. Appl. Cryst. 33, 114-1148.

(7) International Tables for Crystallography, Vol.C (1992). Ed. A.J.C. Wilson, Kluwer Academic Publishers, Dordrecht, Netherlands, Table 6.1.1.4, pp. 572.

(8) Ibers, J. A. & Hamilton, W. C.; Acta Crystallogr., 17, 781 (1964).

(9) Creagh, D. C. & McAuley, W.J .; "International Tables for Crystallography", Vol C, (A.J.C. Wilson, ed.), Kluwer Academic Publishers, Boston, Table 4.2.6.8, pages 219-222 (1992).

(10) Creagh, D. C. & Hubbell, J.H..; "International Tables for Crystallography", Vol C, (A.J.C. Wilson, ed.), Kluwer Academic Publishers, Boston, Table 4.2.4.3, pages 200-206 (1992).

(11) CrystalStructure 4.3: Crystal Structure Analysis Package, Rigaku Corporation (2000-2018). Tokyo 196-8666, Japan.

(12) SHELXL Version 2014/7: Sheldrick, G. M. (2008). Acta Cryst. A64, 112-122.

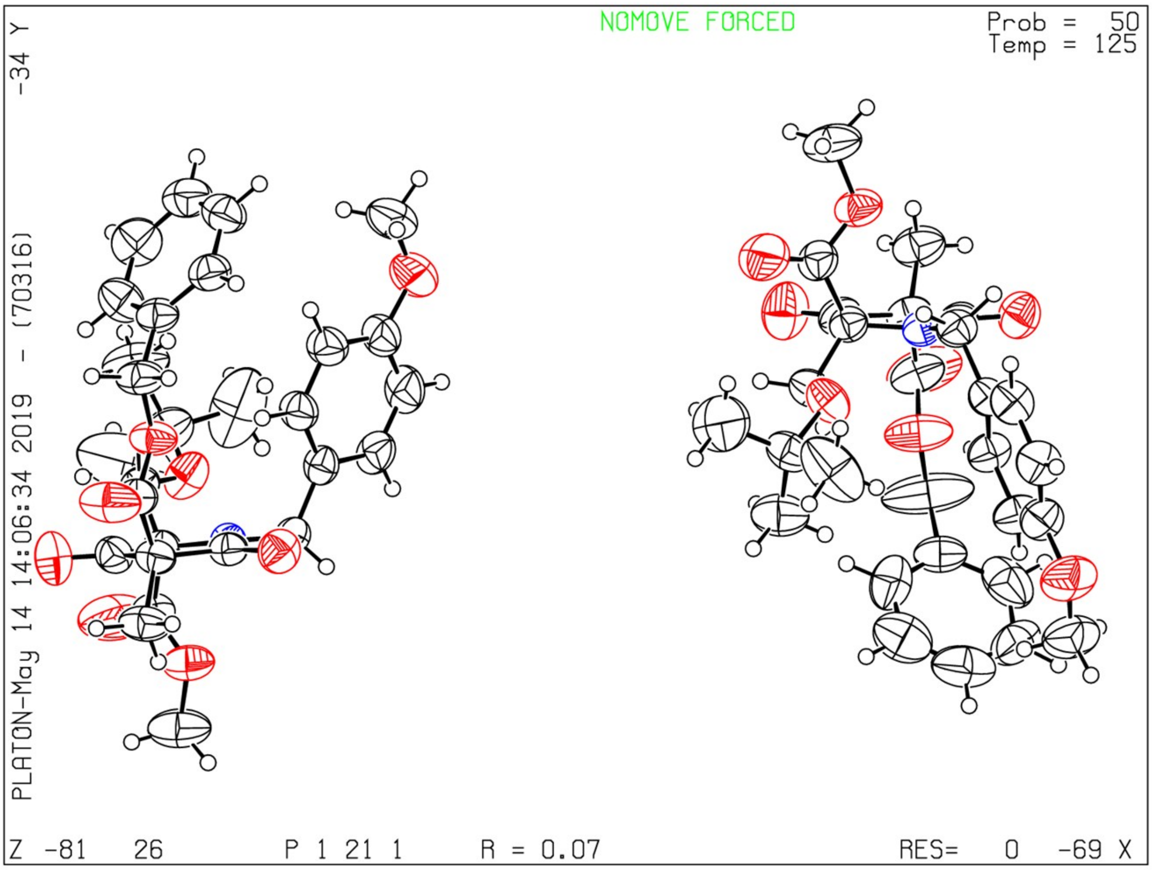
NOMOVE FORCED
Prob = 50
Temp = 125
-34 Y
PLATON-May 14 14:06:34 2019 - (70316)
Z -81 26
P 1 21 1
R = 0.07
RES= 0 -69 X

26

Data Collection 26/1916073

A colourless prism crystal of $C_{28}H_{33}NO_8$ having approximate dimensions of 0.200 x 0.100 x 0.100 mm was mounted in a loop. All measurements were made on a Rigaku XtaLAB P200 diffractometer using graphite monochromated Cu-Kα radiation.

Cell constants and an orientation matrix for data collection corresponded to a primitive monoclinic cell with dimensions:

a = 10.3313(2) Å

b = 10.2519(2) Å β = 98.891(2)^{o}

c = 26.4465(6) Å

V = 2767.44(10) Å^3

For Z = 4 and F.W. = 511.57, the calculated density is 1.228 g/cm^3. Based on the reflection conditions of:

0k0: k = 2n

packing considerations, a statistical analysis of intensity distribution, and the successful solution and refinement of the structure, the space group was determined to be:

$P2_1$ (#4)

The data were collected at a temperature of -148 $\pm$ 1°C to a maximum 2θ value of 136.4^{o}.

Data Reduction

Of the 22931 reflections were collected, where 9321 were unique (R_{int} = 0.0295). Data were collected and processed using CrysAlisPro (Rigaku Oxford Diffraction). [1]

The linear absorption coefficient, μ, for Cu-Kα radiation is 7.454 cm^{-1}. An empirical absorption correction was applied which resulted in transmission factors ranging from 0.772 to 0.928. The data were corrected for Lorentz and polarization effects.

Structure Solution and Refinement

The structure was solved by direct methods[2] and expanded using Fourier techniques. The non-hydrogen atoms were refined anisotropically. Hydrogen atoms were refined using the riding model. The final cycle of full-matrix least-squares refinement[3] on F^2 was based on 9321 observed reflections and 667 variable parameters and converged (largest parameter shift was 0.00 times its esd) with unweighted and weighted agreement factors of:

$$R1 = \Sigma \, ||Fo| - |Fc|| \, / \, \Sigma \, |Fo| = 0.0691$$

$$wR2 = [\, \Sigma \, (w (Fo^2 - Fc^2)^2) / \, \Sigma \, w(Fo^2)^2]^{1/2} = 0.2038$$

The goodness of fit[4] was 1.02. Unit weights were used. The maximum and minimum peaks on the final difference Fourier map corresponded to 0.30 and -0.23 e^{-}/Å^{3}, respectively. The final Flack parameter [5] was 0.06(8), indicating that the present absolute structure is correct. [6]

Neutral atom scattering factors were taken from International Tables for Crystallography (IT), Vol. C, Table 6.1.1.4 [7]. Anomalous dispersion effects were included in Fcalc[8]; the values for Δf' and Δf" were those of Creagh and McAuley[9]. The values for the mass attenuation coefficients are those of Creagh and Hubbell[10]. All calculations were performed using the CrystalStructure[11] crystallographic software package except for refinement, which was performed using SHELXL Version 2014/7[12].

References

(1) CrysAlisPro: Data Collection and Processing Software, Rigaku Corporation (2015). Tokyo 196-8666, Japan.

(2) SHELXT Version 2014/4: Sheldrick, G. M. (2014). Acta Cryst. A70, C1437.

(3) Least Squares function minimized: (SHELXL Version 2014/7)

$$\Sigma w(F_o^2-F_c^2)^2 \quad \text{where w = Least Squares weights.}$$

(4) Goodness of fit is defined as:

$$[\Sigma w(F_o^2-F_c^2)^2/(N_o-N_v)]^{1/2}$$

where: N_o = number of observations

N_v = number of variables

(5) Parsons, S., Flack, H.D. and Wagner, T. Acta Cryst. B69 (2013) 249-259.

(6) Flack, H.D. and Bernardinelli (2000), J. Appl. Cryst. 33, 114-1148.

(7) International Tables for Crystallography, Vol.C (1992). Ed. A.J.C. Wilson, Kluwer Academic Publishers, Dordrecht, Netherlands, Table 6.1.1.4, pp. 572.

(8) Ibers, J. A. & Hamilton, W. C.; Acta Crystallogr., 17, 781 (1964).

(9) Creagh, D. C. & McAuley, W.J .; "International Tables for Crystallography", Vol C, (A.J.C. Wilson, ed.), Kluwer Academic Publishers, Boston, Table 4.2.6.8, pages 219-222 (1992).

(10) Creagh, D. C. & Hubbell, J.H..; "International Tables for Crystallography", Vol C, (A.J.C. Wilson, ed.), Kluwer Academic Publishers, Boston, Table 4.2.4.3, pages 200-206 (1992).

(11) CrystalStructure 4.2: Crystal Structure Analysis Package, Rigaku Corporation (2000-2015). Tokyo 196-8666, Japan.

(12) SHELXL Version 2014/7: Sheldrick, G. M. (2008). Acta Cryst. A64, 112-122.

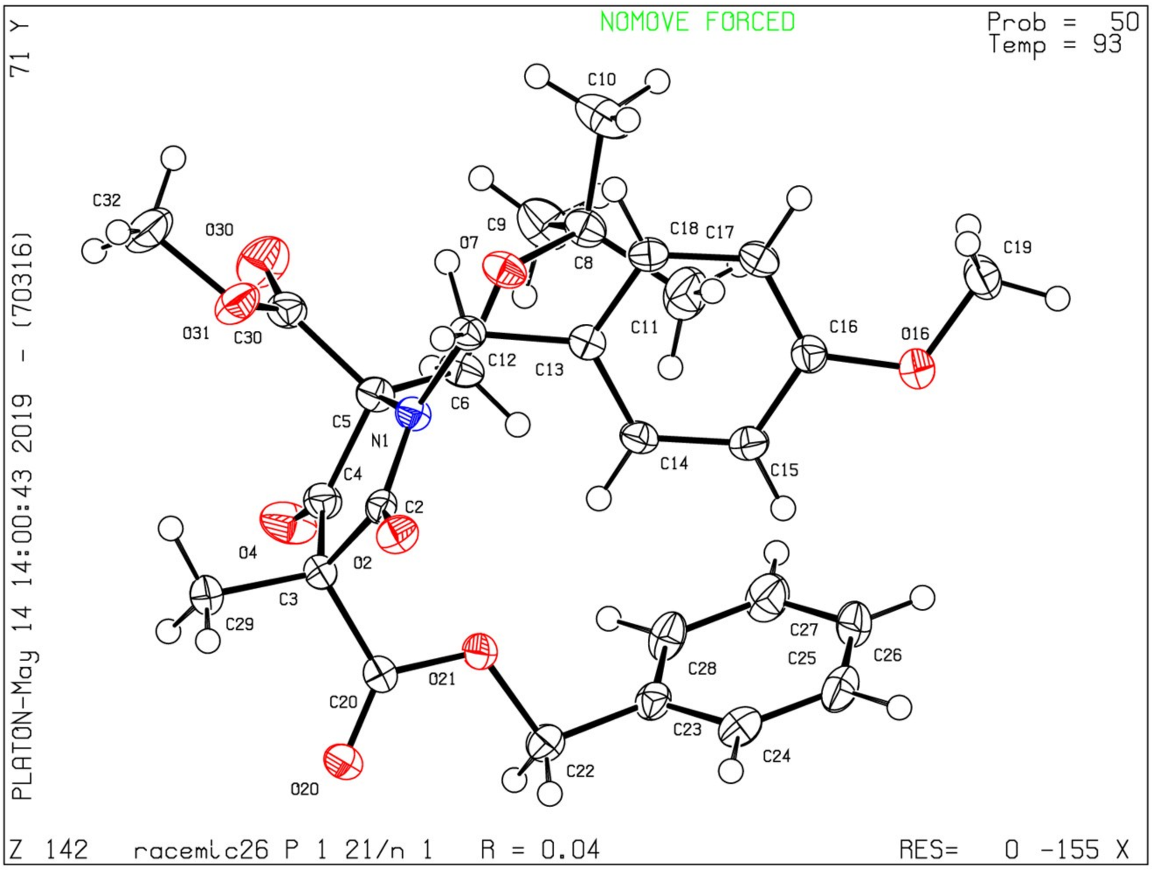
NOMOVE FORCED
Prob = 50
Temp = 93
71 Y
PLATON-May 14 14:00:43 2019 - (70316)
Z 142 racemic26 P 1 21/n 1 R = 0.04
RES= 0 -155 X
C10
C32
O30
C9
C8
C18
C17
C19
O7
O31
C30
C12
C13
C11
C16
O16
C6
C5
N1
C14
C15
C4
C2
O4
O2
C3
C29
C27
C25
C26
C28
O21
C20
C23
C24
C22
O20

+- 26

Data Collection +-26/1916074

A colourless prism crystal of $C_{28}H_{33}NO_8$ having approximate dimensions of 0.200 x 0.200 x 0.200 mm was mounted in a loop. All measurements were made on a Rigaku XtaLAB P200 diffractometer using graphite monochromated Cu-Kα radiation.

Cell constants and an orientation matrix for data collection corresponded to a primitive monoclinic cell with dimensions:

a = 14.29830(9) Å

b = 10.41200(6) Å β = 105.0690(6)^{o}

c = 18.66340(11) Å

V = 2682.95(3) Å^3

For Z = 4 and F.W. = 511.57, the calculated density is 1.266 g/cm^3. The reflection conditions of:

h0l: h+l = 2n

0k0: k = 2n

uniquely determine the space group to be:

$P2_1/n$ (#14)

The data were collected at a temperature of -180 $\pm$ 1°C to a maximum 2θ value of 150.9^{o}.

Data Reduction

Of the 29951 reflections were collected, where 5454 were unique (R_{int} = 0.0195). Data were collected and processed using CrysAlisPro (Rigaku Oxford Diffraction). [1]

The linear absorption coefficient, μ, for Cu-Kα radiation is 7.689 cm^{-1}. An empirical absorption correction was applied which resulted in transmission factors ranging from 0.700 to 0.857. The data were corrected for Lorentz and polarization effects. A correction for secondary extinction[2] was applied (coefficient = 0.012690).

Structure Solution and Refinement

The structure was solved by direct methods[3] and expanded using Fourier techniques. The non-hydrogen atoms were refined anisotropically. Hydrogen atoms were refined using the riding model. The final cycle of full-matrix least-squares refinement[4] on F^2 was based on 5454 observed reflections and 335 variable parameters and converged (largest parameter shift was 0.00 times its esd) with unweighted and weighted agreement factors of:

$$R1 = \Sigma \,||Fo| - |Fc|| \,/\, \Sigma \,|Fo| = 0.0437$$

$$wR2 = [\, \Sigma \,(w (Fo^2 - Fc^2)^2)/ \Sigma \,w(Fo^2)^2]^{1/2} = 0.1120$$

The goodness of fit[5] was 1.08. Unit weights were used. The maximum and minimum peaks on the final difference Fourier map corresponded to 0.34 and -0.29 $e^-/Å^3$, respectively.

Neutral atom scattering factors were taken from International Tables for Crystallography (IT), Vol. C, Table 6.1.1.4 [6]. Anomalous dispersion effects were included in Fcalc[7]; the values for Δf' and Δf" were those of Creagh and McAuley[8]. The values for the mass attenuation coefficients are those of Creagh and Hubbell[9]. All calculations were performed using the CrystalStructure[10] crystallographic software package except for refinement, which was performed using SHELXL Version 2014/7[11].

References

(1) CrysAlisPro: Data Collection and Processing Software, Rigaku Corporation (2015). Tokyo 196-8666, Japan.

(2) Larson, A.C. (1970), Crystallographic Computing, 291-294. F.R. Ahmed, ed. Munksgaard, Copenhagen (equation 22, with V replaced by the cell volume).

(3) SIR2011: Burla, M. C., Caliandro, R., Camalli, M., Carrozzini, B., Cascarano, G. L., Giacovazzo, C., Mallamo, M., Mazzone, A., Polidori, G. and Spagna, R. (2012). J. Appl. Cryst. 45, 357-361.

(4) Least Squares function minimized: (SHELXL Version 2014/7)

$\Sigma w(F_o^2-F_c^2)^2$ where w = Least Squares weights.

(5) Goodness of fit is defined as:

$[\Sigma w(F_o^2-F_c^2)^2/(N_o-N_v)]^{1/2}$

where: N_o = number of observations

N_V = number of variables

(6) International Tables for Crystallography, Vol.C (1992). Ed. A.J.C. Wilson, Kluwer Academic Publishers, Dordrecht, Netherlands, Table 6.1.1.4, pp. 572.

(7) Ibers, J. A. & Hamilton, W. C.; Acta Crystallogr., 17, 781 (1964).

(8) Creagh, D. C. & McAuley, W.J .; "International Tables for Crystallography", Vol C, (A.J.C. Wilson, ed.), Kluwer Academic Publishers, Boston, Table 4.2.6.8, pages 219-222 (1992).

(9) Creagh, D. C. & Hubbell, J.H..; "International Tables for Crystallography", Vol C, (A.J.C. Wilson, ed.), Kluwer Academic Publishers, Boston, Table 4.2.4.3, pages 200-206 (1992).

(10) CrystalStructure 4.2: Crystal Structure Analysis Package, Rigaku Corporation (2000-2015). Tokyo 196-8666, Japan.

(11) SHELXL Version 2014/7: Sheldrick, G. M. (2008). Acta Cryst. A64, 112-122.
